# Zinc-Mediated Four-Component Carbonylation toward Direct Synthesis of α‑Amino Ketones

**DOI:** 10.1021/acs.orglett.6c00604

**Published:** 2026-03-03

**Authors:** Qiangwei Li, Xiao-Feng Wu

**Affiliations:** † Dalian National Laboratory for Clean Energy, 58279 Dalian Institute of Chemical Physics, Chinese Academy of Sciences, 116023 Dalian, Liaoning, China; ‡ Leibniz-Institut für Katalyse e.V., Albert-Einstein-Strasse 29a, 18059 Rostock, Germany

## Abstract

α-Amino ketone plays an important role in medicinal chemistry due to its excellent multiple reaction sites, which serve as a chemical hub for constructing complex molecules. Moreover, its skeleton is also found in many bioactive molecules. The traditional methods for synthesizing α-amino ketones are usually limited by harsh reaction conditions and the use of presynthesized and toxic reagents. As a readily available synthetic reagent, organic iodides play an important role in organic synthesis due to their high reactivity and high functional group compatibility. Herein, we report a zinc-mediated carbonylation reaction involving alkyl iodides, aldehydes, and amines, which efficiently synthesized a series of α-amino ketone compounds.

## Graphical abstract

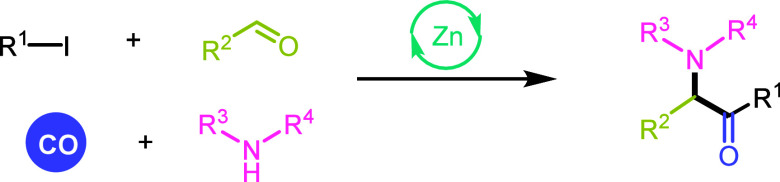

The α-amino ketone skeleton exists in many bioactive molecules, natural products, and drug molecules and is an important unit in organic synthesis.^1^ In 1850, Strecker^2^ reported the reaction of aldehydes and amines, which were then hydrolyzed under the action of sodium cyanide to produce α-amino acids. From the 1950s to the 1980s, chemists discovered that the SN2 substitution reaction between α-halogenated ketones and primary amines could yield α-amino ketones in a mild manner.^3^ Gradually, with the emergence of C–H functionalization and radical-mediated photochemistry and electrochemistry,^4^ the synthetic pathways of α-amino ketones have been greatly enriched, and their structural modifications have also gradually improved along with the development of chiral synthesis.

As a typical type of reaction for synthesizing ketone compounds, carbonylation has been recognized as a potent tool box for the synthesis of various carboxylic acid derivatives.^5^ Traditional carbonylation reactions are usually catalyzed by transition metals such as palladium and rhodium.^6^ In recent years, carbonylation reactions catalyzed by earth-abundant metals such as nickel, cobalt, and copper have gradually been discovered.^7^ This makes the synthesis of carbonyl-containing compounds greener than before. Alkyl halides are an important class of synthons in organic synthesis and are ubiquitously utilized in various carbon chain growth reactions (Scheme 1A).^8^ In recent years, carbonylation reactions in which alkyl halides participate have also been developed rapidly, covering the construction of various carbonylated skeletons such as esters,^9^ amides,^10^ ketones,^11^ and anhydrides,^12^ which have shown outstanding advantages such as mild conditions, wide functional group tolerance, and high chemical selectivity. However, the method of synthesizing α-amino ketones starting from alkyl iodine has rarely been reported. In 2021, we published an example of a carbonylation reaction between alkyl iodine and imine catalyzed by copper, which afforded a series of α-amino ketones in moderate yields.^13^ Procedures based on Hantzsch esters or alkylboronic acids under light irradiation were also developed.^14^ However, its related low atom efficiency has been considered as one of the drawbacks. Hence, although many great achievements have been realized, there is still a need to explore more efficient and convenient synthetic methods for the preparation of α-amino ketones. Herin, we propose a method for the carbonylation of alkyl iodides, aldehydes, and amines to produce α-amino ketones mediated by zinc under the action of a Lewis acid (Scheme 1B). The reaction conditions are mild and can occur at room temperature. Twenty bar of carbon monoxide is required to obtain the target product in moderate to good yields.

**Scheme 1:**
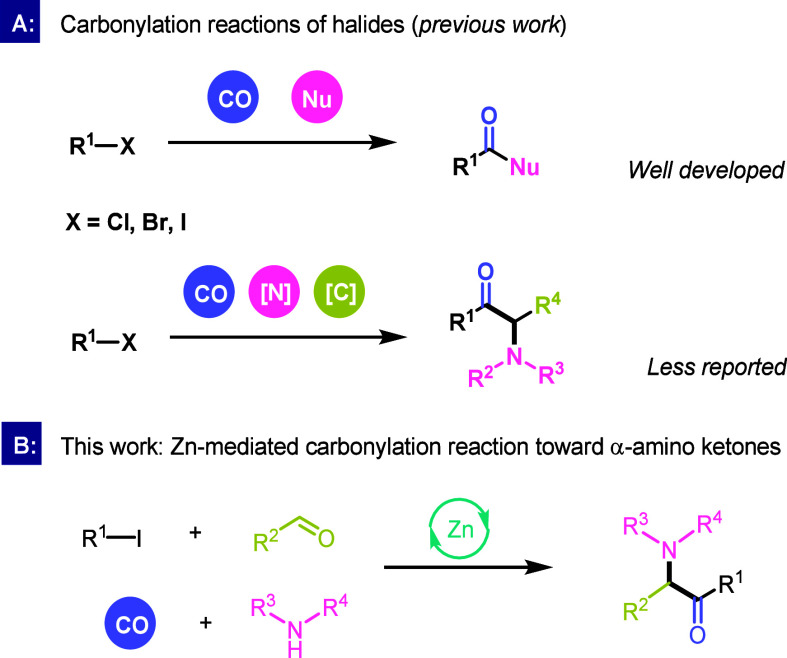
Synthesis of α-Amino Ketones

At the beginning of our investigation, cyclohexyliodine **1a**, phenylpropionaldehyde **2a**, and piperidine **3a** were selected as the model substrates for condition screening. Solvents play a decisive role in chemical reactions, so we first tested the solvent of this protocol. The results showed that this transformation could take place in most solvents, but the best result was achieved in 1,2-dimethoxyethane (DME), with a ≤72% yield. Considering that the reaction is mediated by metallic zinc, the dosage of zinc is also crucial. We then conducted a test on the use of zinc. When zinc was added, its amount gradually increased from 2 to 4 equiv, and the yield improved from 14% to 60%, respectively. When the amount continued to increase to 5 equiv, the yield increased by only 1%. Hence, we decided to use 4 equiv of zinc for the subsequent reaction. The proportion of each raw material used also affects the yield. When 1 equiv of **1a** was used, the yields of **2a** and **3a** were relatively low, only 8% and 15%, respectively (Table 1, entry 11). However, when 1 equiv of **2a** was used along with 3 and 1.5 equiv of **1a** and **3a**, respectively, the yield reached 66%. We then tested the effect of solvent usage on yield. The results showed that as the amount of solvent increased from 0.5 to 2.0 mL, the yield gradually decreased from 66% to 39%, respectively (Table 1, entry 5). We ultimately decided to conduct the experiment using 1 mL of DME. In order to check the effect of water, 5 equiv of H_2_O was added, the reaction was fully inhibited, which might be due to the failure of iminium intermediate formation (Table 1, entry 6). We also tried some other Lewis acids, such as TBSOTf (Table 1, entry 7), TMSCl, etc., but the yield was not improved. A 70% yield was obtained when the pressure was increased to 60 bar (Table 1, entry 9). When the pressure was further reduced to 5 bar, the yield decreased to 41% (Table 1, entry 8). This indicates that a high voltage is not a necessary condition for this protocol. When the pressure was 60 bar, we also attempted to increase the reaction temperature from 27 to 75 °C; however, the yield did not increase but decreased instead (Table 1, entry 10). We speculated that the high temperature might have affected the catalytic performance of the Lewis acid and favored the decarbonylation of the intermediate. We also attempted to increase the number of equivalents of water to provide hydrogen protons, but the result was that the conversion could not proceed. Finally, we attempted to shorten the reaction time. The yield did not decrease with a reduction in time. Eventually, an 81% yield was achieved in 12 h.

**Table 1:**
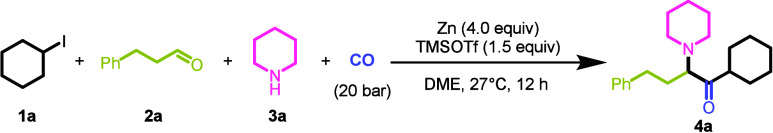
Optimization of the Reaction Conditions

entry^a^	modifications	yield (%)
1	none	81 (80)^b^
2	DMF as the solvent	5
3	EA as the solvent	54
4	2.0 equiv of Zn	14
5	2 mL of DME	39
6	5 equiv of H_2_O	0
7	TBSOTf instead of TMSOTf	22
8	5 bar of CO instead of 10 bar of CO	41
9	60 bar of CO instead of 10 bar of CO	70
10^c^	60 bar of CO instead of 10 bar of CO	34
11^d^	0.1 mmol of **1a**	15

a Reaction conditions: **1a** (0.3 mmol), **2a** (0.1 mmol), **3a** (0.15 mmol), Zn (4.0 equiv), TMSOTf (1.5 equiv), DME (1 mL), CO (20 bar), 27 °C. Yields were determined by GC-FID analysis using dodecane as the internal standard. Abbreviations: TMSOTf, trimethylsilyl trifluoromethanesulfonate; TBSOTf, *tert*-butyldimethylsilyl trifluoromethanesulfonate; TMSCl, trimethylsilyl chloride.

b Isolated yield.

c At 75 °C.

d **2a** (0.2 mmol), **3a** (0.2 mmol).

After determining the optimal reaction conditions, we began to explore the substrate suitability of the reaction (Scheme 2). First, some long chain and cyclic aldehydes (**4b–4f**) were attempted, and all achieved moderate to good yields. During the subsequent exploration, we found that adding a catalytic amount of cuprous iodide would increase the yield of the reaction. Based on the relevant literature, we speculated that cuprous iodide might enhance the reactivity of alkyl iodine. Based on this, we began to test the reactivity of alkyl iodides with different substituents. The results showed that both short chain (**4aa**) and long chain (**4ab–4ad**) alkyl iodides could effectively participate in the reaction, and those with various substituents such as -CF_3_, -Cl, and -I (**4ae–4ag**, respectively) could also afford the corresponding products. All kinds of iodomethyl cycloalkanes (**4ai–4al**) achieved excellent reaction effects. Moreover, iodocyclopentane can also yield the target product (**4h**). Then, we conducted substrate exploration on amines, and the results indicated that both cyclic amines such as cyclohexylamine (**4am–4ao**) and chain-like amines (**4ap–4ar**) could achieve good yields. The aldehyde substrates are concentrated in primary aldehydes. Isopentaldehyde (**4aw**) and 3,3-dimethylbutyraldehyde (**4ax**) performed well, but it is found through attempts that aryl aldehydes are not suitable for this transformation. The addition of CuI can improve the yield, which benefited by strengthening alkyl iodide activation. However, no desired product was detected when primary amines or aryl amines were tested.

**Scheme 2:**
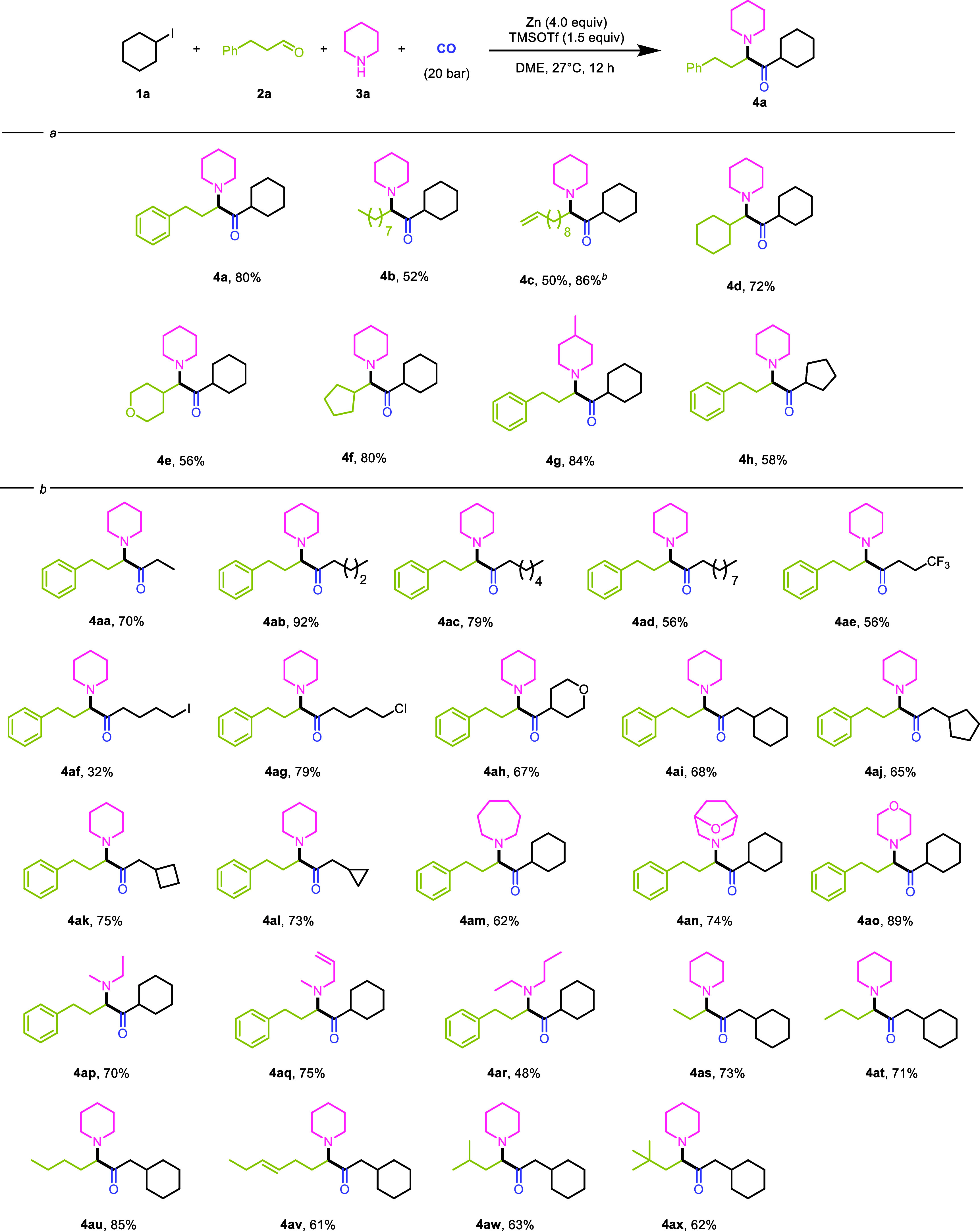
Substrate Testing^a^

a Reaction conditions: **1a** (0.3 mmol), **2a** (0.1 mmol), **3a** (0.15 mmol), Zn (4.0 equiv), TMSOTf (1.5 equiv), DME (1 mL), CO (20 bar), 27 °C.
b With CuI (40 mol %) as an additive.

After testing the substrate suitability of the reaction, we began to study the reaction mechanism of this transformation (Scheme 3). When 4 equiv of TEMPO was added under the standard conditions of the reaction, the reaction was completely suppressed. Similarly, when 1,1-diphenylethylene was added, only a trace of the product was detected, while the products of radical addition and radical capture were detected by GC-MS. We concluded that radical species were generated (Scheme 3, eq 1).^15,16^ Subsequently, we determined through cyclic voltammetry that the reduction potential of the imine cation,^17^ *E*_1/2_((C–H)^+^/(C–H)•), was −1.48 V (see the Supporting Information; Scheme 3, eq 2).^17^ The reduction potential of zinc, *E*(Zn^2+^/Zn), is −0.76 V,^18^ which not enough to reduce either imine or alkyl iodide (−1 V).^19^ Taking all of the information into consideration, a possible reaction pathway is proposed accordingly (Scheme 3, eq 3). Under the action of a Lewis acid, aldehyde and amine reacted to form an imine cation. Meanwhile, alkyl iodide was activated by zinc via a radical intermediate, and an acyl radical was formed after the capture of one molecule of carbon monoxide. Then the acyl intermediate was added to imine to obtain the final product.

**Scheme 3:**
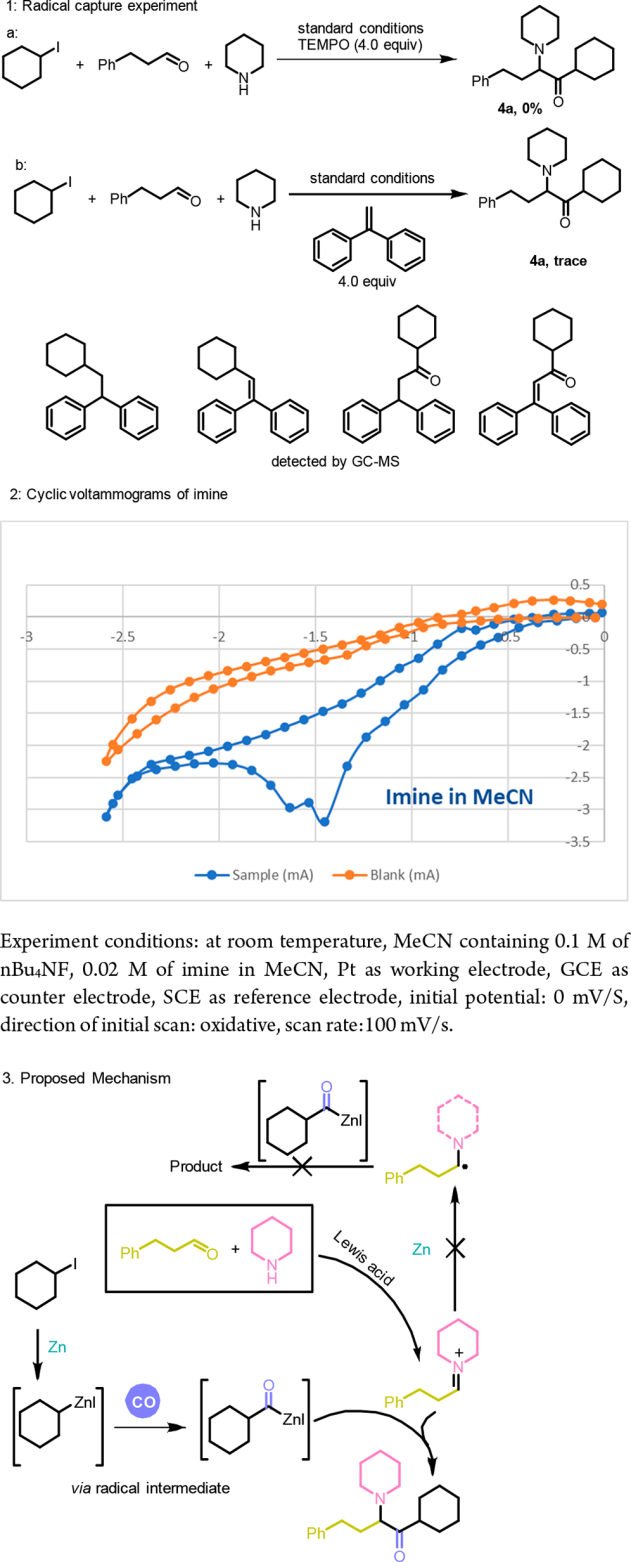
Mechanistic Exploration and Proposal

In summary, we have developed a method for obtaining α-aminoketone through a zinc-mediated four-component carbonylation reaction. The reaction was simple and efficient. Using inexpensive and readily available alkyl iodides, aldehydes, and amines as starting materials, a series of target products were obtained in moderate to good yields, demonstrating the universality and selectivity of this transformation.

## Supplementary Material



## Data Availability

The data underlying this study are available in the published article and its Supporting Information.

## Notes

Experimental details, characterization data, and NMR spectra (PDF)
