# Supplementary material for: Zinc-Mediated Four-Component Carbonylation toward Direct Synthesis of α‑Amino Ketones
Source: Org Lett. 2026 Mar 3;28(10):3366–70. doi: 10.1021/acs.orglett.6c00604 (PMC12993920; doi:10.1021/acs.orglett.6c00604)

Supporting Information

Zinc-Mediated Four-Component Carbonylation Toward Direct Synthesis of α-Amino Ketones

Qiangwei Li[†] and Xiao-Feng Wu*[†]

[†] Dalian National Laboratory for Clean Energy, Dalian Institute of Chemical Physics, Chinese Academy of Sciences, 116023 Dalian, Liaoning, China; Leibniz-Institut für Katalyse e.V., Albert-Einstein-Str. 29a, 18059 Rostock, Germany

E-mail: xwu2020@dicp.ac.cn (X.-F. Wu).

Contents

1. General Information. 2

2. Optimization of the Reaction Conditions. 3

3. Mechanistic investigations. 7

    3.1 Reduction potential of Imine. 7

4. General Experimental Procedure and Characterization Data. 9

5. Copies of ^{1}H, ^{13}C and ^{19}F Spectra. 24

1. General Information.

Unless otherwise noted, all reactions were carried out under carbon monoxide or nitrogen atmosphere. All reagents were from commercial sources (Sigma-Aldrich, Bidepharm, EnergyChemical) and used as received without further purification. All solvents were dried by standard techniques and distilled prior to use. Column chromatography was performed on silica gel (200-300 meshes) using petroleum ether (bp. 60~90 °C), dichloromethane and ethyl acetate as eluent. All NMR spectra were recorded at ambient temperature using Bruker Avance III 400 MHz NMR (^{1}H, 400 MHz; ^{13}C{^{1}H}, 101 MHz, ^{19}F 376 MHz). ^{1}H NMR chemical shifts are reported relative to TMS and were referenced via residual proton resonances of the corresponding deuterated solvent ($CDCl_3$: 7.26 ppm) whereas ^{13}C{^{1}H} NMR spectra are reported relative to TMS via the carbon signals of the deuterated solvent ($CDCl_3$: 77.0 ppm. Data for ^{1}H are reported as follows: chemical shift (δ ppm), multiplicity (s = singlet, d = doublet, t = triplet, q = quartet, dd (doublet of doublets), dt (doublet of triplets), qd (quartet of doublets), quint = quintet, m = multiplet, br = broad), coupling constant (Hz), and integration. All ^{13}C NMR spectra were broad-band ^{1}H decoupled. All reactions were monitored by GC-FID or NMR analysis. HRMS data was obtained with Micromass HPLC-Q-TOF mass spectrometer (ESI-TOF) or Agilent 6540 Accurate-MS spectrometer (Q-TOF). FT-ICR MS was obtained with Solarix FT-ICR MS (15 T, Bruker Daltonics GmbH, Bremen, Germany). Because of the high toxicity of carbon monoxide, all the reactions should be performed in an autoclave. The laboratory should be well-equipped with a CO detector and alarm system.

2. Optimization of the Reaction Conditions.

Table S1. Optimization of solvent.

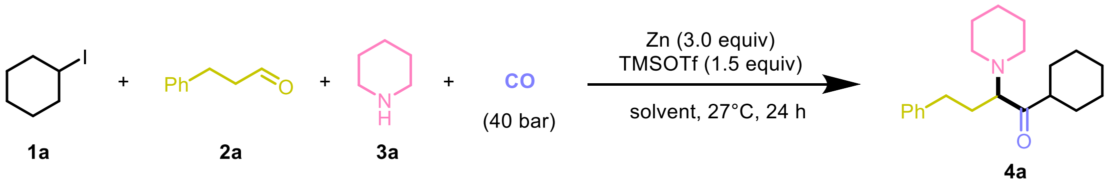

Entry	Solvent	Yield (%)
1	DCM	40
2	THF	18
3	EA	54
4	MeCN	16
5	DMF	5
6	DME	72

Reaction conditions: **1a** (0.3 mmol), **2a** (0.15 mmol), **3a** (0.10 mmol), Zn (3.0 equiv.), TMSOTf (1.5 equiv.), CO (40 bar), solvent (1 mL), 27°C, 24 h. The yields were determined by GC using dodecane as the internal standard.

Table S2. Optimization of zinc usage.

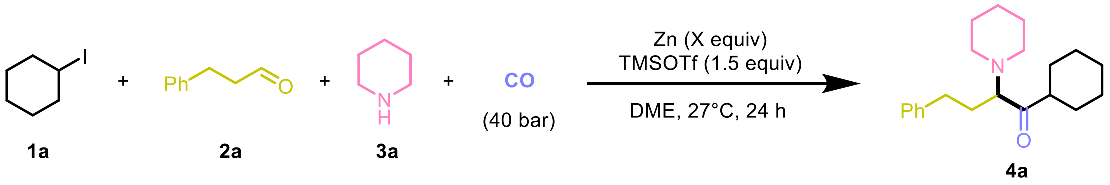

Entry	X	Yield (%)
1	2.0	14
2	3.0	49
3	4.0	60
4	5.0	61

Reaction conditions: **1a** (0.3 mmol), **2a** (0.15 mmol), **3a** (0.10 mmol), TMSOTf (1.5 equiv.), CO (40 bar), DME (1 mL), 27°C, 24 h. The yields were determined by GC using dodecane as the internal standard.

Table S3. Optimization of ratio among **1a**, **2a** and **3a**.

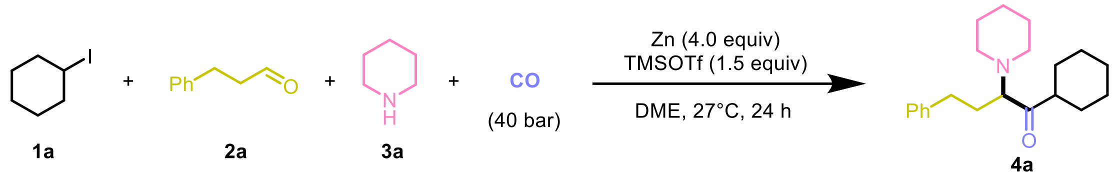

Entry	**1a**:**2a**:**3a** (mmol)	Yield (%)
1	1:2:2	8
2	1:3:3	15
3	2:1:2	25
4	3:1:2	38
5	2:1:1.5	41
6	3:1:1.5	66
7	3:1.5:1	58
8	3:2:1	50

Reaction conditions: TMSOTf (1.5 equiv.), Zn (3.0 equiv.), CO (40 bar), DME (1 mL), 27°C, 24 h. The yields were determined by GC using dodecane as the internal standard.

Table S4. Optimization of solvent usage.

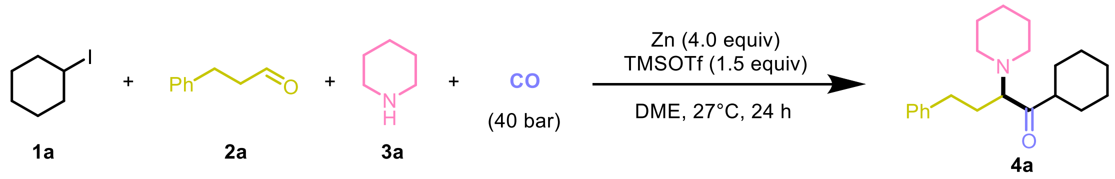

Entry	DME (mL)	Yield (%)
1	0.5	66
2	1.0	66
3	1.5	65
4	2.0	39

Reaction conditions: **1a** (0.3 mmol), **2a** (0.15 mmol), **3a** (0.10 mmol), Zn (4.0 equiv.), TMSOTf (1.5 equiv.), CO (40 bar), 27°C, 24 h. The yields were determined by GC using dodecane as the internal standard.

Table S5. Optimization of pressure.

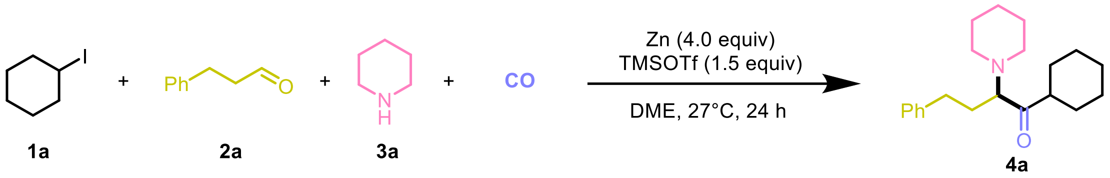

Entry	CO (bar)	Yield (%)
1	60	70
2	30	68
3	20	77
4	10	67
5	5	41

Reaction conditions: **1a** (0.3 mmol), **2a** (0.15 mmol), **3a** (0.10 mmol), Zn (4.0 equiv.), TMSOTf (1.5 equiv.), CO (40 bar), DME (1 mL), 27°C, 24 h. The yields were determined by GC using dodecane as the internal standard.

Table S6. Optimization of reaction time.

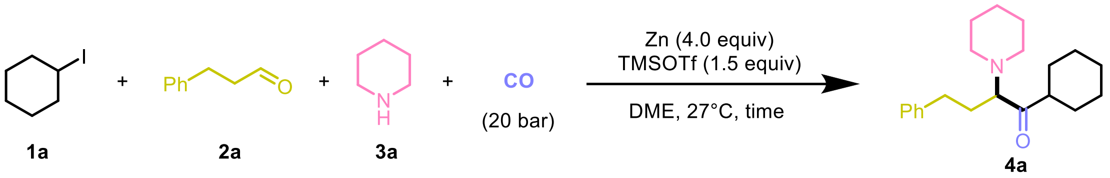

Entry	Time (h)	Yield (%)
1	24	77
2	18	77
3	12	81

Reaction conditions: **1a** (0.3 mmol), **2a** (0.15 mmol), **3a** (0.10 mmol), Zn (4.0 equiv.), TMSOTf (1.5 equiv.), CO (20 bar), DME (1 mL), 27°C. The yields were determined by GC using dodecane as the internal standard.

Table S7. Optimization of Lewis acid.

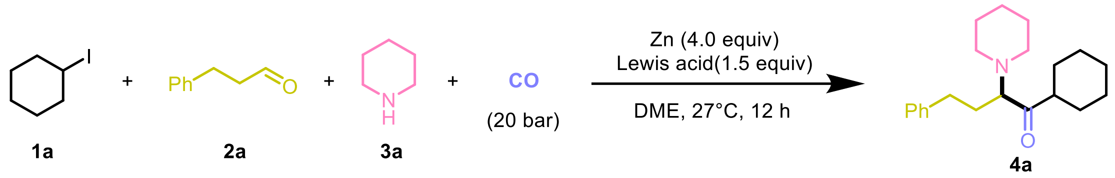

Entry	Lewis acid	Yield (%)
1	TMSOTf	81
2	TBSOTf	22
3	TMSCl	trace
4	TFA	trace

Reaction conditions: **1a** (0.3 mmol), **2a** (0.15 mmol), **3a** (0.10 mmol), Zn (4.0 equiv.), Lewis acid (1.5 equiv.), CO (20 bar), DME (1 mL), 27°C, 12 h. The yields were determined by GC using dodecane as the internal standard.

3. Mechanistic investigations.

3.1 Reduction potential of Imine.

Cyclic Voltammetry studies were performed using an Admiral Squidsta Plus workstation and SUI v2.0 Beat software. Using Pt as working electrode, GCE (Glassy Carbon Electrode) as counter electrode, SCE (Saturated Calomel Electrode) as reference electrode, nBu_4NF as electrolyte. Pt (10 cm^2, rectangular prism). 0.1 M of nBu_4NF, 0.02 M of Imine in MeCN. The experiment was conducted at room temperature. Initial potential: 0 mV/S, direction of initial scan: oxidative, scan rate:100 mV/s. The working electrode is polished with 2000-grit sandpaper, then thoroughly cleaned with deionized water.

Dissolve phenylpropionaldehyde **2a** and piperidine **3a** in 1mL of MeCN, add TMSOTf (1.5 equiv), stir overnight, increase the solvent volume to 5mL, insert the electrode, and measure the reduction potential.

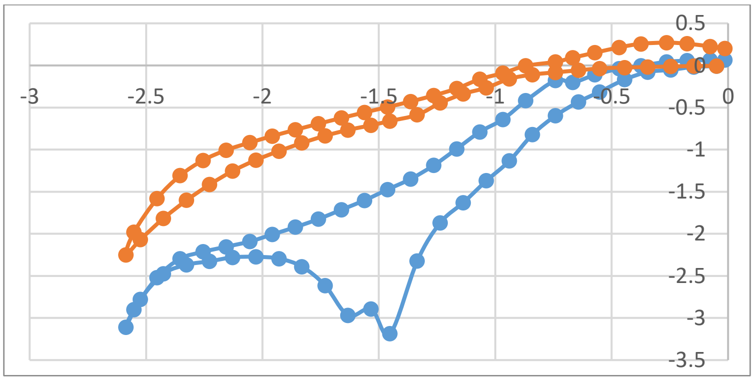

Imine in MeCN

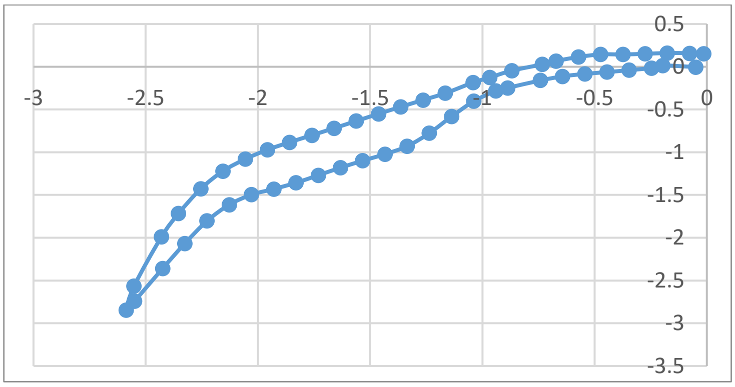

Imine without TMSOTf in MeCN

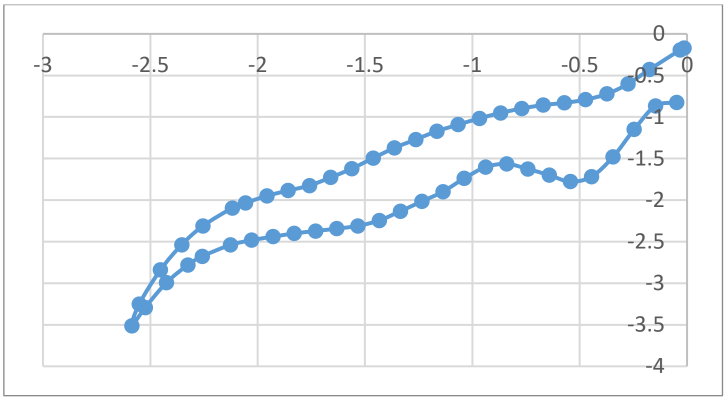

TMSOTf in MeCN

4. General Experimental Procedure and Characterization Data.

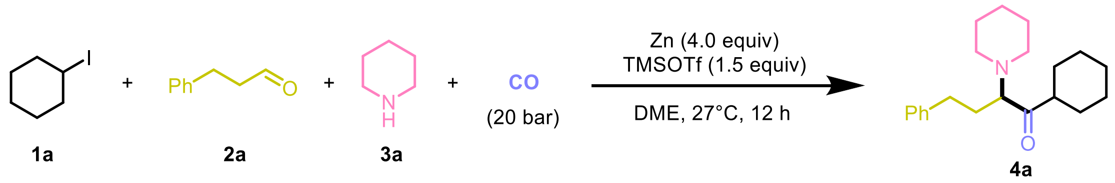

A 4 mL screw-cap vial was charged with Zn (26 mg, 0.4 mmol, 4.0 equiv), DME (1 mL), **3a** piperidine (15 μL, 0.1 mmol, 1.0 equiv), **2a** phenylpropyl aldehyde (14 mg, 1.5 equiv) and **1a** iodocyclohexane (39 μL, 3.0 equiv) was added. After tightening the bottle cap, TMSOTf (27 μL ,1.5 equiv) was added. the vial was moved to an alloy plate and put into an autoclave which are euphotic under argon atmosphere. At room temperature, the autoclave was charged with 20 atm of CO. The autoclave was placed on an environment equipped with a magnetic stirrer. The reaction mixture was stirred for 12 h. After the reaction was complete, the pressure in autoclave was released carefully. The reaction mixture was purified by column chromatography on silica gel using petroleum ether and ethyl acetate (30:1) to afford the product **4a**.

Tips: the products are all colorless liquids.

1 mmol scale: A 12 mL screw-cap vial was charged with Zn (260 mg, 4 mmol, 4.0 equiv), DME (6 mL), **3a** piperidine (150 μL, 1 mmol, 1.0 equiv), **2a** phenylpropyl aldehyde (140 mg, 1.5 equiv) and **1a** iodocyclohexane (390 μL, 3.0 equiv) was added. After tightening the bottle cap, TMSOTf (270 μL ,1.5 equiv) was added. the vial was moved to an alloy plate and put into an autoclave which are euphotic under argon atmosphere. At room temperature, the autoclave was charged with 20 atm of CO. The autoclave was placed on an environment equipped with a magnetic stirrer. The reaction mixture was stirred for 12 h. After the reaction was complete, the pressure in autoclave was released carefully. The reaction mixture was purified by column chromatography on silica gel using petroleum ether and ethyl acetate (30:1) to afford the product **4a** in 71% yield (222.2 mg).

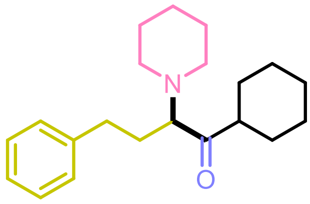

1-cyclohexyl-4-phenyl-2-(piperidin-1-yl)butan-1-one (4a): 25 mg, colorless liquid, 80%. R_f = 0.5 (PE: EA = 10:1). Purified on silica gel using petroleum ether and ethyl acetate.

^{1}H NMR (400 MHz, CDCl$_3$) δ 7.30 – 7.24 (m, 2H), 7.21 – 7.13 (m, 3H), 3.19 (dd, J = 8.8, 4.6 Hz, 1H), 2.73 – 2.54 (m, 2H), 2.54 – 2.34 (m, 5H), 2.05 – 1.90 (m, 1H), 1.86 – 1.70 (m, 6H), 1.58 – 1.49 (m, 4H), 1.45 – 1.35 (m, 3H), 1.31 – 1.19 (m, 4H).

^{13}C NMR (101 MHz, CDCl$_3$) δ 214.0, 142.2, 128.4, 128.3, 125.8, 70.9, 50.9, 48.8, 33.0, 29.2, 28.1, 26.7, 26.0, 25.9, 25.8, 25.6, 24.6.

HRMS(ESI-TOF) m/z: calcd for [M+]H+ $C_{21}H_{32}NO^+$ 314.2478, found: 314.2489.

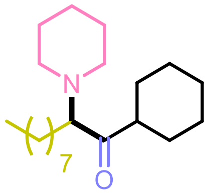

1-cyclohexyl-2-(piperidin-1-yl)decan-1-one (4b): 16.6 mg, colorless liquid, 52%, R_f = 0.5 (PE: EA = 10:1). Purified on silica gel using petroleum ether and ethyl acetate.

^{1}H NMR (400 MHz, CDCl$_3$) δ 3.15 (dd, J = 8.8, 4.4 Hz, 1H), 2.63 (dd, J = 15.1, 7.0 Hz, 1H), 2.55 – 2.35 (m, 4H), 1.83 – 1.63 (m, 6H), 1.51 (s, 4H), 1.46 – 1.35 (m, 3H), 1.26 (d, J = 13.4 Hz, 17H), 0.87 (t, J = 6.7 Hz, 3H).

^{13}C NMR (101 MHz, CDCl$_3$) δ 214.2, 72.0, 51.0, 49.0, 31.9, 29.9, 29.5, 29.3, 29.1, 28.2, 27.0, 26.6, 26.0, 25.9, 25.6, 24.6, 24.5, 22.7, 14.1.

HRMS(ESI-TOF) m/z: calcd for [M+]H+ $C_{21}H_{40}NO^+$ 322.3104, found: 322.3107.

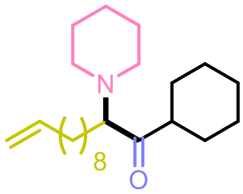

1-cyclohexyl-2-(piperidin-1-yl)dodec-11-en-1-one (4c): 17.2 mg, colorless liquid, 50%, R_f = 0.4 (PE: EA = 10:1). Purified on silica gel using petroleum ether and ethyl acetate.

^{1}H NMR (400 MHz, CDCl$_3$) δ 5.81 (ddt, J = 16.9, 10.1, 6.7 Hz, 1H), 5.08 – 4.75 (m, 2H), 3.14 (dd, J = 8.9, 4.4 Hz, 1H), 2.64 (td, J = 11.1, 3.0 Hz, 1H), 2.56 – 2.33 (m,

4H), 2.03 (q, J = 7.0 Hz, 2H), 1.84 – 1.63 (m, 8H), 1.51 (s, 4H), 1.47 – 1.33 (m, 5H), 1.32 – 1.08 (m, 13H).

^{13}C NMR (101 MHz, CDCl$_3$) δ 214.2, 139.3, 114.1, 72.0, 51.0, 49.0, 33.8, 29.8, 29.4, 29.1, 28.9, 28.2, 27.0, 26.6, 26.0, 25.9, 25.6, 24.6, 24.5.

HRMS(ESI-TOF) m/z: calcd for [M+]H+ $C_{23}H_{42}NO^+$ 348.3261, found: 348.3264.

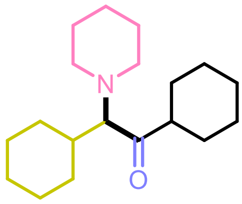

1,2-dicyclohexyl-2-(piperidin-1-yl)ethan-1-one (4d): 21.0 mg, colorless liquid, 72%, R_f = 0.5 (PE: EA = 10:1). Purified on silica gel using petroleum ether and ethyl acetate.

^{1}H NMR (400 MHz, CDCl$_3$) δ 3.00 (d, J = 10.3 Hz, 1H), 2.64 – 2.55 (m, 2H), 2.54 – 2.43 (m, 2H), 2.34 – 2.20 (m, 1H), 1.98 – 1.72 (m, 7H), 1.54 – 1.31 (m, 9H), 1.29 – 1.06 (m, 9H), 0.99 – 0.64 (m, 2H).

^{13}C NMR (101 MHz, CDCl$_3$) δ 212.9, 75.9, 52.2, 51.1, 30.9, 30.4, 28.4, 27.1, 26.8, 26.8, 26.2, 26.1, 26.0, 25.9, 25.6, 24.9.

HRMS(ESI-TOF) m/z: calcd for [M+]H+ $C_{19}H_{34}NO^+$ 292.2635, found: 292.2643.

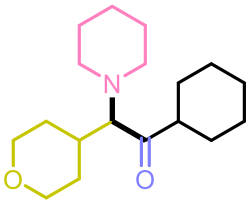

1-cyclohexyl-2-(piperidin-1-yl)-2-(tetrahydro-2H-pyran-4-yl)ethan-1-one (4e): 16.4 mg, colorless liquid, 56%, R_f = 0.4 (PE: EA = 20:1). Purified on silica gel using petroleum ether and ethyl acetate.

^{1}H NMR (400 MHz, CDCl$_3$) δ 3.97 (dd, J = 11.2, 3.6 Hz, 1H), 3.87 (dd, J = 11.2, 3.3 Hz, 1H), 3.36 (dd, J = 25.8, 12.6 Hz, 2H), 3.03 (d, J = 10.4 Hz, 1H), 2.69 – 2.55 (m, 2H), 2.55 – 2.41 (m, 2H), 2.29 (t, J = 11.5 Hz, 1H), 2.15 – 1.97 (m, 1H), 1.92 – 1.73 (m, 5H), 1.67 (d, J = 11.1 Hz, 2H), 1.53 – 1.35 (m, 7H), 1.33 – 1.16 (m, 5H), 1.06 (d, J = 12.1 Hz, 1H).

^{13}C NMR (101 MHz, CDCl$_3$) δ 212.1, 75.4, 68.1, 67.6, 52.2, 51.2, 32.9, 30.8, 30.5, 28.5, 27.1, 26.5, 26.2, 25.9, 25.4, 24.8.

HRMS(ESI-TOF) m/z: calcd for [M+]H+ $C_{18}H_{32}NO_2^+$ 294.2428, found: 294.2435.

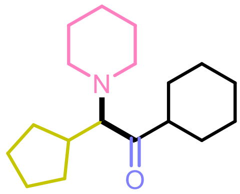

1-cyclohexyl-2-cyclopentyl-2-(piperidin-1-yl)ethan-1-one (4f): 22.1 mg, colorless liquid, 80%, R_f = 0.4 (PE: EA = 20:1). Purified on silica gel using petroleum ether and ethyl acetate.

^{1}H NMR (400 MHz, $CDCl_3$) δ 2.98 (d, J = 10.6 Hz, 1H), 2.59 (s, 4H), 2.41 (t, J = 11.4 Hz, 1H), 2.34 – 2.20 (m, 1H), 1.91 – 1.72 (m, 5H), 1.72 – 1.35 (m, 14H), 1.34 – 1.08 (m, 4H), 0.96 – 0.78 (m, 1H).

^{13}C NMR (100 MHz, $CDCl_3$) δ 213.0, 76.0, 51.2, 50.9, 37.9, 30.7, 30.7, 28.8, 27.5, 27.1, 26.2, 25.9, 25.6, 25.2, 25.0, 24.9.

HRMS(ESI-TOF) m/z: calcd for [M+]H+ $C_{18}H_{32}NO^+$ 278.2478, found: 278.2480.

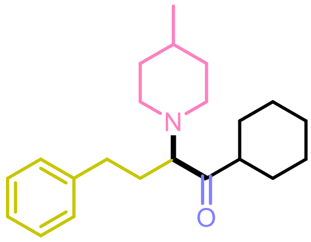

1-cyclohexyl-2-(4-methylpiperidin-1-yl)-4-phenylbutan-1-one (4g): 27.4 mg, colorless liquid, 84%, R_f = 0.4 (PE: EA = 20:1). Purified on silica gel using petroleum ether and ethyl acetate.

^{1}H NMR (400 MHz, $CDCl_3$) δ 7.42 – 7.22 (m, 2H), 7.18 (t, J = 5.9 Hz, 3H), 3.24 (dd, J = 8.8, 4.6 Hz, 1H), 2.73 (d, J = 11.3 Hz, 1H), 2.69 – 2.53 (m, 3H), 2.49 – 2.35 (m, 2H), 2.18 (td, J = 11.3, 2.0 Hz, 1H), 2.08 – 1.89 (m, 1H), 1.84 – 1.64 (m, 6H), 1.57 (t, J = 14.5 Hz, 2H), 1.44 – 1.05 (m, 8H), 0.90 (d, J = 6.3 Hz, 3H).

^{13}C NMR (100 MHz, $CDCl_3$) δ 214.1, 142.1, 128.4, 128.4, 125.8, 70.5, 51.2, 49.1, 48.9, 35.1, 34.8, 33.0, 30.9, 29.2, 28.1, 26.0, 25.9, 25.6, 22.0.

HRMS(ESI-TOF) m/z: calcd for [M+]H+ $C_{22}H_{34}NO^+$ 328.2635, found: 328.2638.

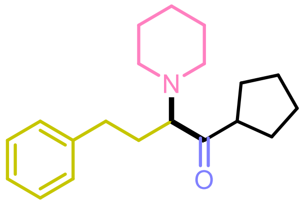

1-cyclopentyl-4-phenyl-2-(piperidin-1-yl)butan-1-one (4h): 17.3 mg, colorless liquid, 58%, R_f = 0.5 (PE: EA = 10:1). Purified on silica gel using petroleum ether and ethyl acetate.

^{1}H NMR (400 MHz, CDCl$_3$) δ 7.26 (d, J = 6.4 Hz, 2H), 7.18 (d, J = 6.5 Hz, 3H), 3.27 – 3.17 (m, 1H), 3.14 (dd, J = 8.8, 4.8 Hz, 1H), 2.68 – 2.53 (m, 1H), 2.55 – 2.35 (m, 5H), 2.06 – 1.90 (m, 1H), 1.90 – 1.64 (m, 6H), 1.64 – 1.48 (m, 7H), 1.46 – 1.35 (m, 2H).

^{13}C NMR (101 MHz, CDCl$_3$) δ 214.4, 142.1, 128.4, 128.4, 125.8, 72.5, 51.0, 49.2, 33.0, 30.5, 29.3, 26.6, 26.2, 26.2, 26.2, 24.6.

HRMS(ESI-TOF) m/z: calcd for [M+]H+ $C_{20}H_{30}NO^+$ 300.2322, found: 300.2321.

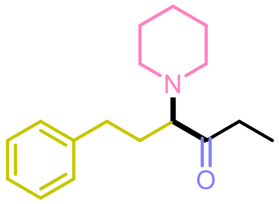

6-phenyl-4-(piperidin-1-yl)hexan-3-one (4aa): 18.2 mg, colorless liquid, 70%, R_f = 0.4 (PE: EA = 10:1). Purified on silica gel using petroleum ether and ethyl acetate.

^{1}H NMR (400 MHz, CDCl$_3$) δ 7.31 – 7.22 (m, 2H), 7.17 (d, J = 7.6 Hz, 3H), 3.05 (dd, J = 8.6, 5.1 Hz, 1H), 2.67 – 2.54 (m, 2H), 2.54 – 2.38 (m, 6H), 2.03 – 1.91 (m, 1H), 1.89 – 1.78 (m, 1H), 1.59 – 1.49 (m, 4H), 1.45 – 1.35 (m, 2H), 1.04 (t, J = 7.3 Hz, 3H).

^{13}C NMR (100 MHz, CDCl$_3$) δ 212.7, 142.0, 128.4, 128.4, 125.9, 72.7, 51.0, 34.5, 33.0, 27.1, 26.6, 24.6, 7.8.

HRMS(ESI-TOF) m/z: calcd for [M+]H+ $C_{17}H_{26}NO^+$ 260.2009, found: 260.2018.

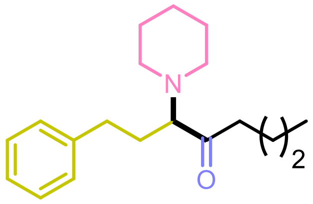

1-phenyl-3-(piperidin-1-yl)octan-4-one (4ab): 26.3 mg, colorless liquid, 92%, R_f = 0.5 (PE: EA = 10:1). Purified on silica gel using petroleum ether and ethyl acetate.

^{1}H NMR (400 MHz, CDCl$_3$) δ 7.32 – 7.23 (m, 2H), 7.17 (d, J = 7.6 Hz, 3H), 3.03 (dd, J = 8.6, 5.0 Hz, 1H), 2.67 – 2.54 (m, 2H), 2.53 – 2.35 (m, 6H), 2.04 – 1.90 (m, 1H), 1.90 – 1.76 (m, 1H), 1.60 – 1.48 (m, 36H), 1.41 (d, J = 5.0 Hz, 2H), 1.37 – 1.23 (m, 2H), 0.91 (t, J = 7.3 Hz, 3H).

^{13}C NMR (100 MHz, CDCl$_3$) δ 212.1, 142.0, 128.4, 128.4, 125.9, 72.8, 51.0, 41.0, 33.0, 26.9, 26.6, 25.9, 24.6, 22.5, 14.0.

HRMS(ESI-TOF) m/z: calcd for [M+]H+ $C_{19}H_{30}NO^+$ 288.2322, found: 288.2325.

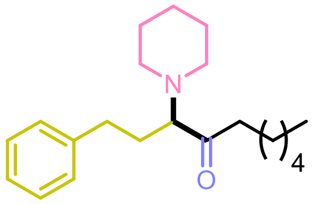

1-phenyl-3-(piperidin-1-yl)decan-4-one (4ac): 25.0 mg, colorless liquid, 79%, R_f = 0.5 (PE: EA = 10:1). Purified on silica gel using petroleum ether and ethyl acetate.

^{1}H NMR (400 MHz, CDCl$_3$) δ 7.33 – 7.23 (m, 2H), 7.18 (t, J = 6.0 Hz, 3H), 3.03 (dd, J = 8.8, 5.0 Hz, 1H), 2.67 – 2.52 (m, 2H), 2.52 – 2.38 (m, 6H), 2.04 – 1.90 (m, 1H), 1.89 – 1.78 (m, 1H), 1.60 – 1.49 (m, 6H), 1.47 – 1.37 (m, 2H), 1.35 – 1.22 (m, 6H), 0.88 (t, J = 6.7 Hz, 3H).

^{13}C NMR (100 MHz, CDCl$_3$) δ 212.1, 142.0, 128.4, 128.4, 125.9, 72.8, 51.0, 41.3, 33.0, 31.7, 29.0, 26.9, 26.6, 24.6, 23.7, 22.5, 14.1.

HRMS(ESI-TOF) m/z: calcd for [M+]H+ $C_{21}H_{34}NO^+$ 316.2635, found: 316.2643.

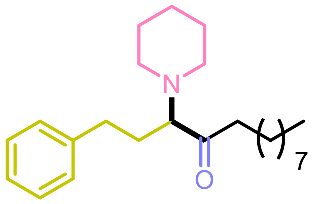

1-phenyl-3-(piperidin-1-yl)tridecan-4-one (4ad): 20.0 mg, colorless liquid, 56%, R_f = 0.5 (PE: EA = 10:1). Purified on silica gel using petroleum ether and ethyl acetate.

^{1}H NMR (400 MHz, CDCl$_3$) δ 7.34 – 7.23 (m, 2H), 7.18 (t, J = 6.1 Hz, 3H), 3.03 (dd, J = 8.7, 5.0 Hz, 1H), 2.66 – 2.52 (m, 2H), 2.52 – 2.38 (m, 6H), 2.02 – 1.90 (m, 1H), 1.89 – 1.78 (m, 1H), 1.60 – 1.48 (m, 6H), 1.46 – 1.37 (m, 2H), 1.27 (s, 12H), 0.88 (t, J = 6.6 Hz, 3H).

^{13}C NMR (100 MHz, CDCl$_3$) δ 212.2, 142.0, 128.4, 128.4, 125.9, 72.8, 51.0, 41.3, 33.0, 31.9, 29.5, 29.3, 29.3, 26.9, 26.6, 24.6, 23.8, 22.7, 14.1.

HRMS(ESI-TOF) m/z: calcd for [M+]H+ $C_{24}H_{40}NO^+$ 358.3104, found: 358.3106.

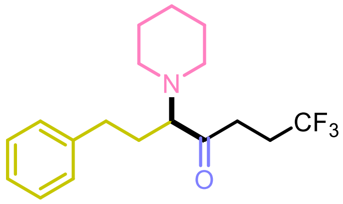

1,1,1-trifluoro-7-phenyl-5-(piperidin-1-yl)heptan-4-one (4ae): 18.4 mg, colorless liquid, 56%, R_f = 0.3 (PE: EA = 10:1). Purified on silica gel using petroleum ether and ethyl acetate.

^{1}H NMR (400 MHz, CDCl$_3$) δ 7.28 (t, J = 7.4 Hz, 2H), 7.23 – 7.09 (m, 3H), 3.04 (dd, J = 8.9, 4.6 Hz, 1H), 3.00 – 2.87 (m, 1H), 2.71 – 2.57 (m, 2H), 2.55 – 2.29 (m, 7H), 2.06 – 1.92 (m, 1H), 1.91 – 1.80 (m, 1H), 1.59 – 1.49 (m, 4H), 1.42 (dd, J = 11.3, 5.6 Hz, 2H).

^{13}C NMR (100 MHz, CDCl$_3$) δ 208.3, 141.7, 128.4, 127.1 (q, JC-F = 275.7 Hz), 72.9, 51.0, 33.3 (q, $J_{C\text{-}F}$ = 2.5 Hz), 33.1, 28.1 (q, $J_{C\text{-}F}$ = 29.7 Hz), 26.6, 26.1, 24.4.

^{19}F NMR (376 MHz, CDCl$_3$) δ -66.5.

HRMS(ESI-TOF) m/z: calcd for [M+]H+ $C_{18}H_{25}F_3NO^+$ 328.1883, found: 328.1888.

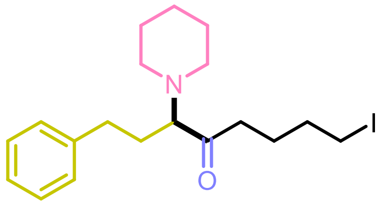

8-iodo-1-phenyl-3-(piperidin-1-yl)octan-4-one (4af): 13.2 mg, colorless liquid, 32%, R_f = 0.5 (PE: EA = 10:1). Purified on silica gel using petroleum ether and ethyl acetate.

^{1}H NMR (400 MHz, CDCl$_3$) δ 7.32 – 7.22 (m, 2H), 7.18 (t, J = 6.1 Hz, 3H), 3.03 (dd, J = 8.7, 5.0 Hz, 1H), 2.67 – 2.54 (m, 2H), 2.53 – 2.37 (m, 5H), 2.02 – 1.89 (m, 1H), 1.90 – 1.76 (m, 1H), 1.60 – 1.47 (m, 6H), 1.46 – 1.37 (m, 2H), 1.37 – 1.25 (m, 2H), 0.91 (t, J = 7.3 Hz, 3H).

^{13}C NMR (100 MHz, CDCl$_3$) δ 212.1, 142.0, 128.4, 128.4, 125.9, 72.8, 51.0, 41.0, 33.0, 26.9, 26.6, 25.9, 24.6, 22.5, 14.0.

HRMS(ESI-TOF) m/z: calcd for [M+]H+ $C_{19}H_{29}INO^+$ 414.1288, found: 414.1291.

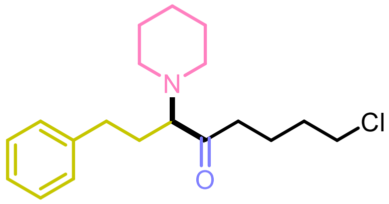

8-chloro-1-phenyl-3-(piperidin-1-yl)octan-4-one (4ag): 25.4 mg, colorless liquid, 79%, R_f = 0.3 (PE: EA = 10:1). Purified on silica gel using petroleum ether and ethyl acetate.

^{1}H NMR (400 MHz, $CDCl_3$) δ 7.34 – 7.23 (m, 2H), 7.18 (t, J = 7.5 Hz, 3H), 3.54 (t, J = 6.2 Hz, 2H), 3.02 (dd, J = 8.8, 4.8 Hz, 1H), 2.70 – 2.55 (m, 2H), 2.53 – 2.27 (m, 6H), 2.03 – 1.90 (m, 1H), 1.89 – 1.63 (m, 5H), 1.53 (d, J = 5.1 Hz, 4H), 1.47 – 1.34 (m, 2H).

^{13}C NMR (100 MHz, $CDCl_3$) δ 211.1, 141.9, 128.4, 128.4, 125.9, 72.8, 51.0, 44.8, 40.1, 33.0, 32.1, 26.6, 26.5, 24.5, 21.1.

HRMS(ESI-TOF) m/z: calcd for [M+]H+ $C_{19}H_{29}ClNO^{+}$ 322.1932, found: 322.1935.

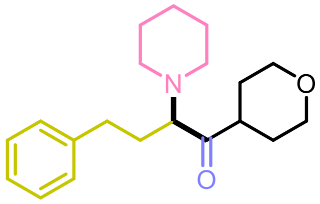

4-phenyl-2-(piperidin-1-yl)-1-(tetrahydro-2H-pyran-4-yl)butan-1-one (4ah): 21.4 mg, colorless liquid, 67%, R_f = 0.3 (PE: EA = 5:1). Purified on silica gel using petroleum ether and ethyl acetate.

^{1}H NMR (400 MHz, $CDCl_3$) δ 7.32 – 7.24 (m, 2H), 7.18 (t, J = 7.9 Hz, 3H), 4.09 – 3.90 (m, 2H), 3.50 – 3.33 (m, 2H), 3.18 (dd, J = 9.2, 4.1 Hz, 1H), 2.91 (tt, J = 11.3, 3.9 Hz, 1H), 2.69 – 2.56 (m, 1H), 2.54 – 2.33 (m, 5H), 2.10 – 1.93 (m, 1H), 1.87 – 1.73 (m, 2H), 1.73 – 1.57 (m, 3H), 1.57 – 1.46 (m, 4H), 1.41 (dd, J = 11.2, 5.5 Hz, 2H).

^{13}C NMR (100 MHz, $CDCl_3$) δ 211.9, 141.9, 128.4, 125.9, 70.6, 67.5, 67.3, 50.8, 45.4, 33.1, 29.0, 28.0, 26.6, 24.9, 24.4.

HRMS(ESI-TOF) m/z: calcd for [M+]H+ $C_{20}H_{30}NO_2^{+}$ 316.2271, found: 316.2278.

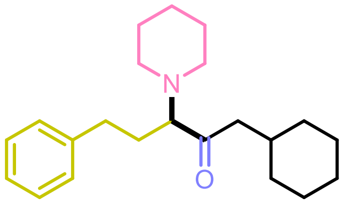

1-cyclohexyl-5-phenyl-3-(piperidin-1-yl)pentan-2-one (4ai): 22.3 mg, colorless liquid, 68%, R_f = 0.5 (PE: EA = 10:1). Purified on silica gel using petroleum ether and ethyl acetate.

^{1}H NMR (400 MHz, CDCl$_3$) δ 7.25 – 7.15 (m, 2H), 7.10 (d, J = 7.7 Hz, 3H), 2.92 (dd, J = 8.7, 4.9 Hz, 1H), 2.63 – 2.46 (m, 1H), 2.46 – 2.30 (m, 6H), 1.95 – 1.81 (m, 1H), 1.83 – 1.67 (m, 2H), 1.60 (d, J = 9.7 Hz, 6H), 1.52 – 1.41 (m, 4H), 1.39 – 1.29 (m, 2H), 1.28 – 1.13 (m, 2H), 1.14 – 0.98 (m, 1H), 0.93 – 0.76 (m, 2H).

^{13}C NMR (100 MHz, CDCl$_3$) δ 211.3, 142.1, 128.4, 128.4, 125.9, 72.9, 51.0, 49.0, 33.5, 33.4, 33.3, 33.0, 26.6, 26.5, 26.3, 26.2, 26.2, 24.6.

HRMS(ESI-TOF) m/z: calcd for [M+]H+ $C_{22}H_{34}NO^+$ 328.2635, found: 328.2646.

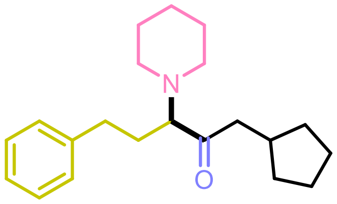

1-cyclopentyl-5-phenyl-3-(piperidin-1-yl)pentan-2-one (4aj): 20.2 mg, colorless liquid, 65%, R_f = 0.5 (PE: EA = 10:1). Purified on silica gel using petroleum ether and ethyl acetate.

^{1}H NMR (400 MHz, CDCl$_3$) δ 7.31 – 7.23 (m, 2H), 7.17 (d, J = 7.8 Hz, 3H), 3.02 (dd, J = 8.8, 4.9 Hz, 1H), 2.68 – 2.38 (m, 8H), 2.33 – 2.18 (m, 1H), 2.03 – 1.89 (m, 1H), 1.89 – 1.74 (m, 3H), 1.67 – 1.47 (m, 8H), 1.46 – 1.35 (m, 2H), 1.16 – 1.00 (m, 2H).

^{13}C NMR (100 MHz, CDCl$_3$) δ 211.7, 142.1, 128.4, 128.4, 125.9, 72.8, 51.0, 47.6, 35.4, 33.0, 32.8, 32.6, 26.6, 25.0, 25.0, 24.6.

HRMS(ESI-TOF) m/z: calcd for [M+]H+ $C_{21}H_{32}NO^+$ 314.2478, found: 314.2485.

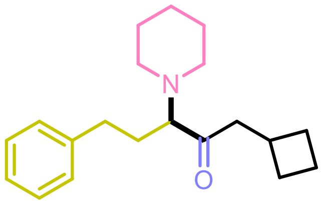

1-cyclobutyl-5-phenyl-3-(piperidin-1-yl)pentan-2-one (4ak): 22.3 mg, colorless liquid, 75%, R_f = 0.5 (PE: EA = 10:1). Purified on silica gel using petroleum ether and

ethyl acetate.

^{1}H NMR (400 MHz, CDCl$_3$) δ 7.30 – 7.24 (m, 2H), 7.18 (t, J = 7.1 Hz, 3H), 2.98 (dd, J = 8.7, 4.8 Hz, 1H), 2.78 – 2.52 (m, 4H), 2.52 – 2.35 (m, 5H), 2.11 (d, J = 1.7 Hz, 2H), 2.01 – 1.77 (m, 4H), 1.72 – 1.57 (m, 2H), 1.58 – 1.47 (m, 4H), 1.47 – 1.36 (m, 2H).

^{13}C NMR (100 MHz, CDCl$_3$) δ 211.3, 142.0, 128.4, 128.4, 125.9, 72.7, 51.0, 48.4, 33.0, 31.6, 28.7, 28.7, 26.6, 24.5, 18.9.

HRMS(ESI-TOF) m/z: calcd for [M+]H+ $C_{20}H_{30}NO^+$ 300.2322, found: 300.2323.

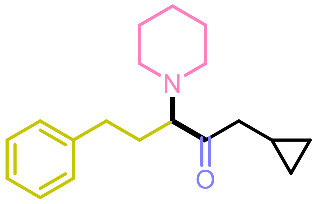

1-cyclopropyl-5-phenyl-3-(piperidin-1-yl)pentan-2-one (4al): 20.7 mg, colorless liquid, 73%, R_f = 0.4 (PE: EA = 10:1). Purified on silica gel using petroleum ether and ethyl acetate.

^{1}H NMR (400 MHz, CDCl$_3$) δ 7.31 – 7.22 (m, 2H), 7.18 (t, J = 6.5 Hz, 3H), 5.97 – 5.70 (m, 1H), 5.01 (dd, J = 28.3, 13.6 Hz, 2H), 3.03 (dd, J = 8.7, 4.9 Hz, 1H), 2.75 – 2.52 (m, 3H), 2.53 – 2.39 (m, 5H), 2.32 (q, J = 7.1 Hz, 2H), 2.03 – 1.91 (m, 1H), 1.89 – 1.77 (m, 1H), 1.53 (dt, J = 10.9, 5.2 Hz, 4H), 1.45 – 1.35 (m, 2H).

^{13}C NMR (100 MHz, CDCl$_3$) δ 211.1, 142.0, 137.6, 128.4, 128.4, 125.9, 115.1, 72.9, 51.0, 40.3, 33.0, 27.8, 26.7, 26.6, 24.5.

HRMS(ESI-TOF) m/z: calcd for [M+]H+ $C_{19}H_{28}NO^+$ 286.2165, found: 286.2169.

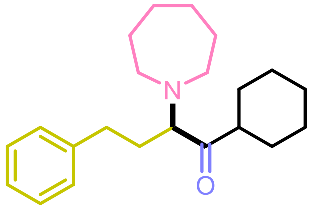

2-(azepan-1-yl)-1-cyclohexyl-4-phenylbutan-1-one (4am): 20.2 mg, colorless liquid, 62%, R_f = 0.5 (PE: EA = 20:1). Purified on silica gel using petroleum ether and ethyl acetate.

^{1}H NMR (400 MHz, CDCl$_3$) δ 7.33 – 7.23 (m, 2H), 7.23 – 7.10 (m, 3H), 3.33 – 3.18 (m, 1H), 2.74 – 2.57 (m, 6H), 2.55 – 2.40 (m, 1H), 2.02 (dt, J = 14.7, 8.6 Hz, 1H), 1.82 – 1.68 (m, 5H), 1.56 (s, 8H), 1.49 – 1.35 (m, 1H), 1.32 – 1.15 (m, 5H).

^{13}C NMR (100 MHz, CDCl$_3$) δ 214.2, 142.2, 128.5, 128.4, 125.8, 70.9, 52.2, 48.3,

33.1, 30.1, 29.4, 28.0, 27.1, 26.8, 26.1, 26.0, 25.6.

HRMS(ESI-TOF) m/z: calcd for [M+]H+ $C_{22}H_{34}NO^+$ 328.2635, found: 328.2641.

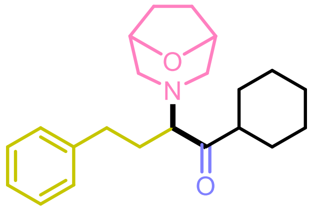

2-(8-oxa-3-azabicyclo[3.2.1]octan-3-yl)-1-cyclohexyl-4-phenylbutan-1-one (4an): 25.2 mg, colorless liquid, 74%, R_f = 0.3 (PE: EA = 10:1). Purified on silica gel using petroleum ether and ethyl acetate.

^{1}H NMR (400 MHz, CDCl$_3$) δ 7.37 – 7.23 (m, 2H), 7.23 – 7.10 (m, 3H), 4.26 (d, J = 18.3 Hz, 2H), 3.13 (dd, J = 8.6, 4.8 Hz, 1H), 2.87 (d, J = 10.8 Hz, 1H), 2.71 – 2.52 (m, 3H), 2.52 – 2.33 (m, 2H), 2.23 (d, J = 10.9 Hz, 1H), 2.06 – 1.58 (m, 11H), 1.52 – 1.35 (m, 1H), 1.34 – 1.13 (m, 4H).

^{13}C NMR (100 MHz, CDCl$_3$) δ 212.6, 141.7, 128.4, 128.4, 126.0, 74.9, 74.8, 68.7, 56.0, 53.4, 49.0, 32.8, 29.2, 28.5, 28.4, 27.9, 26.1, 25.9, 25.6, 25.4.

HRMS(ESI-TOF) m/z: calcd for [M+]H+ $C_{22}H_{32}NO_2^+$ 342.2428, found: 342.2434.

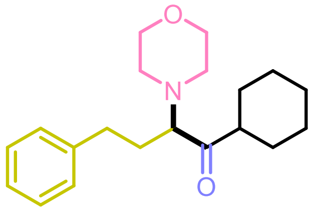

1-cyclohexyl-2-morpholino-4-phenylbutan-1-one (4ao): 28.2 mg, colorless liquid, 89%, R_f = 0.5 (PE: EA = 5:1). Purified on silica gel using petroleum ether and ethyl acetate.

^{1}H NMR (400 MHz, CDCl$_3$) δ 7.28 (t, J = 7.6 Hz, 2H), 7.23 – 7.13 (m, 3H), 3.76 – 3.58 (m, 4H), 3.23 (dd, J = 8.6, 4.8 Hz, 1H), 2.71 – 2.36 (m, 7H), 2.12 – 1.93 (m, 1H), 1.92 – 1.58 (m, 6H), 1.49 – 1.33 (m, 1H), 1.33 – 1.14 (m, 4H).

^{13}C NMR (100 MHz, CDCl$_3$) δ 213.0, 141.7, 128.4, 128.4, 126.0, 70.3, 67.5, 50.0, 49.1, 32.6, 28.9, 28.2, 25.9, 25.9, 25.9, 25.5.

HRMS(ESI-TOF) m/z: calcd for [M+]H+ $C_{20}H_{30}NO_2^+$ 316.2271, found: 316.2278.

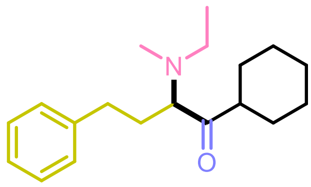

1-cyclohexyl-2-(ethyl(methyl)amino)-4-phenylbutan-1-one (4ap): 20.0 mg, colorless liquid, 70%, R_f = 0.5 (PE: EA = 10:1). Purified on silica gel using petroleum ether and ethyl acetate.

^{1}H NMR (400 MHz, CDCl$_3$) δ 7.33 – 7.24 (m, 2H), 7.18 (t, J = 6.1 Hz, 3H), 3.30 (dd, J = 9.0, 4.2 Hz, 1H), 2.71 (t, J = 11.0 Hz, 1H), 2.65 – 2.55 (m, 1H), 2.53 – 2.35 (m, 3H), 2.20 (s, 3H), 2.09 – 1.94 (m, 1H), 1.83 – 1.64 (m, 6H), 1.50 – 1.36 (m, 1H), 1.34 – 1.16 (m, 4H), 1.00 (t, J = 7.1 Hz, 3H).

^{13}C NMR (100 MHz, CDCl$_3$) δ 214.1, 142.1, 128.4, 128.4, 125.9, 68.4, 48.3, 48.3, 37.8, 33.0, 29.3, 28.1, 26.1, 25.9, 25.5, 24.9, 13.7.

HRMS(ESI-TOF) m/z: calcd for [M+]H+ $C_{19}H_{30}NO^+$ 288.2322, found: 288.2326.

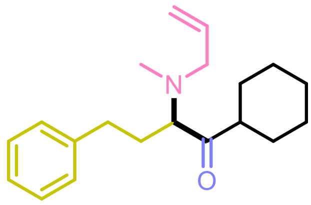

2-(allyl(methyl)amino)-1-cyclohexyl-4-phenylbutan-1-one (4aq): 22.5 mg, colorless liquid, 75%, R_f = 0.4 (PE: EA = 20:1). Purified on silica gel using petroleum ether and ethyl acetate.

^{1}H NMR (400 MHz, CDCl$_3$) δ 7.36 – 7.23 (m, 2H), 7.18 (t, J = 7.0 Hz, 3H), 5.90 – 5.64 (m, 1H), 5.19 – 4.99 (m, 2H), 3.34 (dd, J = 8.8, 4.5 Hz, 1H), 3.15 – 2.99 (m, 2H), 2.75 – 2.53 (m, 2H), 2.50 – 2.36 (m, 1H), 2.20 (s, 3H), 2.10 – 1.95 (m, 1H), 1.85 – 1.59 (m, 6H), 1.51 – 1.35 (m, 1H), 1.34 – 1.13 (m, 4H).

^{13}C NMR (100 MHz, CDCl$_3$) δ 214.0, 142.0, 136.4, 128.5, 128.4, 125.9, 117.1, 67.8, 57.5, 48.3, 38.1, 32.9, 29.2, 28.1, 26.0, 25.9, 25.5, 25.1.

HRMS(ESI-TOF) m/z: calcd for [M+]H+ $C_{20}H_{30}NO^+$ 300.2322, found: 300.2325.

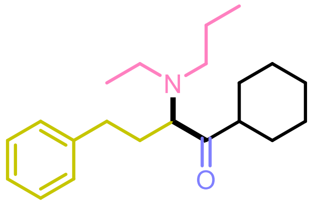

1-cyclohexyl-2-(ethyl(propyl)amino)-4-phenylbutan-1-one (4ar): 15.0 mg, colorless liquid, 48%, R_f = 0.5 (PE: EA = 20:1). Purified on silica gel using petroleum ether and ethyl acetate.

^{1}H NMR (400 MHz, CDCl$_3$) δ 7.32 – 7.21 (m, 2H), 7.18 (t, J = 6.2 Hz, 3H), 3.33 (dd, J = 9.3, 3.7 Hz, 1H), 2.83 (ddd, J = 11.4, 8.4, 3.0 Hz, 1H), 2.70 – 2.57 (m, 1H), 2.55 – 2.45 (m, 1H), 2.43 – 2.29 (m, 4H), 2.12 – 1.97 (m, 1H), 1.85 – 1.72 (m, 3H), 1.70 – 1.59 (m, 4H), 1.54 – 1.41 (m, 1H), 1.39 – 1.28 (m, 2H), 1.29 – 1.15 (m, 3H), 0.94 (t, J = 7.1 Hz, 3H), 0.83 (t, J = 7.3 Hz, 3H).

^{13}C NMR (100 MHz, CDCl$_3$) δ 214.3, 142.2, 128.4, 128.3, 125.8, 65.5, 52.6, 47.5, 44.8, 33.3, 29.7, 28.0, 26.2, 26.0, 25.5, 24.7, 22.0, 14.4, 11.7.

HRMS(ESI-TOF) m/z: calcd for [M+]H+ $C_{21}H_{34}NO^+$ 316.2635, found: 316.2642.

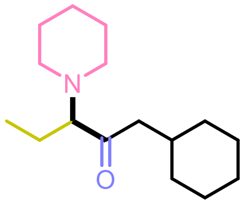

1-cyclohexyl-3-(piperidin-1-yl)pentan-2-one (4as): 18.2 mg, colorless liquid, 73%, R_f = 0.5 (PE: EA = 5:1). Purified on silica gel using petroleum ether and ethyl acetate.

^{1}H NMR (400 MHz, CDCl$_3$) δ 3.08 (dd, J = 9.0, 4.5 Hz, 1H), 2.64 (t, J = 11.1 Hz, 1H), 2.57 – 2.31 (m, 4H), 1.88 – 1.59 (m, 7H), 1.50 (t, J = 9.0 Hz, 5H), 1.45 – 1.36 (m, 3H), 1.35 – 1.16 (m, 5H), 0.81 (t, J = 7.4 Hz, 3H).

^{13}C NMR (100 MHz, CDCl$_3$) δ 214.0, 100.0, 73.6, 51.1, 49.1, 29.0, 28.1, 26.6, 26.0, 25.9, 25.6, 24.6, 17.6, 11.5.

HRMS(ESI-TOF) m/z: calcd for [M+]H+ $C_{16}H_{30}NO^+$ 252.2322, found: 252.2329.

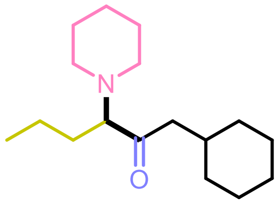

1-cyclohexyl-3-(piperidin-1-yl)hexan-2-one (4at): 18.7 mg, colorless liquid, 71%, R_f = 0.5 (PE: EA = 10:1). Purified on silica gel using petroleum ether and ethyl

acetate.

^{1}H NMR (400 MHz, CDCl$_3$) δ 3.18 (dd, J = 8.7, 4.4 Hz, 1H), 2.73 – 2.58 (m, 1H), 2.56 – 2.35 (m, 4H), 1.83 – 1.73 (m, 3H), 1.73 – 1.58 (m, 3H), 1.57 – 1.48 (m, 4H), 1.47 – 1.35 (m, 4H), 1.32 – 1.10 (m, 8H), 0.89 (t, J = 7.3 Hz, 3H).

^{13}C NMR (100 MHz, CDCl$_3$) δ 214.2, 71.6, 51.0, 49.1, 29.1, 28.1, 26.8, 26.6, 26.0, 25.9, 25.6, 24.55, 20.3, 14.3.

HRMS(ESI-TOF) m/z: calcd for [M+]H+ $C_{17}H_{32}NO^+$ 266.2478, found: 266.2472.

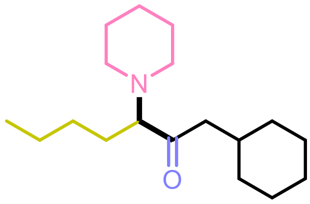

1-cyclohexyl-3-(piperidin-1-yl)heptan-2-one (4au): 23.8 mg, colorless liquid, 85%, R_f = 0.5 (PE: EA = 10:1). Purified on silica gel using petroleum ether and ethyl acetate.

^{1}H NMR (400 MHz, CDCl$_3$) δ 3.15 (dd, J = 9.0, 4.4 Hz, 1H), 2.72 – 2.58 (m, 1H), 2.56 – 2.36 (m, 4H), 1.85 – 1.58 (m, 7H), 1.57 – 1.48 (m, 4H), 1.48 – 1.36 (m, 4H), 1.36 – 1.20 (m, 7H), 1.20 – 1.04 (m, 2H), 0.88 (t, J = 7.3 Hz, 3H).

^{13}C NMR (100 MHz, CDCl$_3$) δ 214.1, 72.0, 51.0, 49.0, 29.3, 29.1, 28.1, 26.6, 26.0, 26.0, 25.6, 24.6, 24.1, 22.9, 14.0.

HRMS(ESI-TOF) m/z: calcd for [M+]H+ $C_{18}H_{34}NO^+$ 280.2635, found: 280.2639.

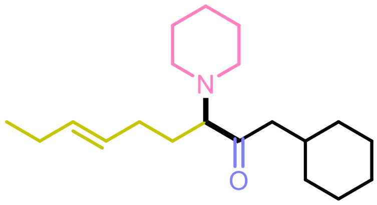

1-cyclohexyl-3-(piperidin-1-yl)non-5-en-2-one (4av): 18.9 mg, colorless liquid, 61%, R_f = 0.5 (PE: EA = 10:1). Purified on silica gel using petroleum ether and ethyl acetate.

^{1}H NMR (400 MHz, CDCl$_3$) δ 5.51 – 5.18 (m, 2H), 3.19 (d, J = 6.8 Hz, 1H), 2.65 (t, J = 10.9 Hz, 1H), 2.56 – 2.36 (m, 4H), 2.07 – 1.90 (m, 3H), 1.87 – 1.62 (m, 8H), 1.51 (d, J = 3.9 Hz, 5H), 1.45 – 1.33 (m, 3H), 1.33 – 1.14 (m, 5H), 0.94 (dd, J = 12.3, 7.4 Hz, 3H).

^{13}C NMR (100 MHz, CDCl$_3$) δ 213.9, 132.6, 128.6, 70.9, 50.9, 48.9, 29.8, 29.2, 28.1, 26.7, 26.0, 26.0, 25.6, 24.6, 24.4, 24.1, 23.8, 20.6, 14.4, 14.0.

HRMS(ESI-TOF) m/z: calcd for [M+]H+ $C_{20}H_{36}NO^+$ 306.2791, found: 306.2783.

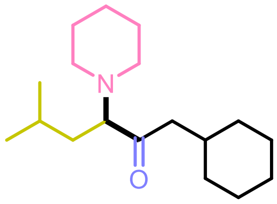

1-cyclohexyl-5-methyl-3-(piperidin-1-yl)hexan-2-one (4aw): 17.5 mg, colorless liquid, 63%, R_f = 0.5 (PE: EA = 10:1). Purified on silica gel using petroleum ether and ethyl acetate.

^{1}H NMR (400 MHz, CDCl$_3$) δ 3.24 (dd, J = 8.7, 4.8 Hz, 1H), 2.68 (ddd, J = 11.2, 7.2, 3.1 Hz, 1H), 2.56 – 2.32 (m, 4H), 1.76 (d, J = 8.9 Hz, 4H), 1.72 – 1.61 (m, 2H), 1.61 – 1.46 (m, 5H), 1.46 – 1.33 (m, 4H), 1.33 – 1.16 (m, 6H), 0.86 (dd, J = 11.4, 6.5 Hz, 6H).

^{13}C NMR (100 MHz, CDCl$_3$) δ 214.2, 69.6, 50.8, 48.8, 32.6, 29.3, 28.2, 26.7, 26.1, 26.0, 25.6, 25.5, 24.6, 23.2, 22.4.

HRMS(ESI-TOF) m/z: calcd for [M+]H+ $C_{18}H_{34}NO^+$ 280.2635, found: 280.2638.

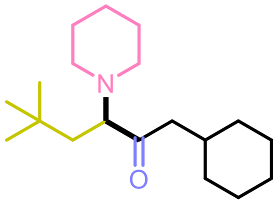

1-cyclohexyl-5,5-dimethyl-3-(piperidin-1-yl)hexan-2-one (4ax): 18.3 mg, colorless liquid, 62%, R_f = 0.5 (PE: EA = 20:1). Purified on silica gel using petroleum ether and ethyl acetate.

^{1}H NMR (400 MHz, CDCl$_3$) δ 3.25 (d, J = 8.4 Hz, 1H), 2.89 – 2.62 (m, 1H), 2.36 (t, J = 5.1 Hz, 4H), 1.87 – 1.71 (m, 5H), 1.67 (d, J = 10.8 Hz, 1H), 1.59 – 1.42 (m, 6H), 1.41 – 1.34 (m, 2H), 1.33 – 1.18 (m, 6H), 0.81 (s, 9H).

^{13}C NMR (100 MHz, CDCl$_3$) δ 212.4, 68.0, 50.4, 48.8, 33.1, 30.0, 29.7, 29.6, 28.7, 26.6, 26.2, 26.0, 25.63, 24.5.

HRMS(ESI-TOF) m/z: calcd for [M+]H+ $C_{19}H_{36}NO^+$ 294.2791, found: 294.2799.

5. Copies of ^{1}H, ^{13}C and ^{19}F Spectra.

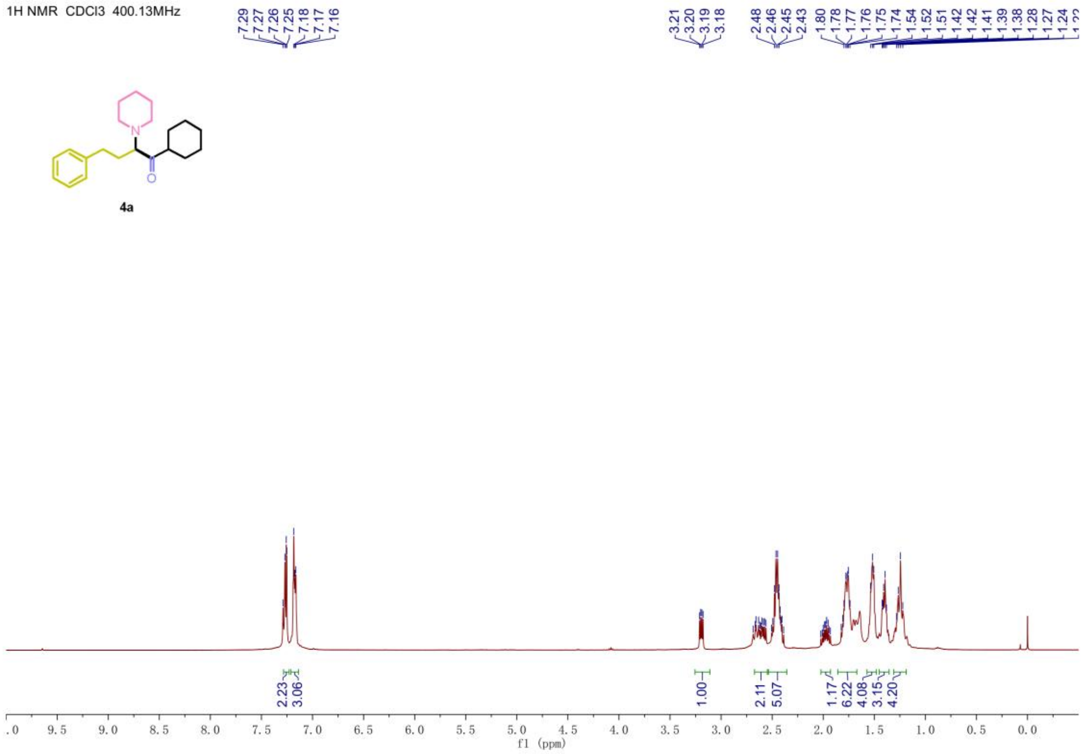

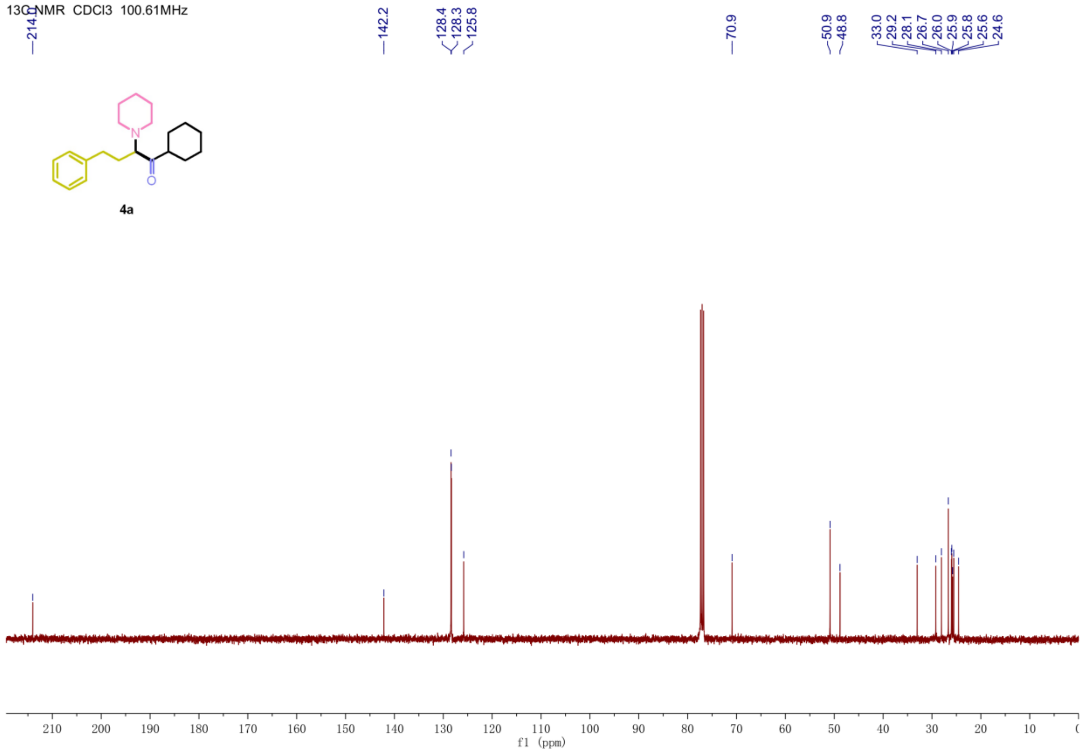

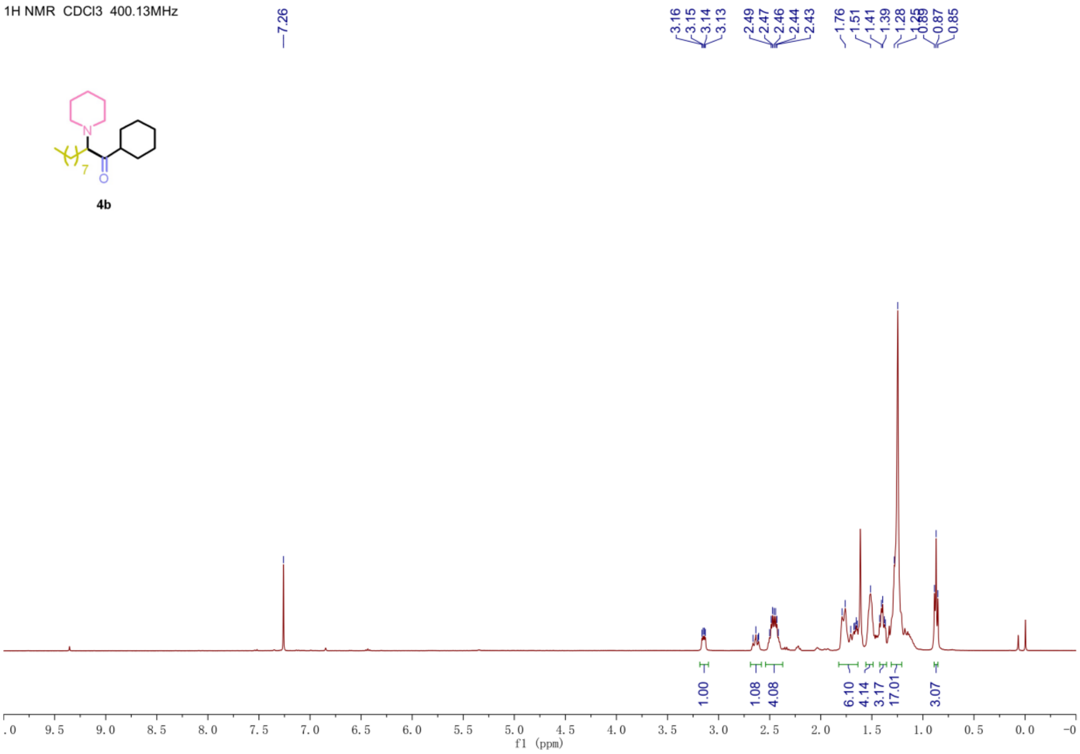
1H NMR CDCl3 400.13MHz
7.26
3.16
3.15
3.14
3.13
2.49
2.47
2.46
2.44
2.43
1.76
1.51
1.41
1.39
1.28
0.87
0.85
4b
1.00
1.08
4.08
6.10
4.14
3.17
17.01
3.07
f1 (ppm)

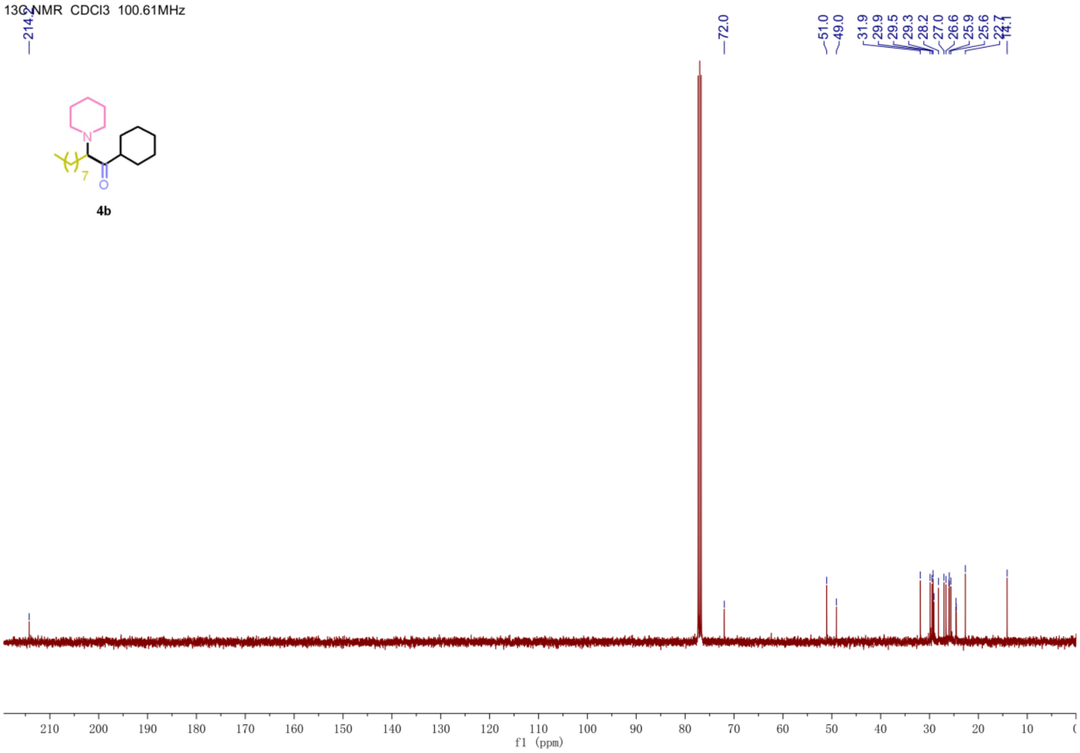
13C NMR CDCl3 100.61MHz
72.0
51.0
49.0
31.9
29.9
29.5
29.3
28.2
27.0
26.6
25.9
25.6
22.7
14.1
4b
f1 (ppm)

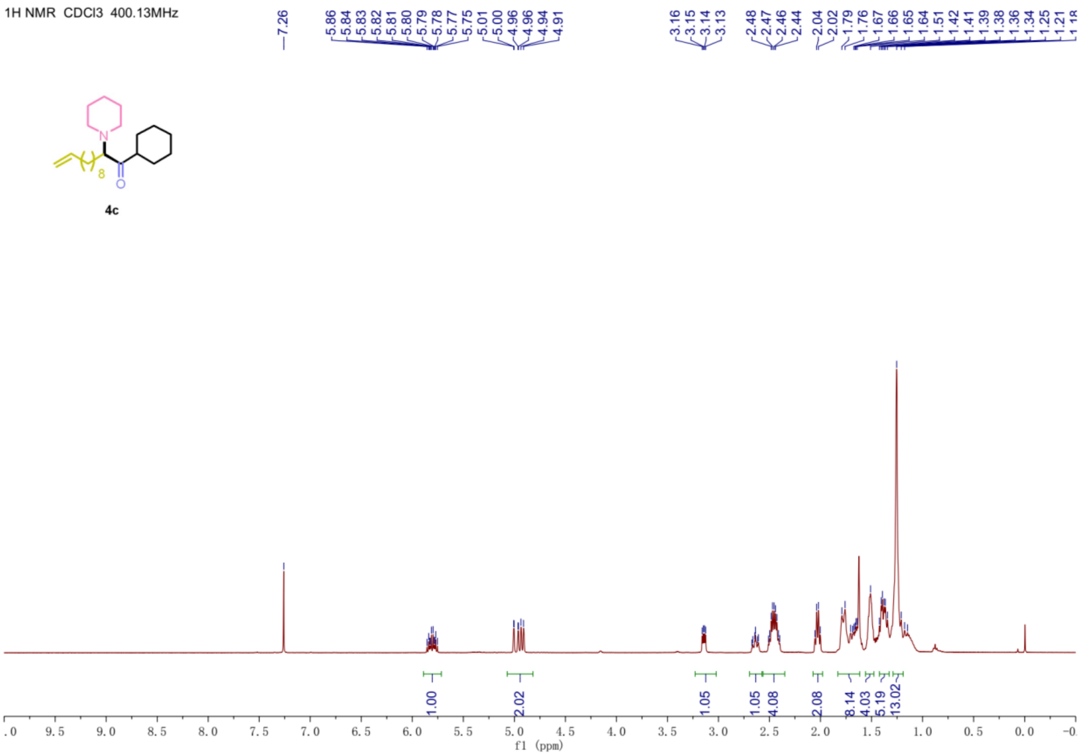
1H NMR CDCl3 400.13MHz
7.26
5.86
5.84
5.83
5.82
5.81
5.80
5.79
5.78
5.77
5.75
5.01
5.00
4.96
4.96
4.94
4.91
3.16
3.15
3.14
3.13
2.48
2.47
2.46
2.44
2.04
2.02
1.79
1.76
1.67
1.66
1.65
1.64
1.51
1.42
1.41
1.39
1.38
1.36
1.34
1.25
1.21
N
O
8
4c
1.00
2.02
1.05
1.05
4.08
2.08
8.14
4.03
5.19
13.02
f1 (ppm)

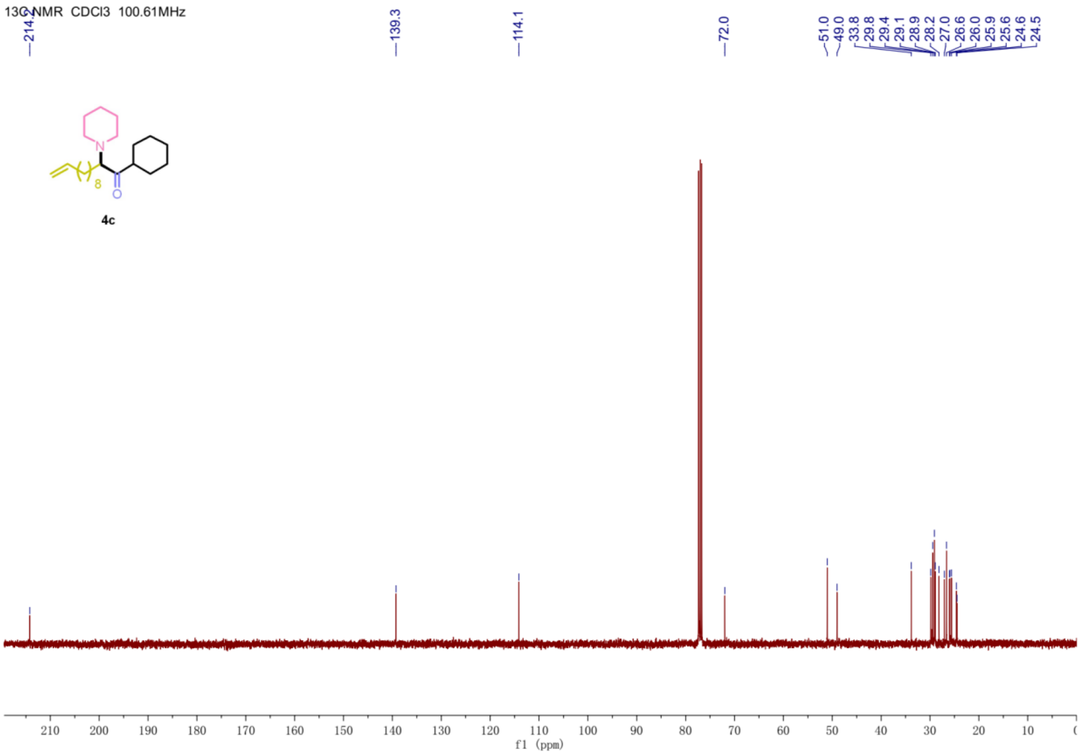
13C NMR CDCl3 100.61MHz
214.1
139.3
114.1
72.0
51.0
49.0
33.8
29.8
29.4
29.1
28.9
28.2
27.0
26.6
26.0
25.9
25.6
24.6
24.5
N
O
8
4c
f1 (ppm)

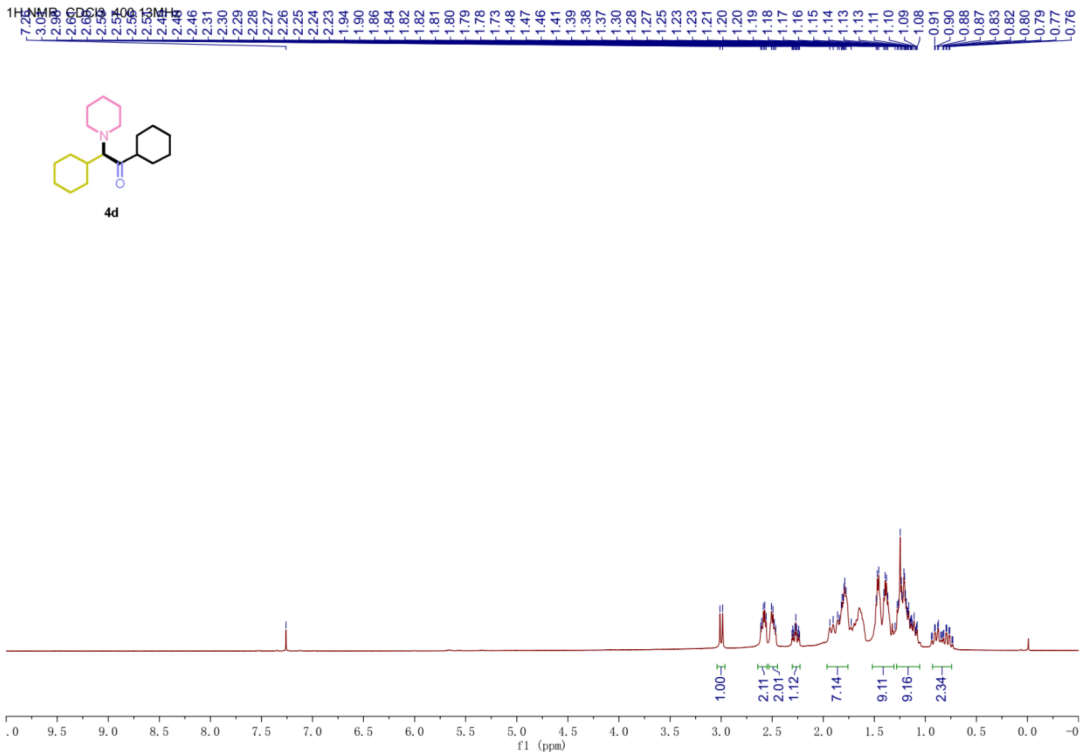
1H NMR CDCl3 400.13MHz
2.46 2.31 2.30 2.29 2.28 2.27 2.26 2.25 2.24 2.23 1.94 1.90 1.86 1.84 1.82 1.82 1.81 1.80 1.79 1.78 1.73 1.48 1.47 1.46 1.41 1.39 1.38 1.37 1.30 1.28 1.27 1.25 1.23 1.23 1.21 1.20 1.20 1.19 1.18 1.17 1.16 1.15 1.14 1.13 1.13 1.11 1.10 1.09 1.08 0.91 0.90 0.88 0.87 0.83 0.82 0.80 0.79 0.77 0.76
N
O
4d
1.00
2.11
2.01
1.12
7.14
9.11
9.16
2.34
9.5 9.0 8.5 8.0 7.5 7.0 6.5 6.0 5.5 5.0 4.5 4.0 3.5 3.0 2.5 2.0 1.5 1.0 0.5 0.0
f1 (ppm)

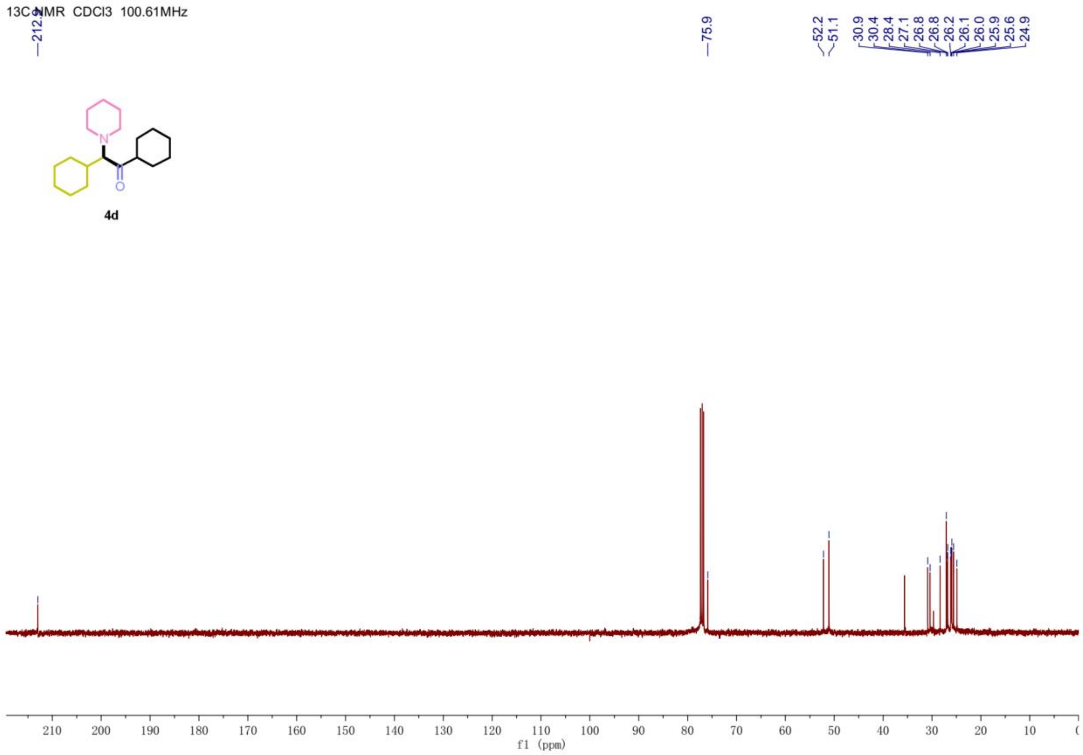
13C NMR CDCl3 100.61MHz
75.9
52.2
51.1
30.9 30.4 28.4 27.1 26.8 26.2 26.1 26.0 25.9 25.6 24.9
N
O
4d
210 200 190 180 170 160 150 140 130 120 110 100 90 80 70 60 50 40 30 20 10
f1 (ppm)

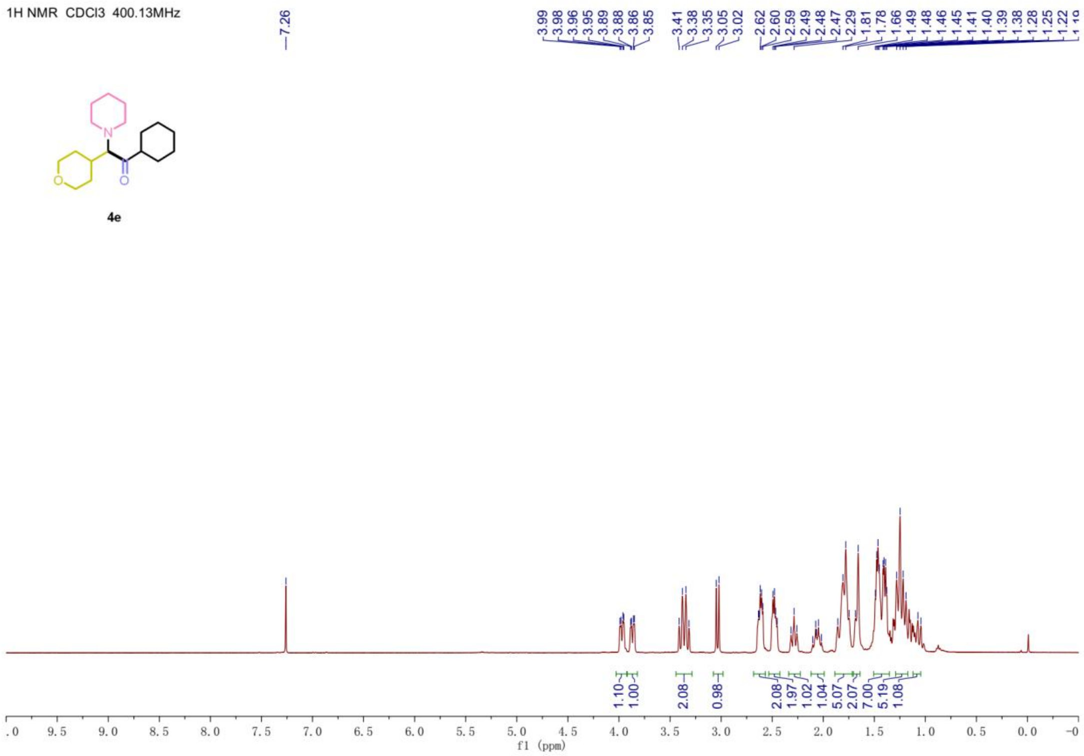
1H NMR CDCl3 400.13MHz
7.26
3.99 3.98 3.96 3.95 3.89 3.88 3.86 3.85
3.41 3.38 3.35 3.05 3.02
2.62 2.60 2.59 2.49 2.48 2.47 2.29 1.81 1.78 1.66 1.49 1.48 1.46 1.45 1.41 1.40 1.39 1.38 1.28 1.25 1.22
4e
1.10 1.00 2.08 0.98 2.08 1.97 1.02 1.04 5.07 2.07 7.00 5.19 1.08
f1 (ppm)

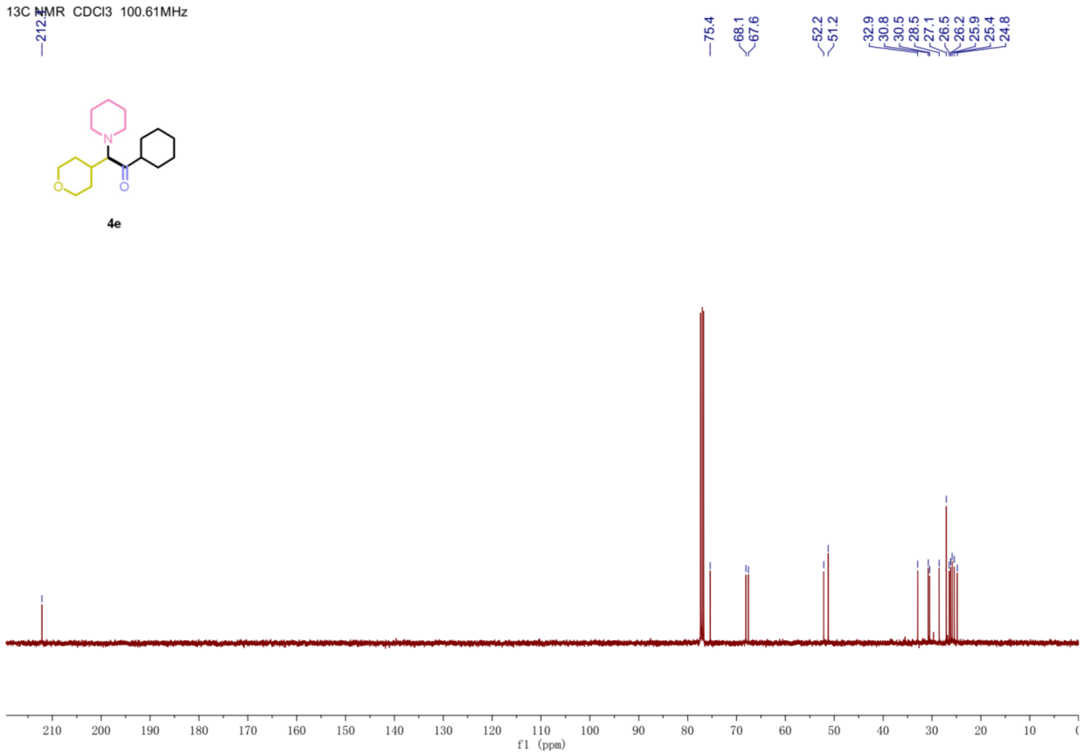
13C NMR CDCl3 100.61MHz
212.
75.4
68.1 67.6
52.2 51.2
32.9 30.8 30.5 28.5 27.1 26.5 26.2 25.9 25.4 24.8
4e
f1 (ppm)

1H NMR CDCl3 400.13MHz

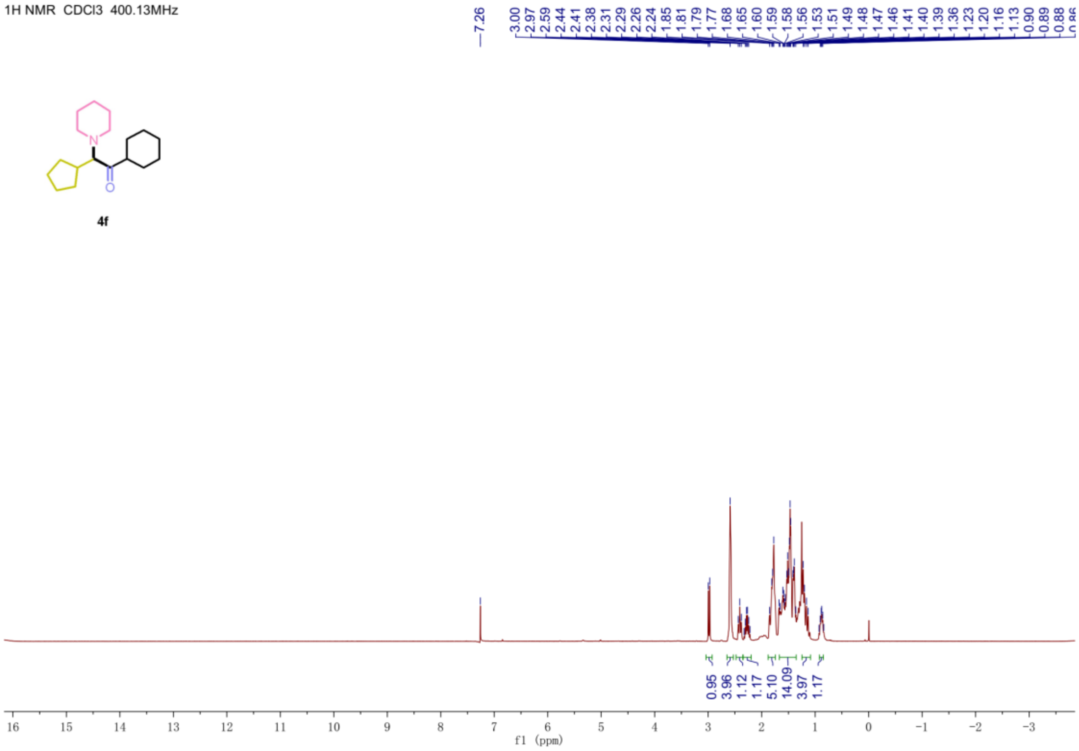

13C NMR CDCl3 100.61MHz

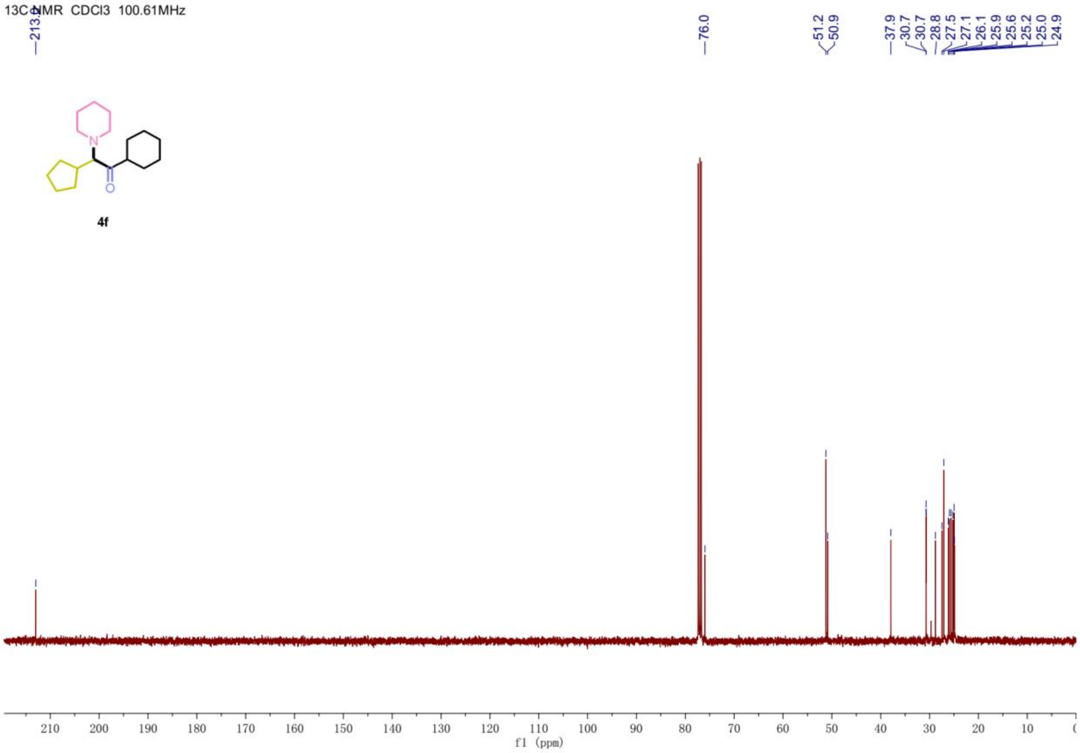

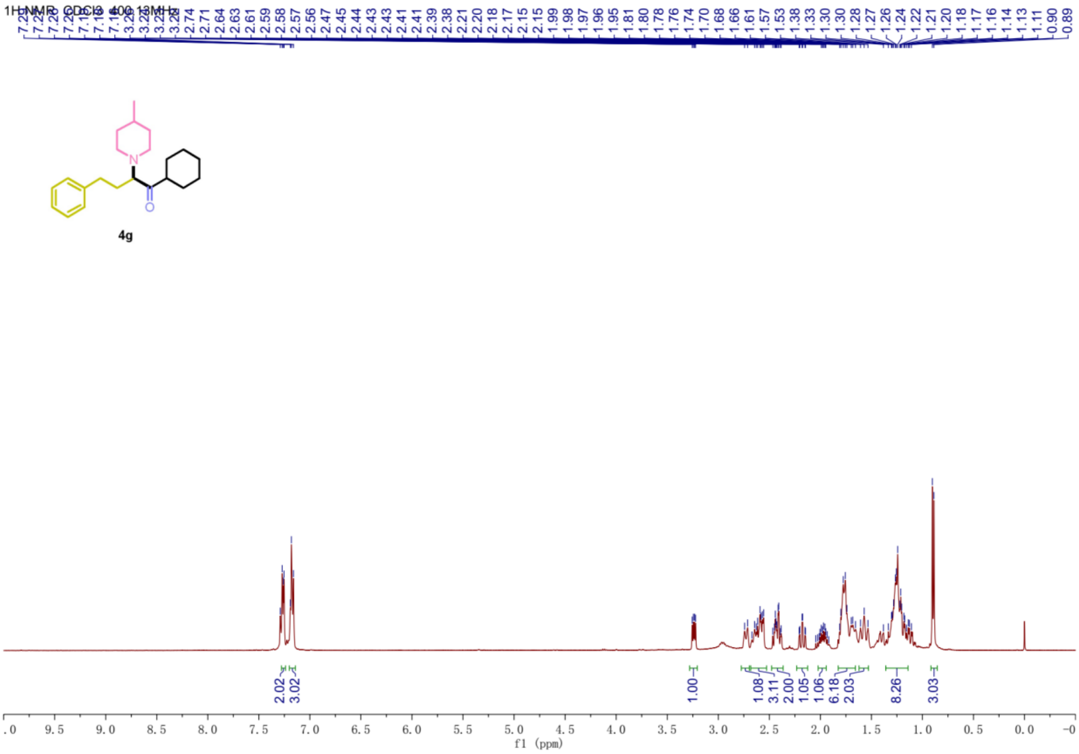
1H-NMR CDCl3 400.13MHz
4g
2.02
3.02
1.00
1.08
3.11
2.00
1.05
1.06
6.18
2.03
8.26
3.03
f1 (ppm)

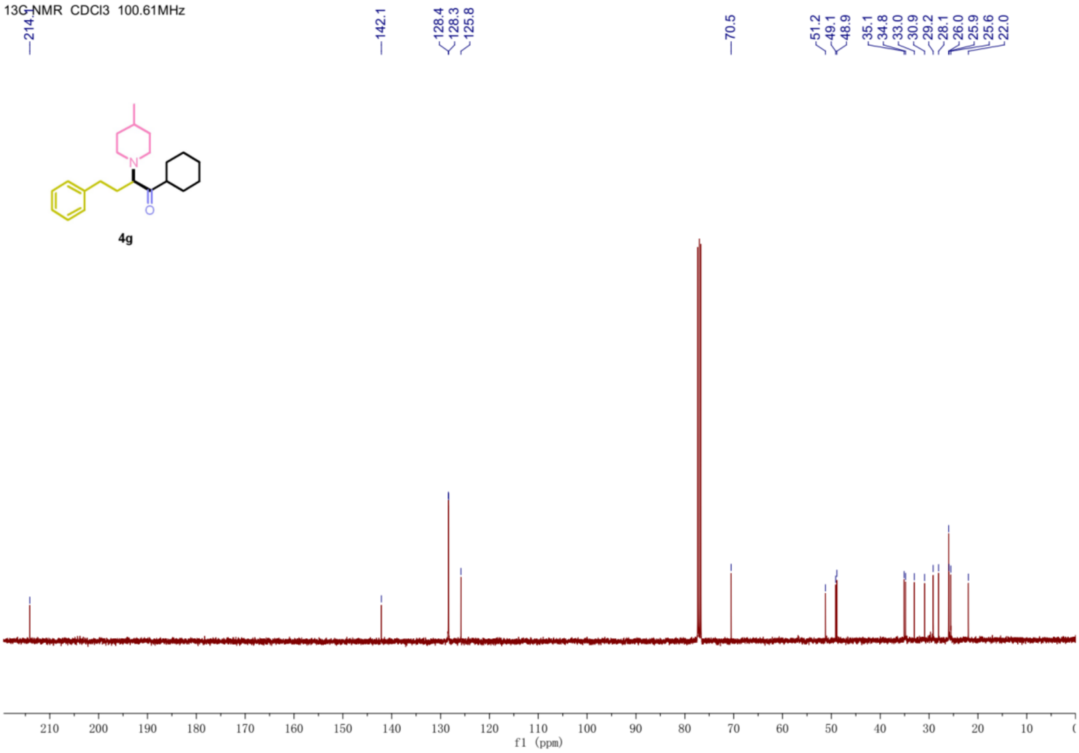
13C-NMR CDCl3 100.61MHz
214.1
142.1
128.4
128.3
125.8
70.5
51.2
49.1
48.9
35.1
34.8
33.0
30.9
29.2
28.1
26.0
25.9
25.6
22.0
4g
f1 (ppm)

1H NMR CDCl3 400.13MHz

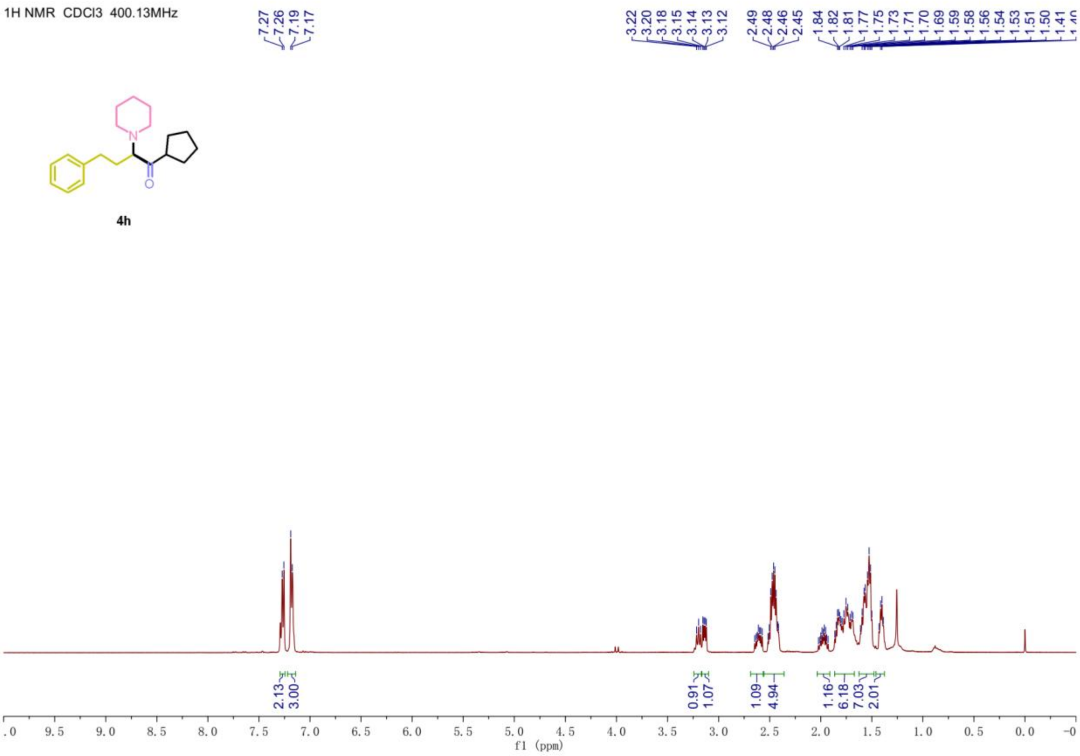

13C-NMR CDCl3 100.61MHz

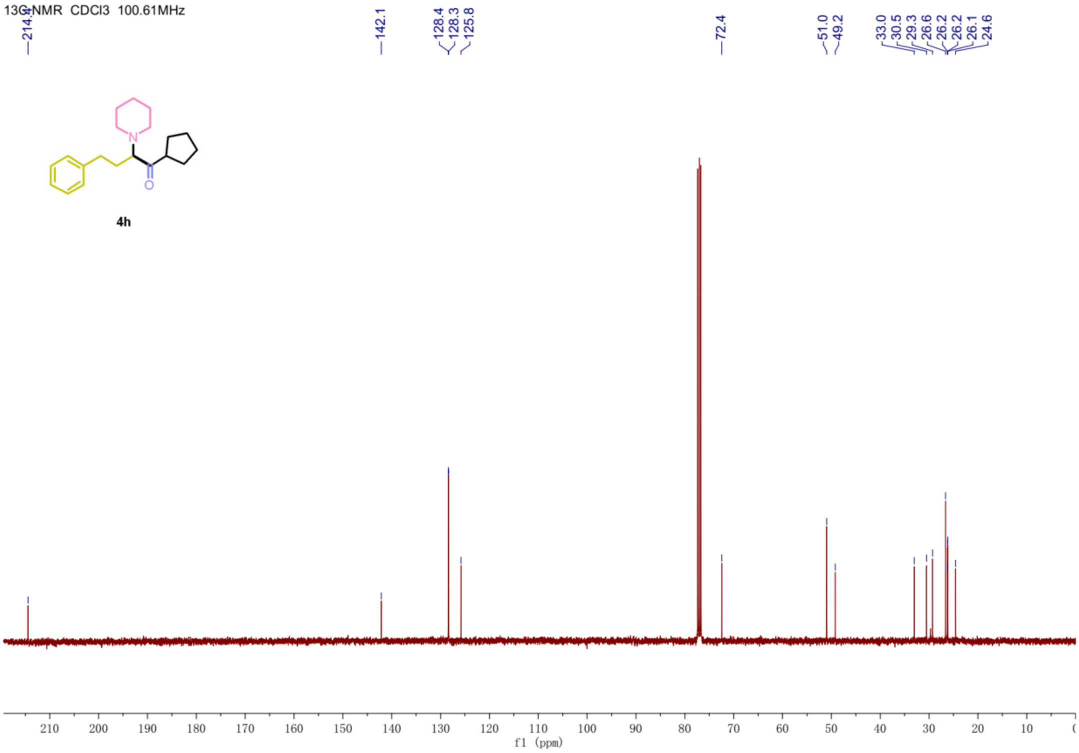

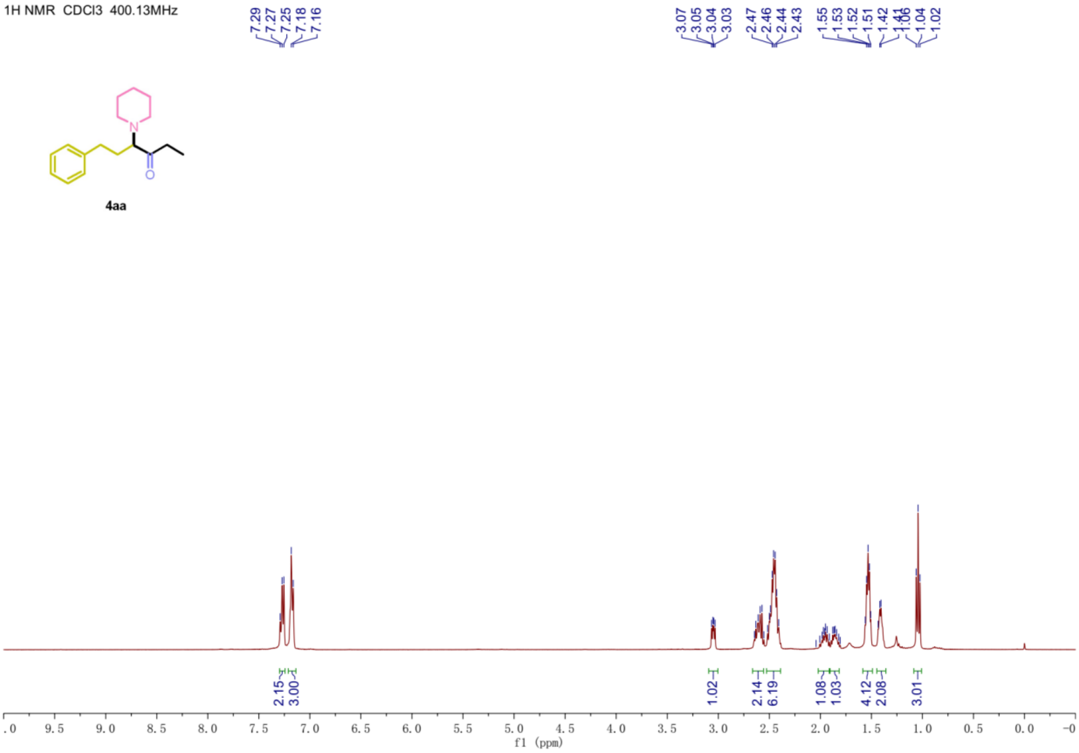
1H NMR CDCl3 400.13MHz
7.29 7.27 7.25 7.18 7.16
3.07 3.05 3.04 3.03 2.47 2.46 2.44 2.43 1.55 1.53 1.52 1.51 1.42 1.41 1.06 1.04 1.02
4aa
2.15 3.00 1.02 2.14 6.19 1.08 1.03 4.12 2.08 3.01
f1 (ppm)

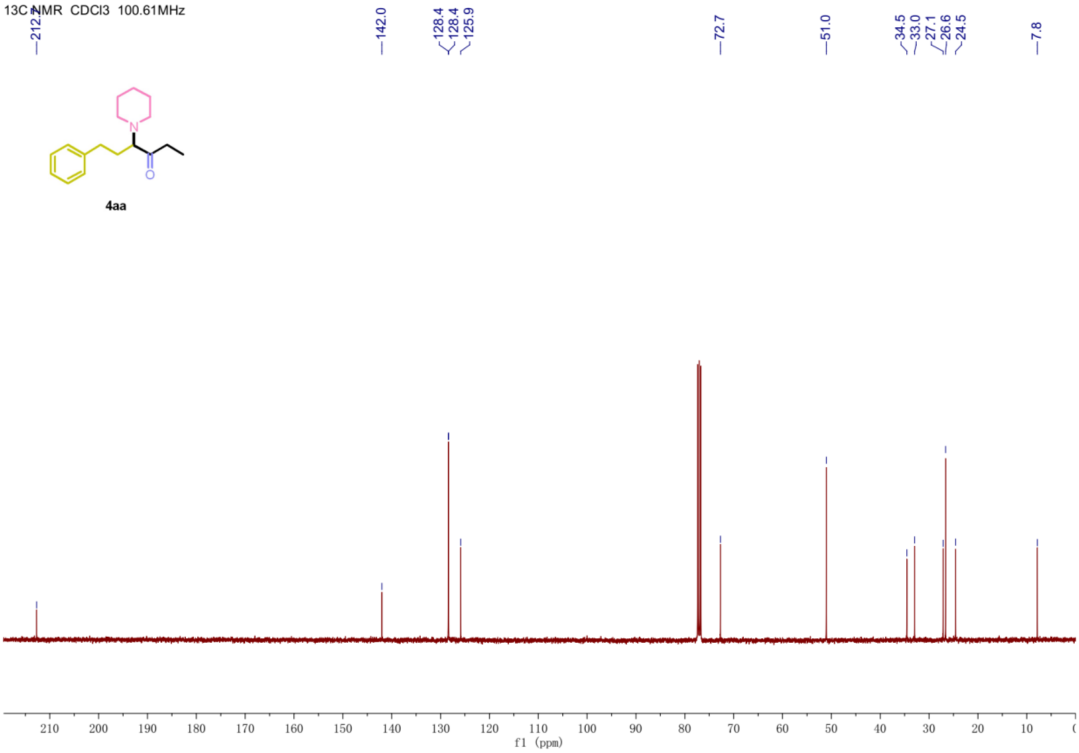
13C NMR CDCl3 100.61MHz
212.4
142.0
128.4 128.4 125.9
72.7
51.0
34.5 33.0 27.1 26.6 24.5
7.8
4aa
f1 (ppm)

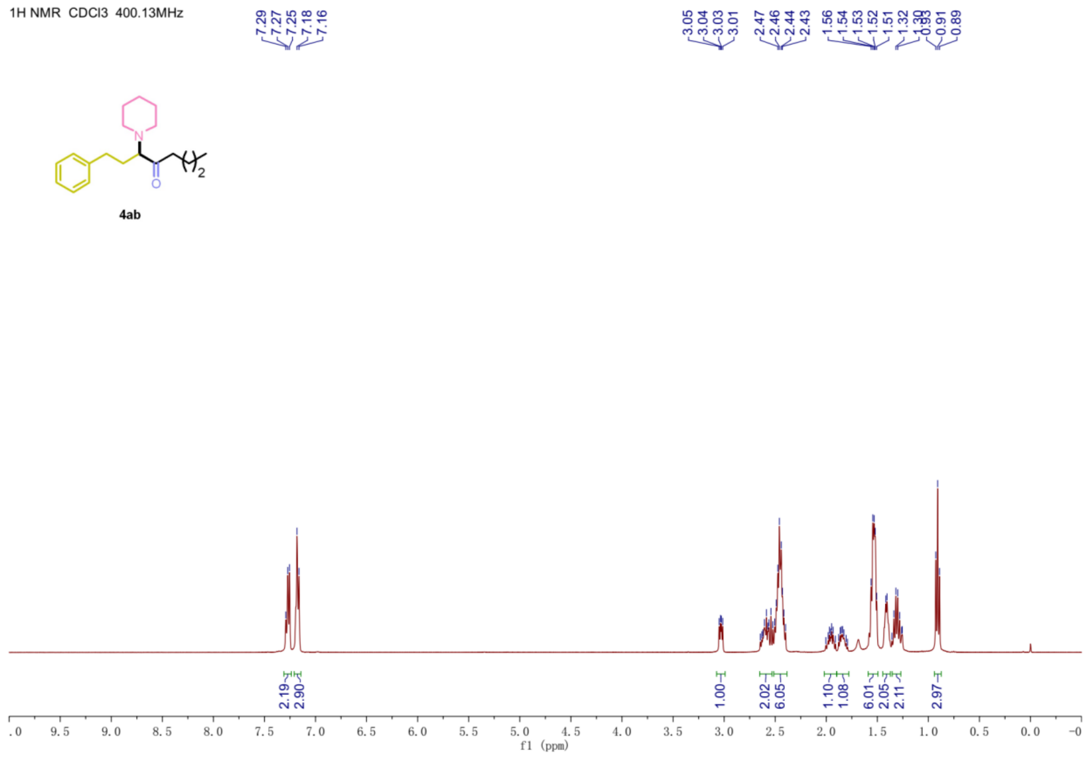
1H NMR CDCl3 400.13MHz
7.29
7.27
7.25
7.18
7.16
3.05
3.04
3.03
3.01
2.47
2.46
2.44
2.43
1.56
1.54
1.53
1.52
1.51
1.32
0.91
0.89
4ab
2.19
2.90
1.00
2.02
6.05
1.10
1.08
6.01
2.05
2.11
2.97
f1 (ppm)

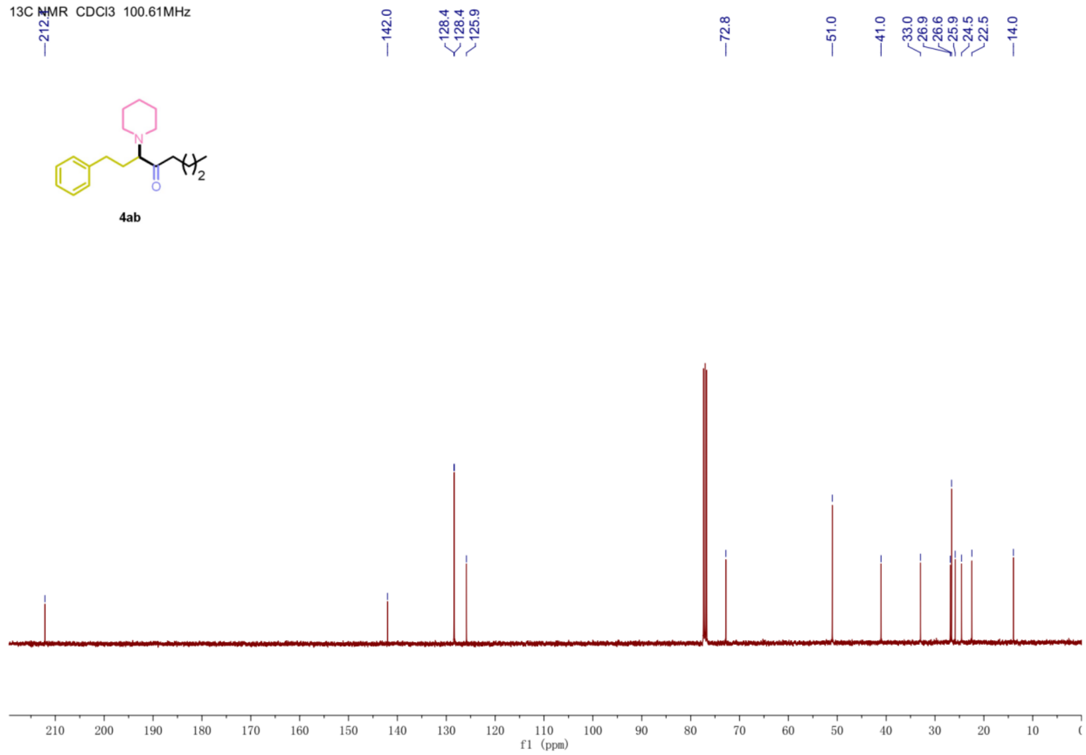
13C NMR CDCl3 100.61MHz
212.
142.0
128.4
128.4
125.9
72.8
51.0
41.0
33.0
26.9
26.6
25.9
24.5
22.5
14.0
4ab
f1 (ppm)

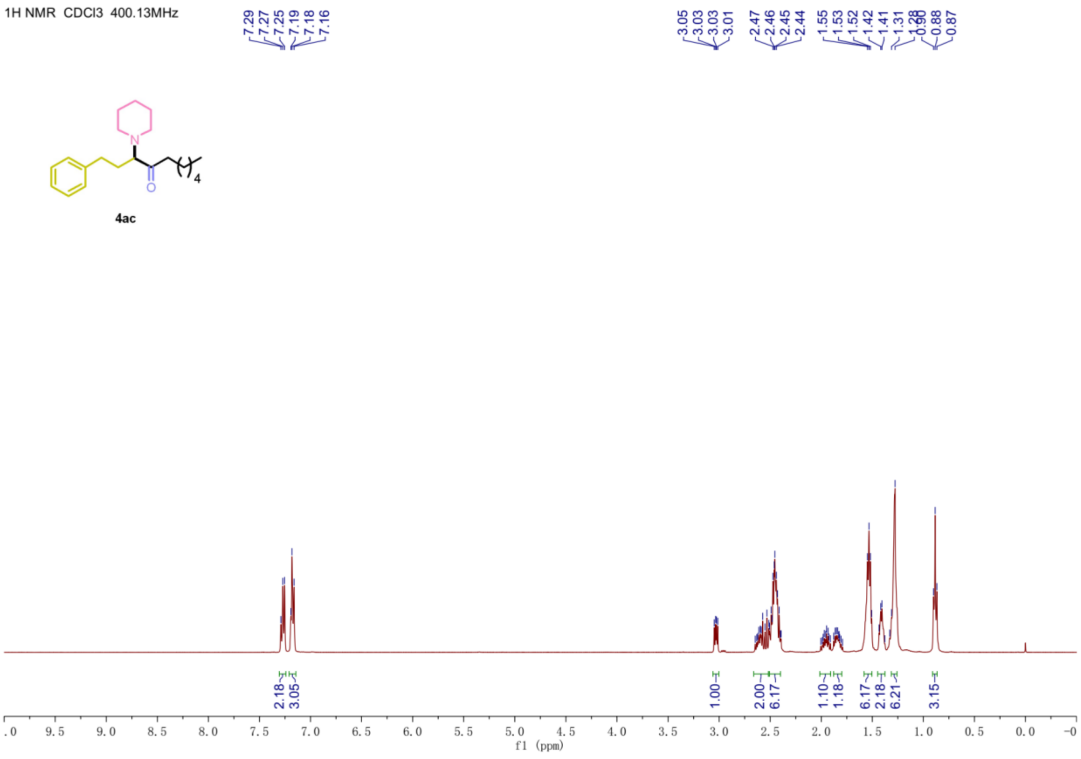
1H NMR CDCl3 400.13MHz
7.29 7.27 7.25 7.19 7.18 7.16
3.05 3.03 3.03 3.01 2.47 2.46 2.45 2.44 1.55 1.53 1.52 1.42 1.41 1.31 0.88 0.87
N
O
4
4ac
2.18 3.05 1.00 2.00 6.17 1.10 1.18 6.17 2.18 6.21 3.15
f1 (ppm)

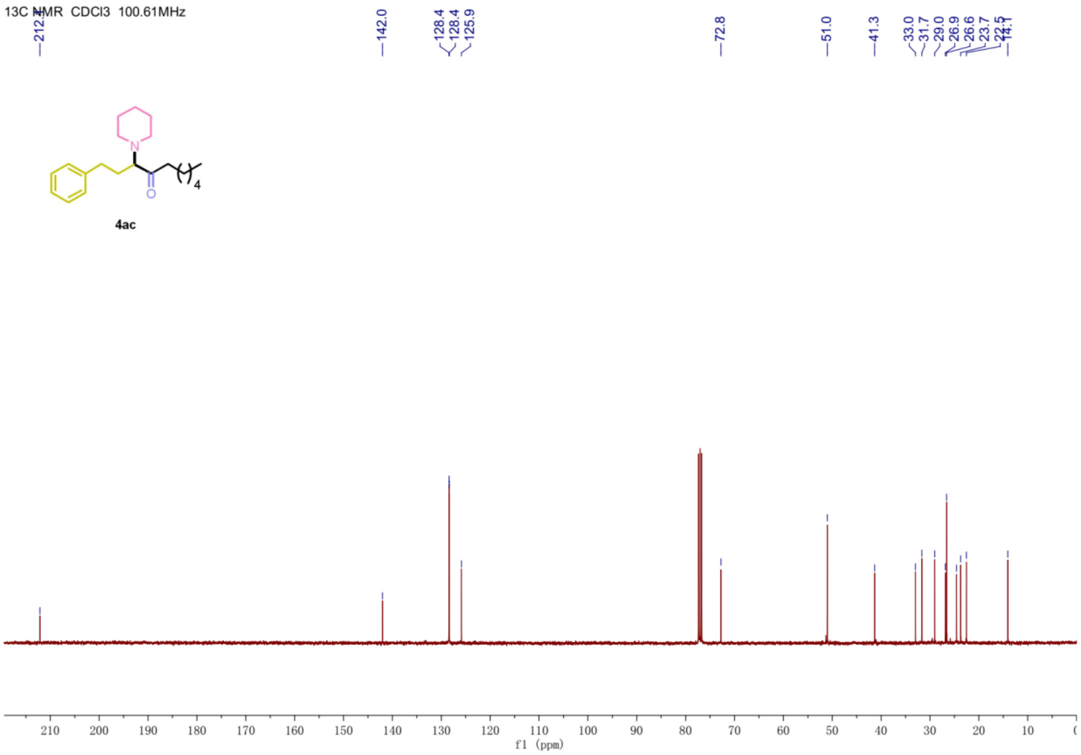
13C NMR CDCl3 100.61MHz
212.
142.0
128.4 128.4 125.9
72.8
51.0
41.3
33.0 31.7 29.0 26.9 26.6 23.7 22.5 14.1
N
O
4
4ac
f1 (ppm)

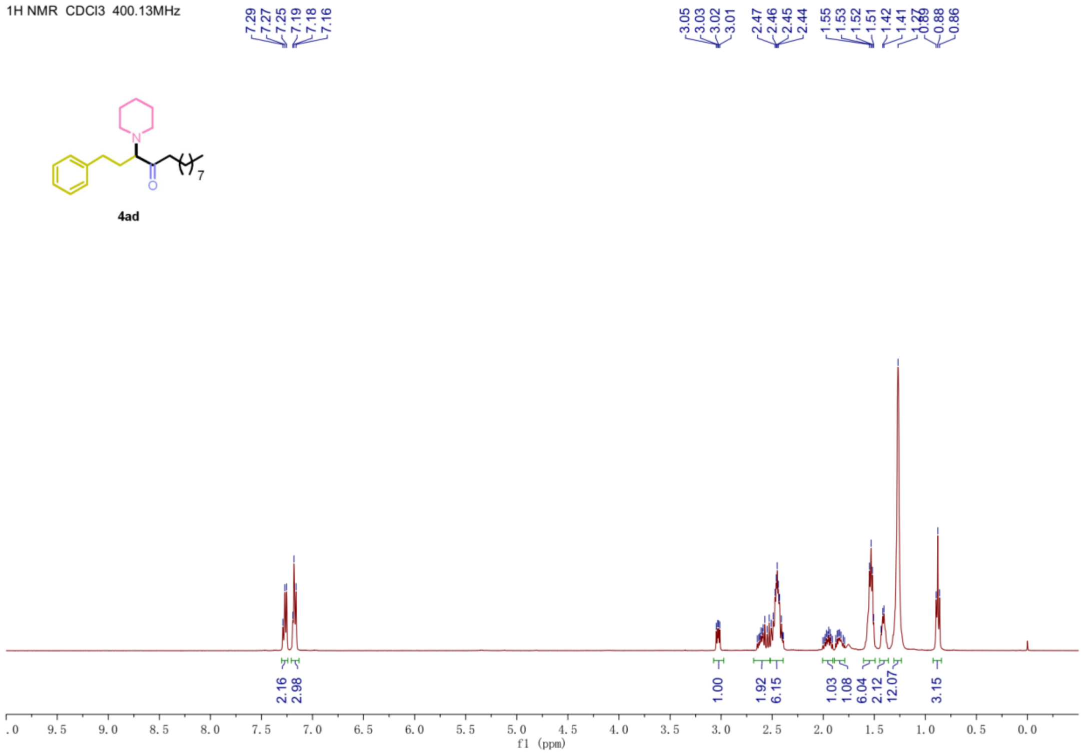
1H NMR CDCl3 400.13MHz
7.29
7.27
7.25
7.19
7.18
7.16
3.05
3.03
3.02
3.01
2.47
2.46
2.45
2.44
1.55
1.53
1.52
1.51
1.42
1.41
0.88
0.86
4ad
2.16
2.98
1.00
1.92
6.15
1.03
1.08
6.04
2.12
12.07
3.15
f1 (ppm)

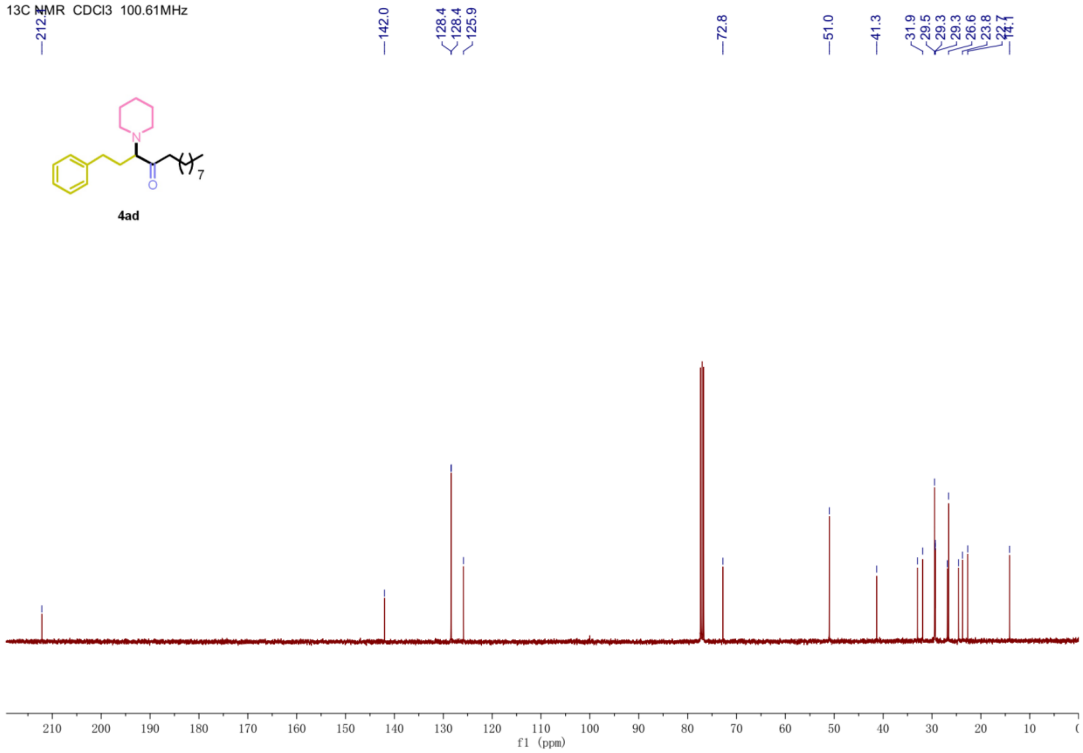
13C NMR CDCl3 100.61MHz
142.0
128.4
128.4
125.9
72.8
51.0
41.3
31.9
29.5
29.3
29.3
26.6
23.8
22.7
14.1
4ad
f1 (ppm)

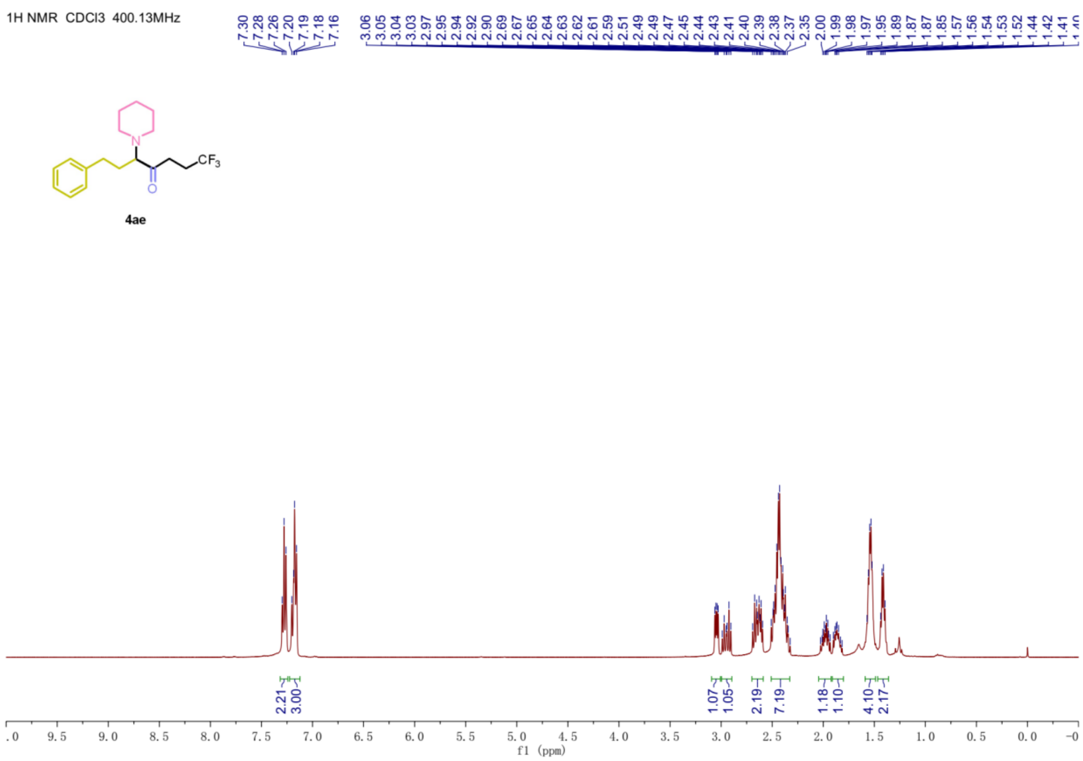
1H NMR CDCl3 400.13MHz
4ae
f1 (ppm)

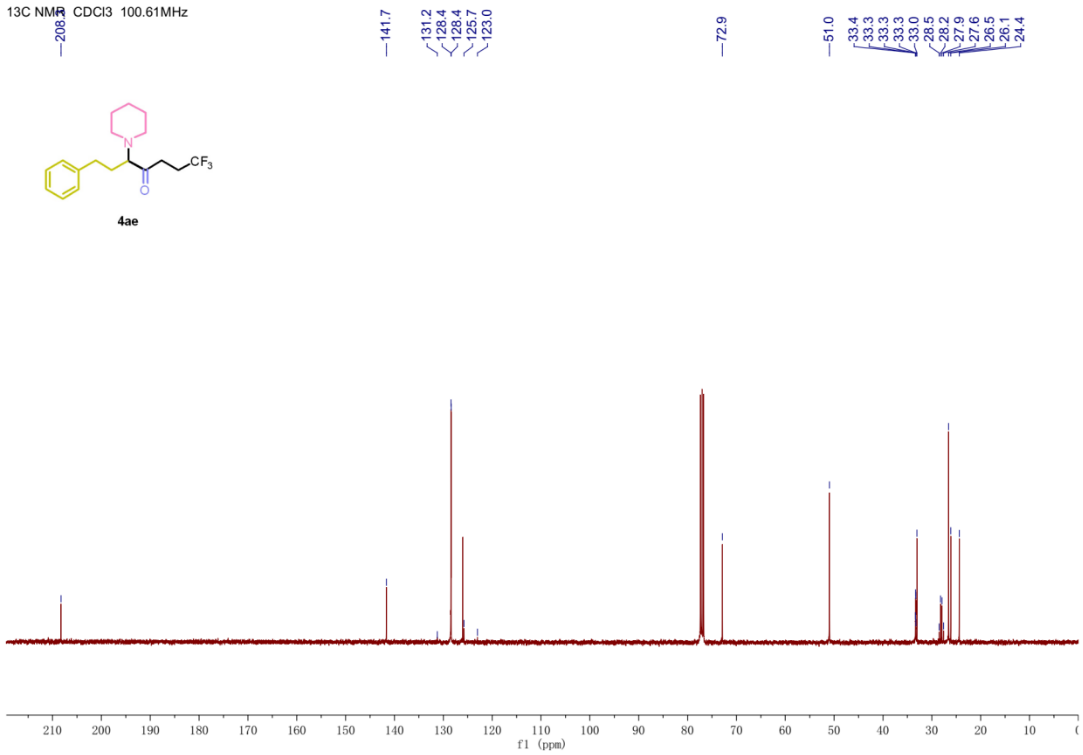
13C NMR CDCl3 100.61MHz
4ae
f1 (ppm)

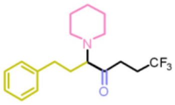

4ae

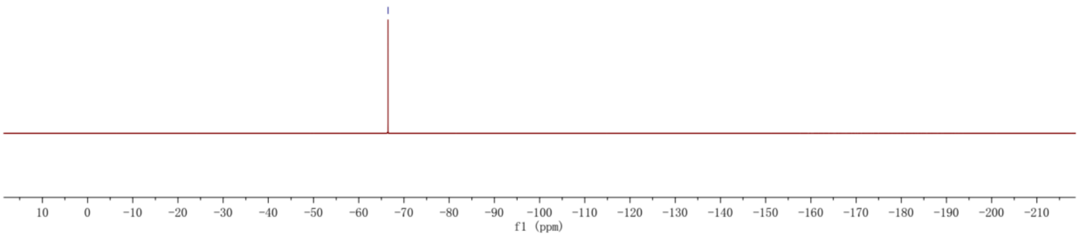
19F NMR CDCl3 376.50MHz
-66.5
10 0 −10 −20 −30 −40 −50 −60 −70 −80 −90 −100 −110 −120 −130 −140 −150 −160 −170 −180 −190 −200 −210
f1 (ppm)

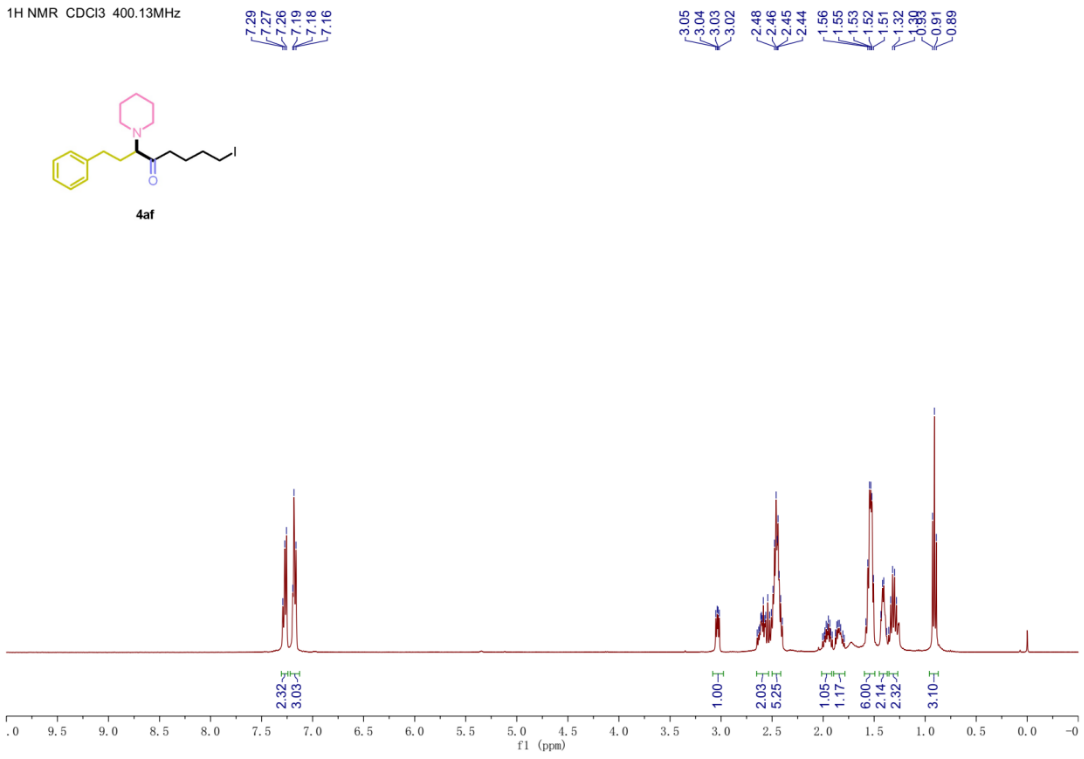
1H NMR CDCl3 400.13MHz
7.29
7.27
7.26
7.19
7.18
7.16
3.05
3.04
3.03
3.02
2.48
2.46
2.45
2.44
1.56
1.55
1.53
1.52
1.51
1.32
0.91
0.89
4af
2.32
3.03
1.00
2.03
5.25
1.05
1.17
6.00
2.14
2.32
3.10
f1 (ppm)

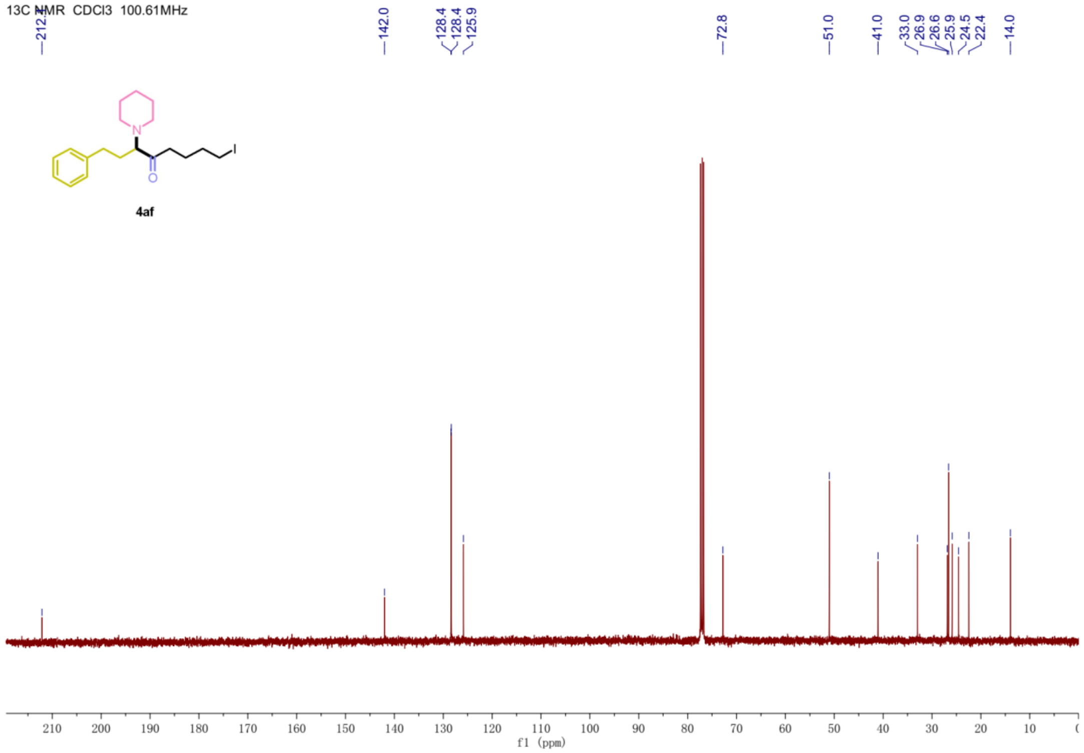
13C NMR CDCl3 100.61MHz
212.1
142.0
128.4
128.4
125.9
72.8
51.0
41.0
33.0
26.9
26.6
25.9
24.5
22.4
14.0
4af
f1 (ppm)

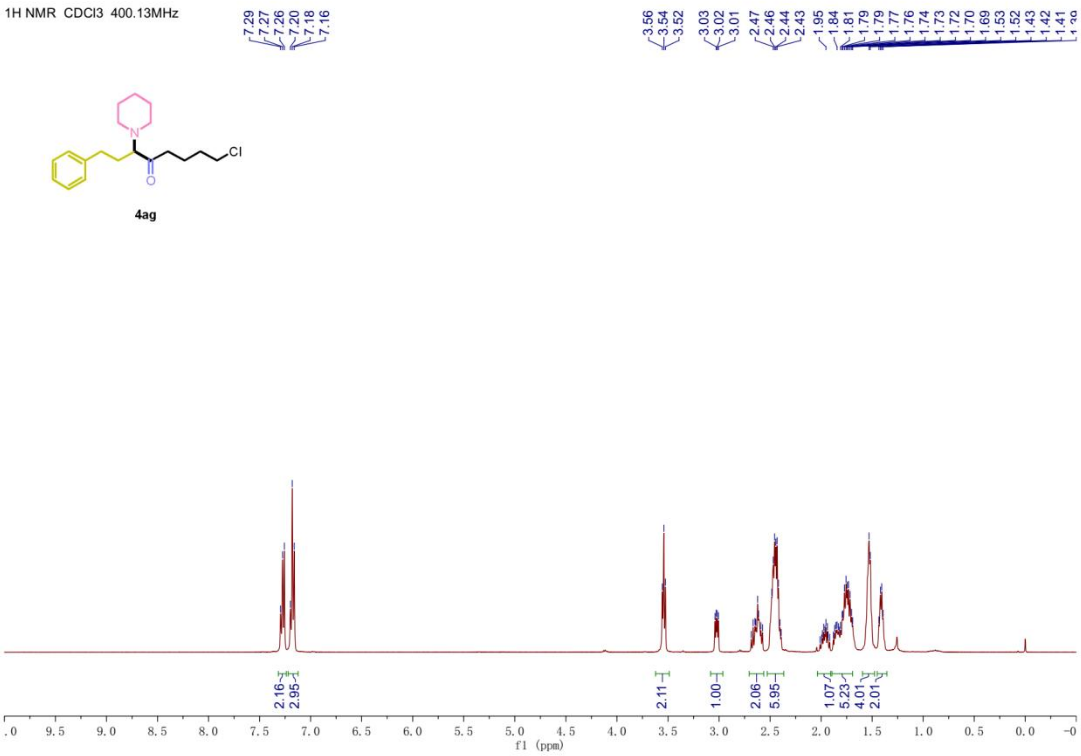
1H NMR CDCl3 400.13MHz
4ag
f1 (ppm)

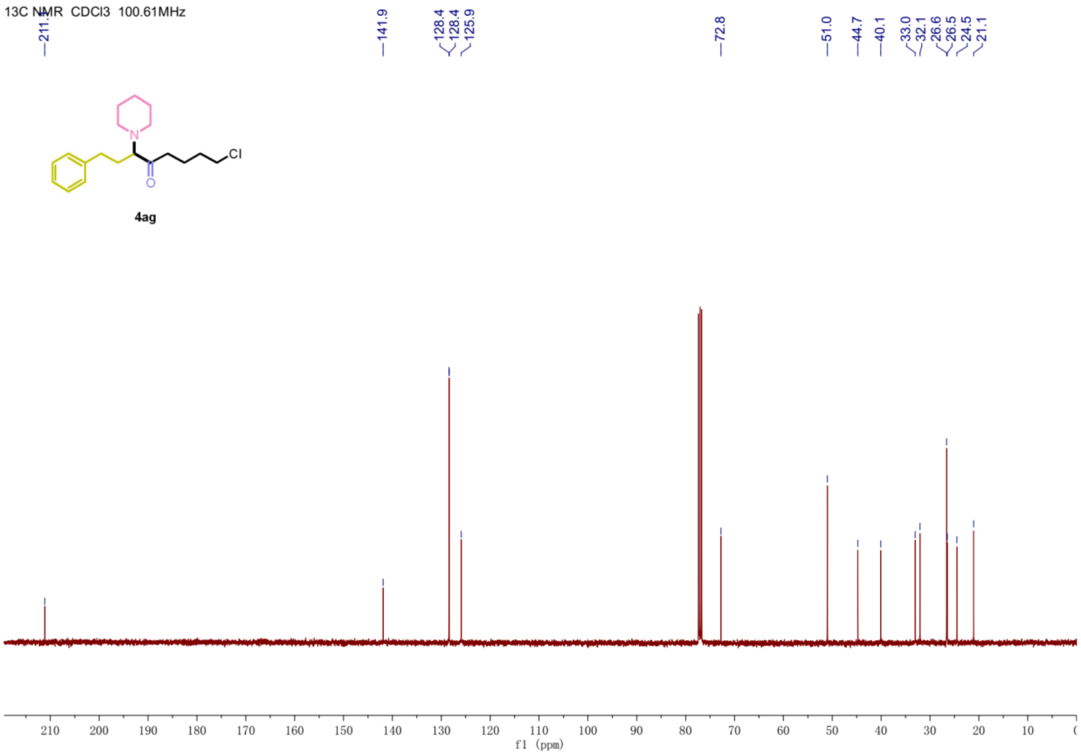
13C NMR CDCl3 100.61MHz
4ag
f1 (ppm)

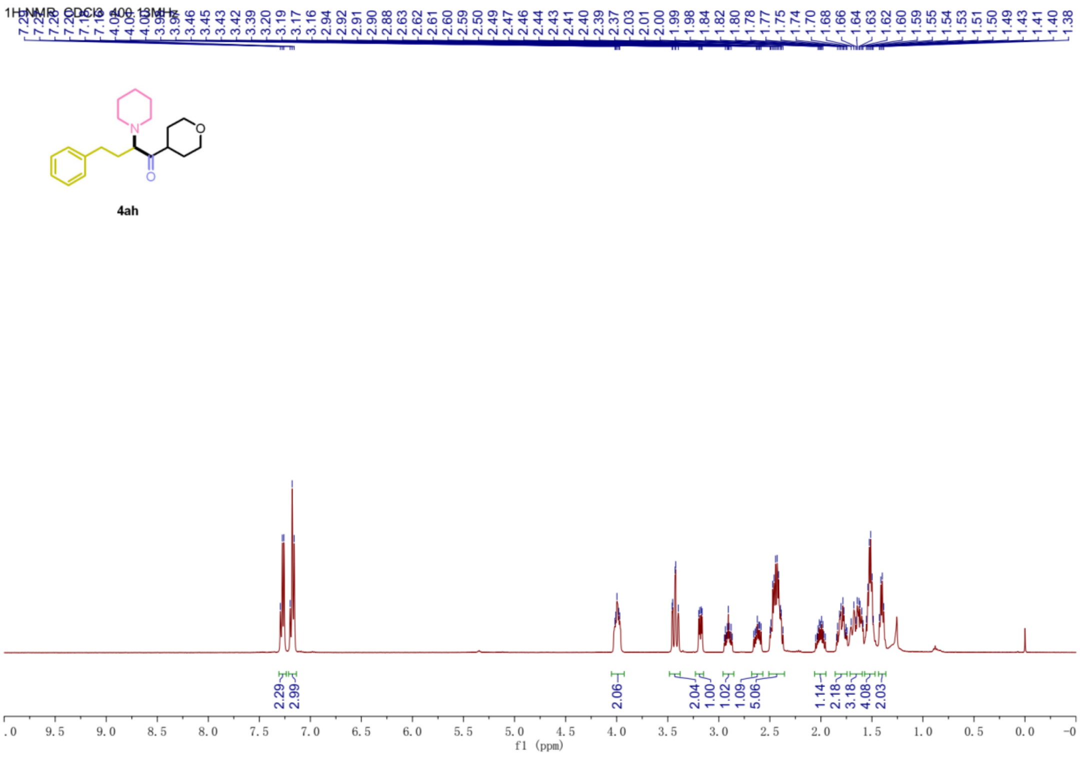
1H NMR CDCl3 400.13MHz
4ah
f1 (ppm)

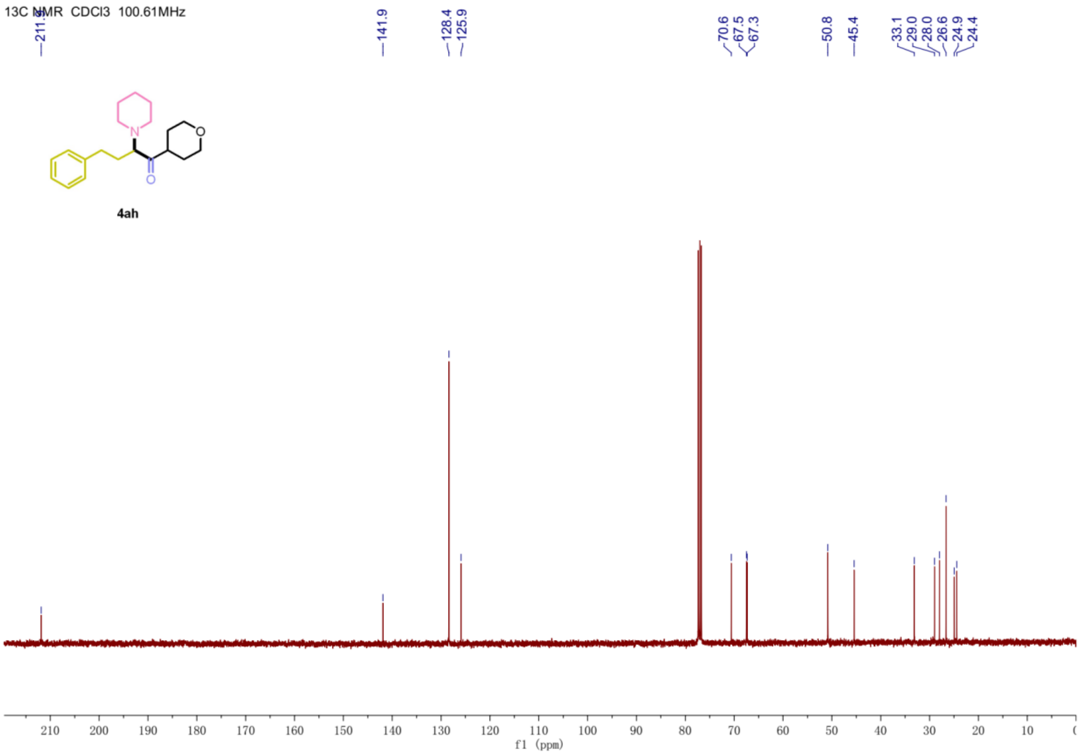
13C NMR CDCl3 100.61MHz
211.9
141.9
128.4
125.9
70.6
67.5
67.3
50.8
45.4
33.1
29.0
28.0
26.6
24.9
24.4
4ah
f1 (ppm)

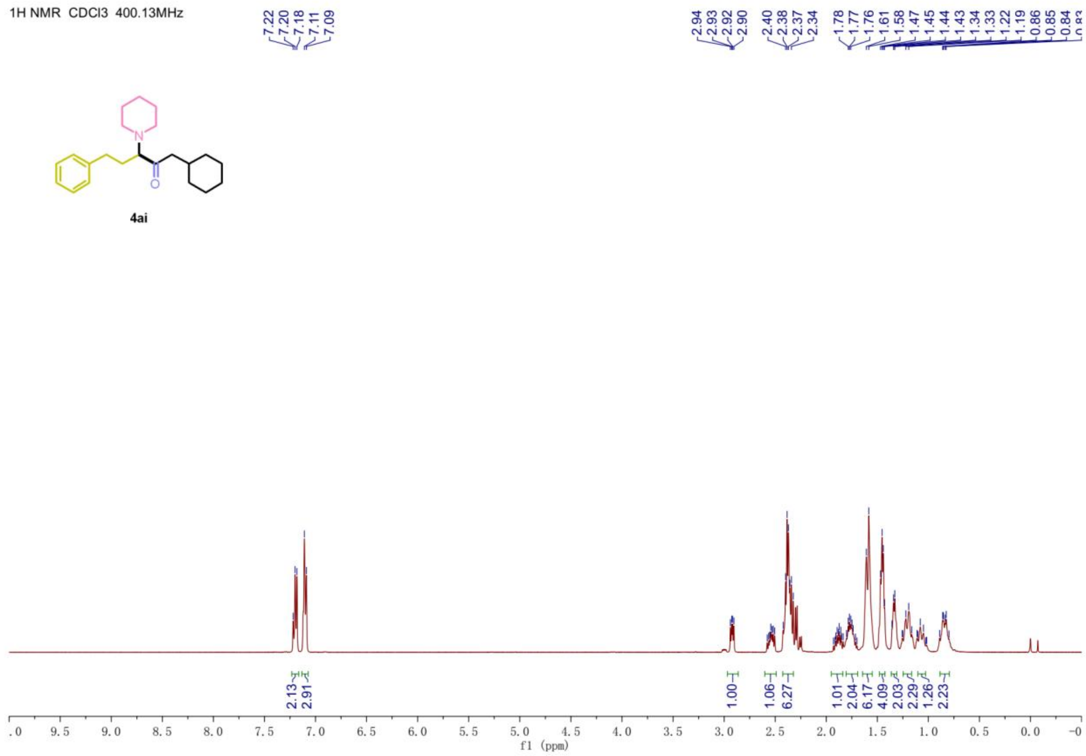
1H NMR CDCl3 400.13MHz
7.22
7.20
7.18
7.11
7.09
2.94
2.93
2.92
2.90
2.40
2.38
2.37
2.34
1.78
1.77
1.76
1.61
1.58
1.47
1.45
1.44
1.43
1.34
1.33
1.22
1.19
0.86
0.85
0.84
N
O
4ai
2.13
2.91
1.00
1.06
6.27
1.01
2.04
6.17
4.09
2.03
2.29
1.26
2.23
f1 (ppm)

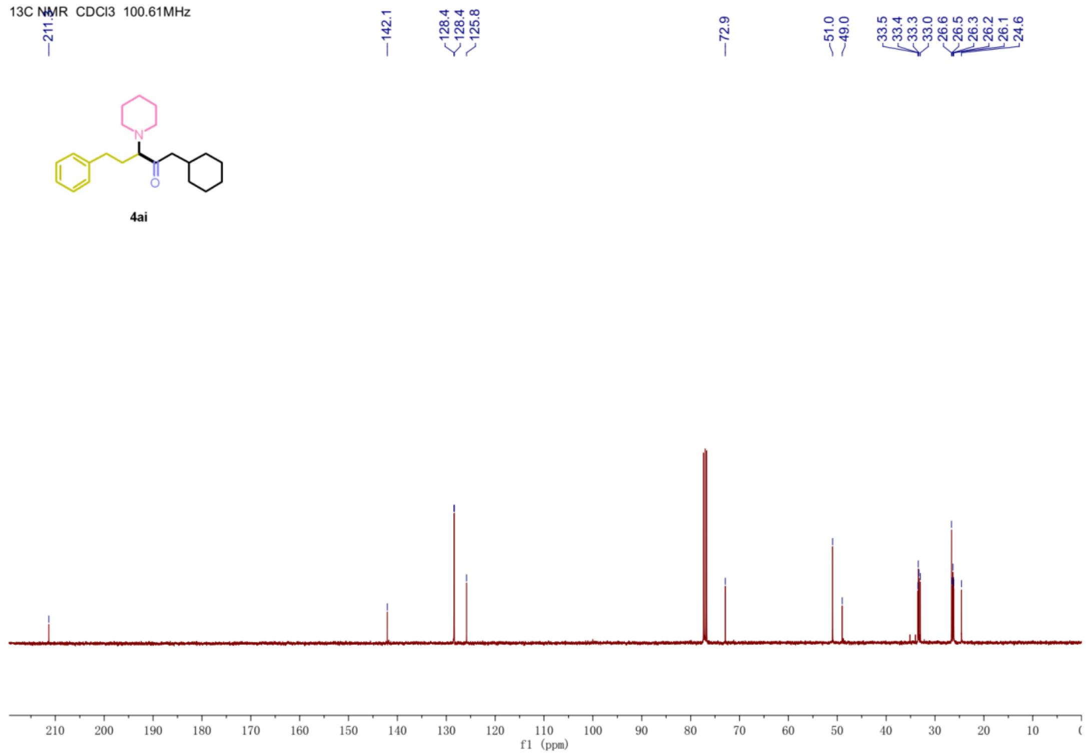
13C NMR CDCl3 100.61MHz
211.
142.1
128.4
128.4
125.8
72.9
51.0
49.0
33.5
33.4
33.3
33.0
26.6
26.5
26.3
26.2
26.1
24.6
N
O
4ai
f1 (ppm)

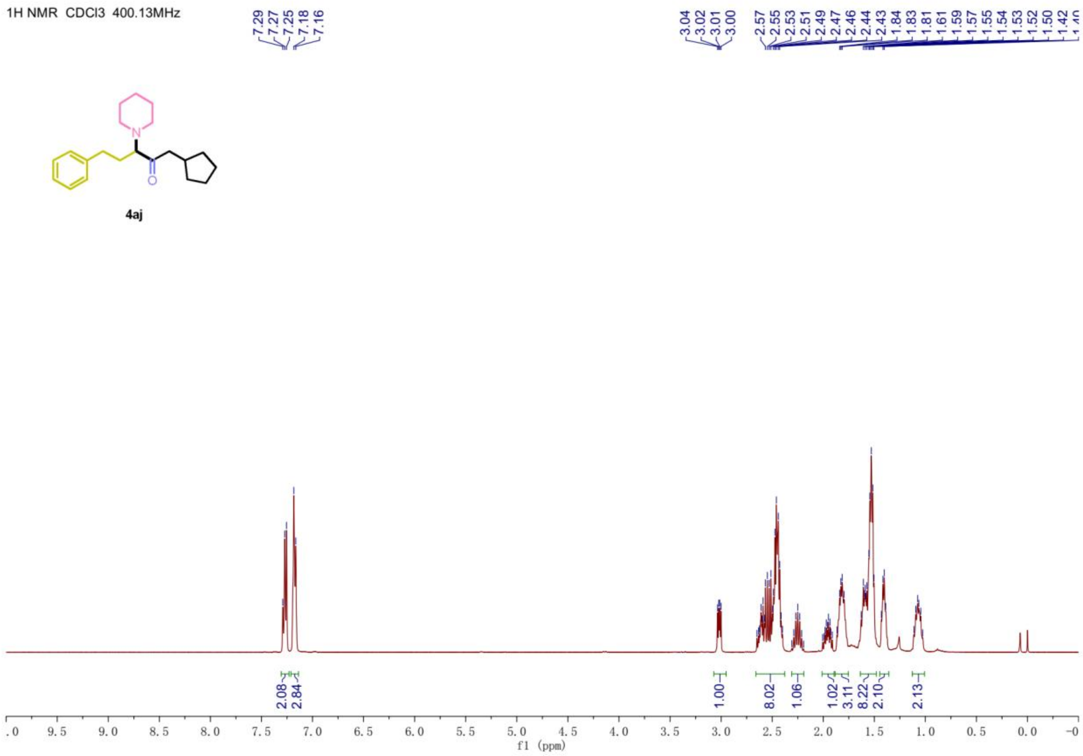
1H NMR CDCl3 400.13MHz
7.29
7.27
7.25
7.18
7.16
3.04
3.02
3.01
3.00
2.57
2.55
2.53
2.51
2.49
2.47
2.46
2.44
2.43
1.84
1.83
1.81
1.61
1.59
1.57
1.55
1.54
1.53
1.52
1.50
1.42
4aj
2.08
2.84
1.00
8.02
1.06
1.02
3.11
8.22
2.10
2.13
f1 (ppm)

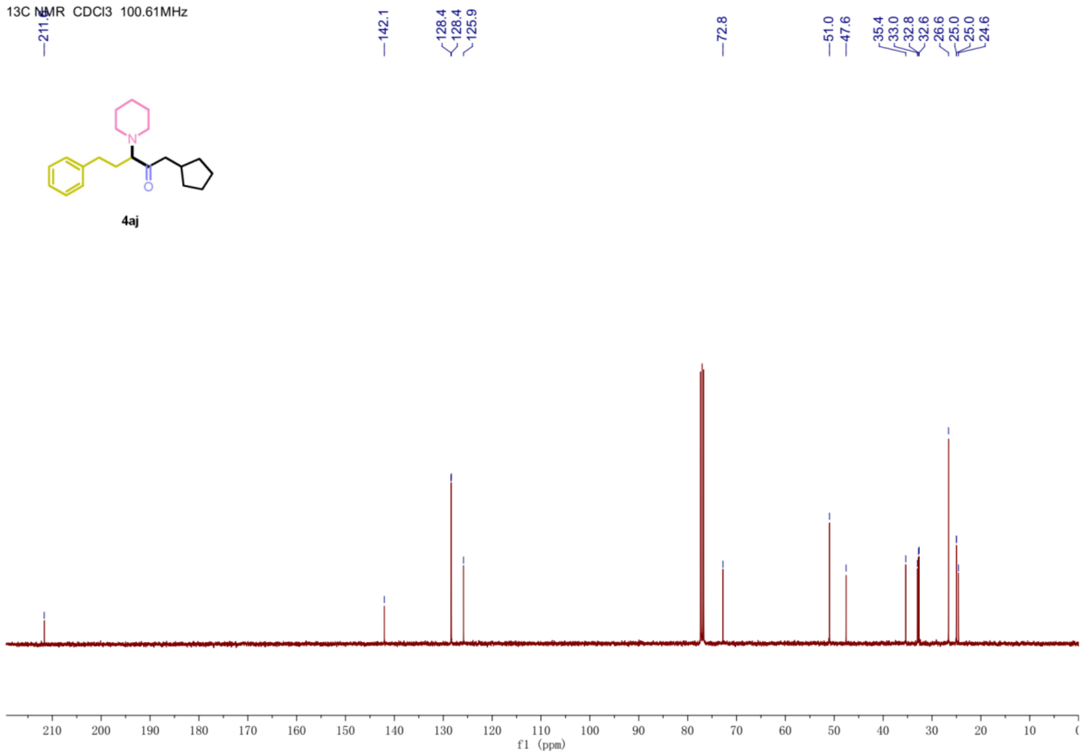
13C NMR CDCl3 100.61MHz
142.1
128.4
128.4
125.9
72.8
51.0
47.6
35.4
33.0
32.8
32.6
26.6
25.0
25.0
24.6
4aj
f1 (ppm)

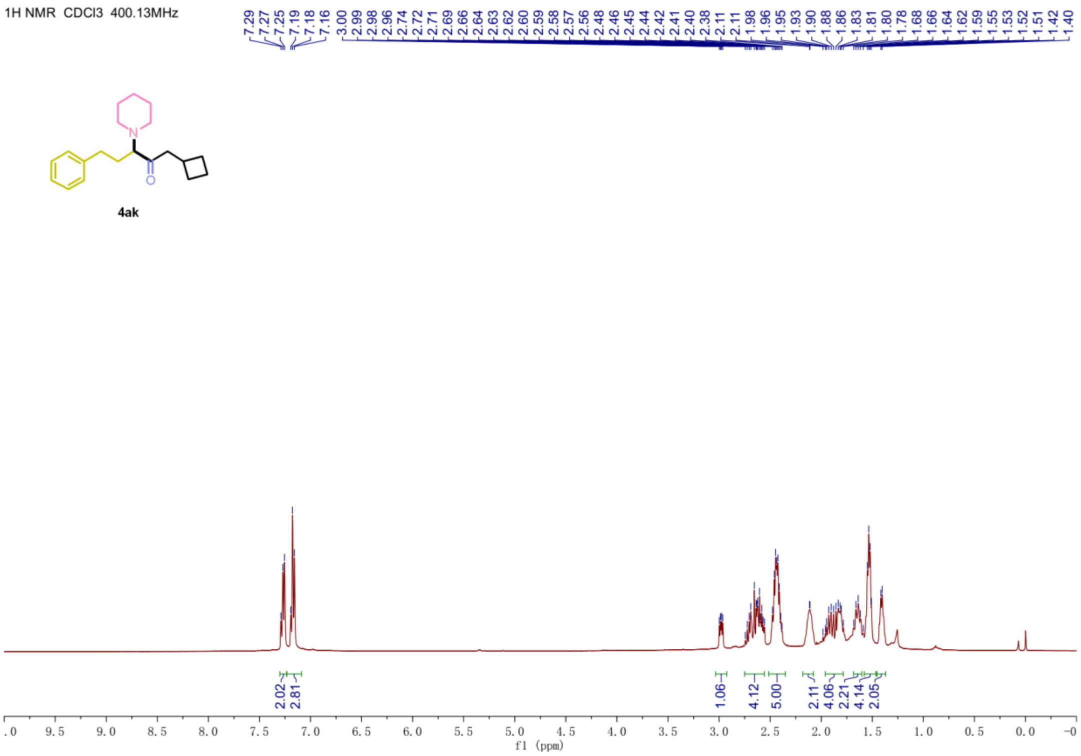
1H NMR CDCl3 400.13MHz
7.29 7.27 7.25 7.19 7.18 7.16 3.00 2.99 2.98 2.96 2.74 2.72 2.71 2.69 2.66 2.64 2.63 2.62 2.60 2.59 2.58 2.57 2.56 2.48 2.46 2.45 2.44 2.42 2.41 2.40 2.38 2.11 1.98 1.96 1.95 1.93 1.90 1.88 1.86 1.83 1.81 1.80 1.78 1.68 1.66 1.64 1.62 1.59 1.55 1.53 1.52 1.51 1.42 1.40
N
O
4ak
2.02 2.81 1.06 4.12 5.00 2.11 4.06 2.21 4.14 2.05
f1 (ppm)

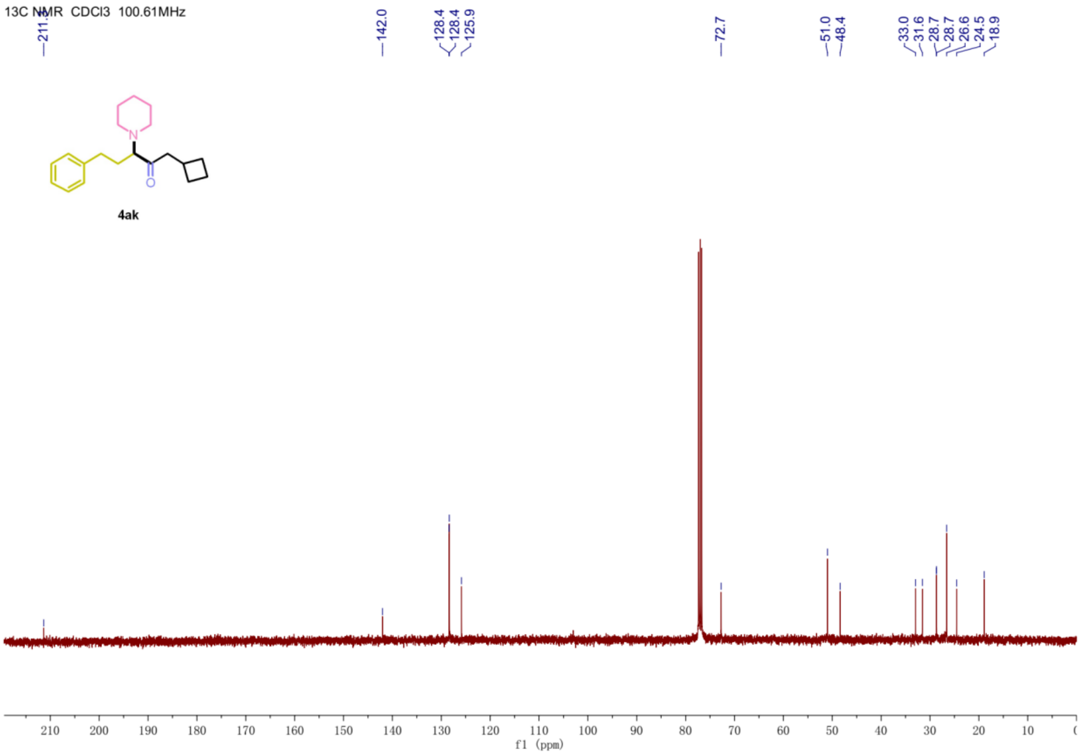
13C NMR CDCl3 100.61MHz
211
142.0 128.4 128.4 125.9 72.7 51.0 48.4 33.0 31.6 28.7 26.6 24.5 18.9
N
O
4ak
f1 (ppm)

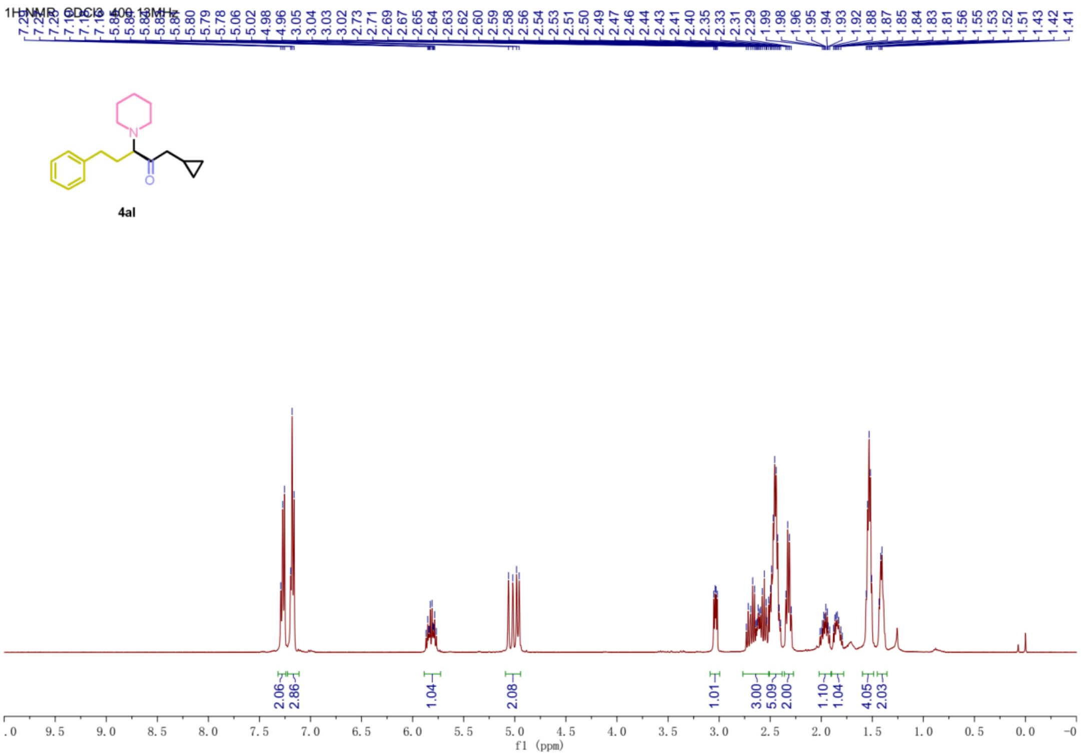
1H NMR CDCl3 400.13MHz
4al
2.06
2.86
1.04
2.08
1.01
3.00
5.09
2.00
1.10
1.04
4.05
2.03
f1 (ppm)

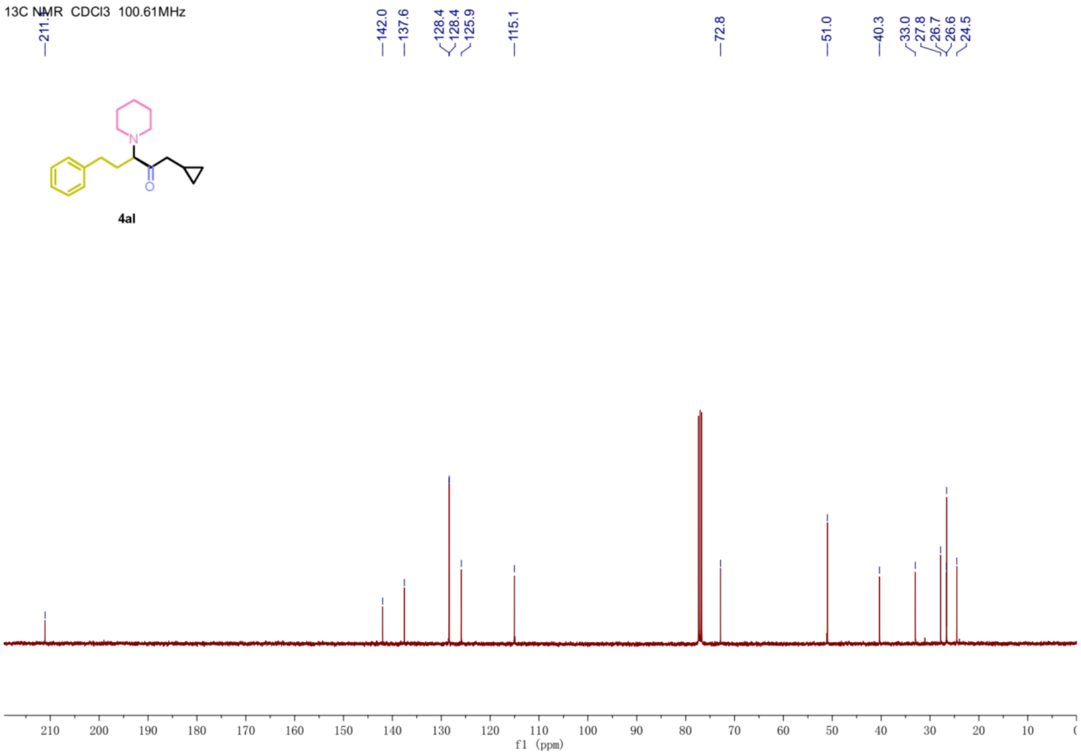
13C NMR CDCl3 100.61MHz
211.1
142.0
137.6
128.4
128.4
125.9
115.1
72.8
51.0
40.3
33.0
27.8
26.7
26.6
24.5
4al
f1 (ppm)

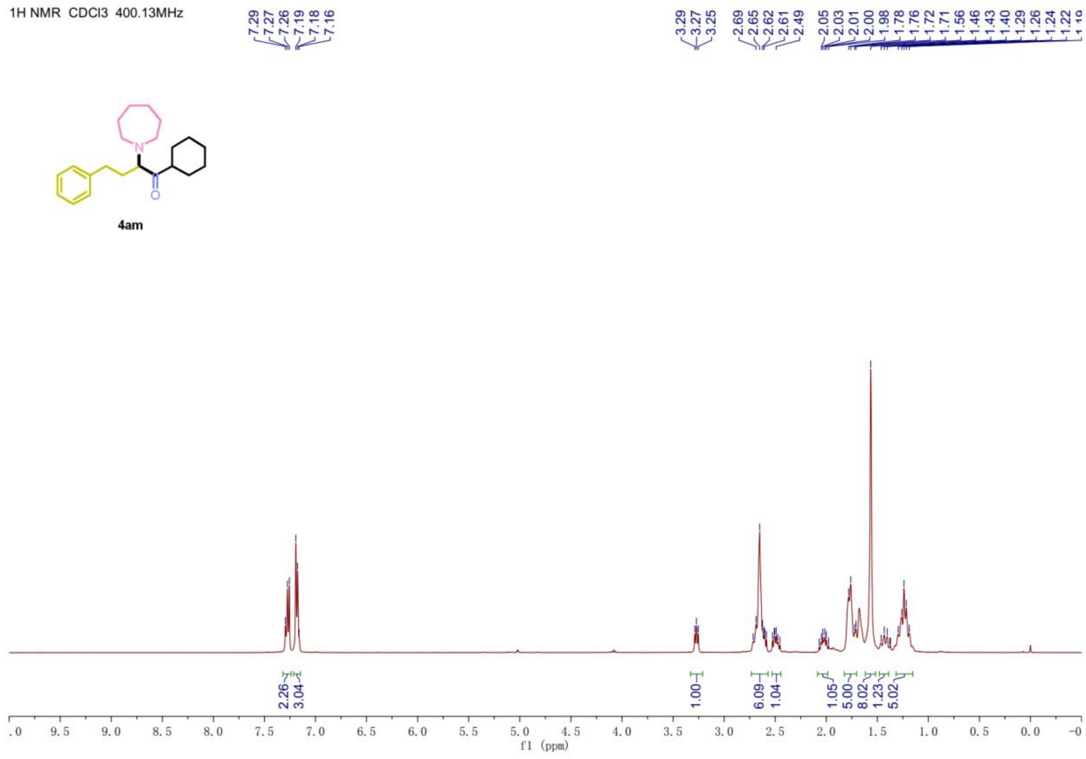
1H NMR CDCl3 400.13MHz
7.29
7.27
7.26
7.19
7.18
7.16
3.29
3.27
3.25
2.69
2.65
2.62
2.61
2.49
2.05
2.03
2.01
2.00
1.98
1.78
1.76
1.72
1.71
1.56
1.46
1.43
1.40
1.29
1.26
1.24
1.22
4am
2.26
3.04
1.00
6.09
1.04
1.05
5.00
8.02
1.23
5.02
f1 (ppm)

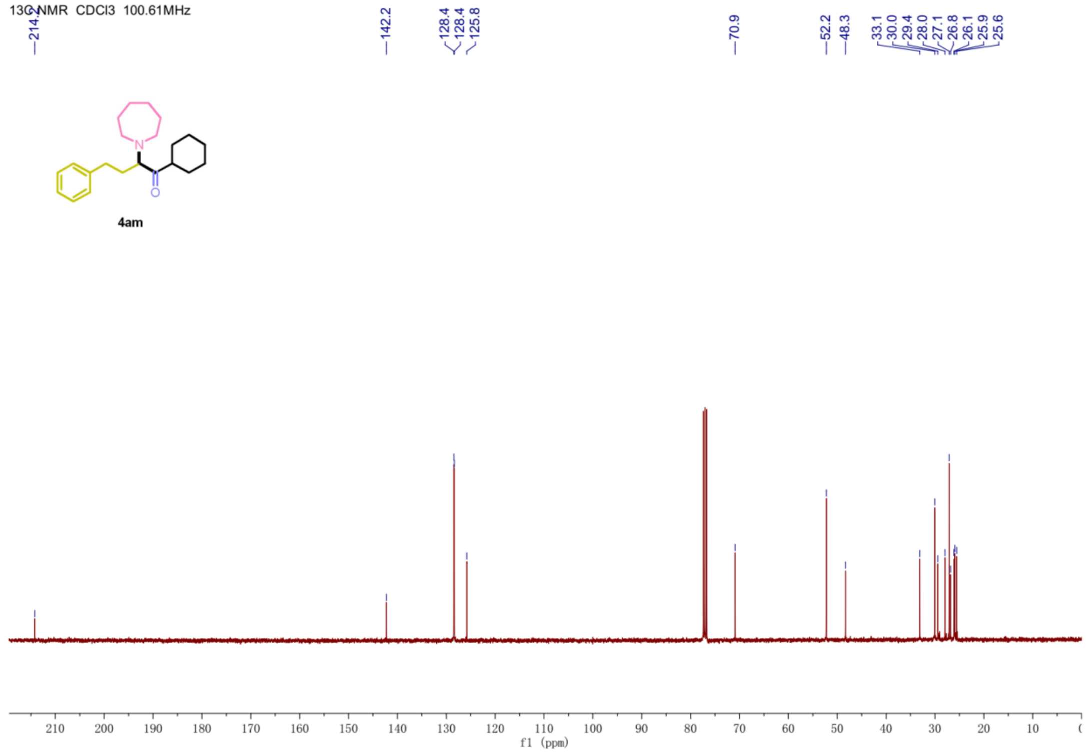
13C NMR CDCl3 100.61MHz
214.1
142.2
128.4
128.4
125.8
70.9
52.2
48.3
33.1
30.0
29.4
28.0
27.1
26.8
26.1
25.9
25.6
4am
f1 (ppm)

1H NMR CDCl3 400.13MHz

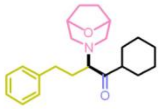

4an

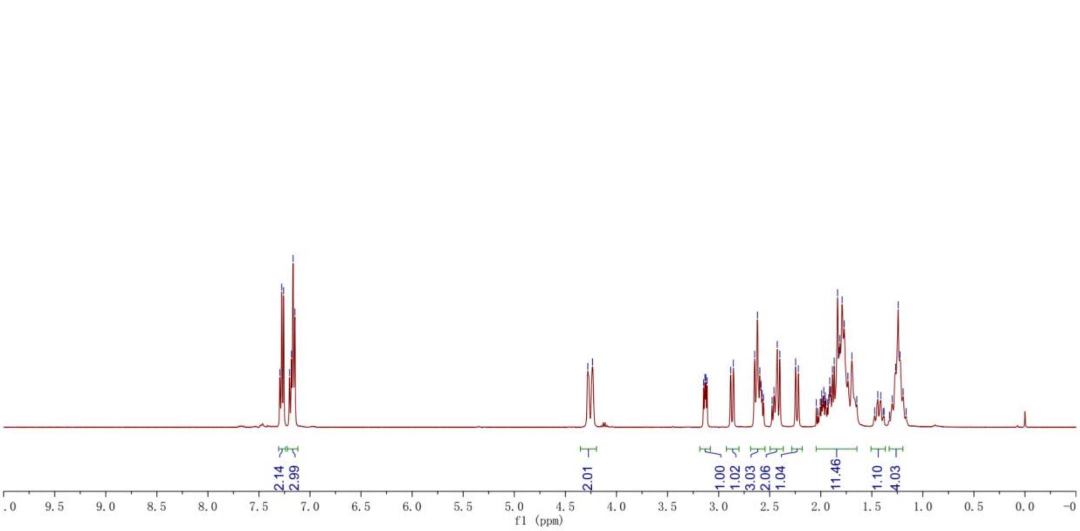

13C NMR CDCl3 100.61MHz

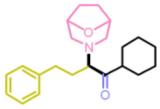

4an

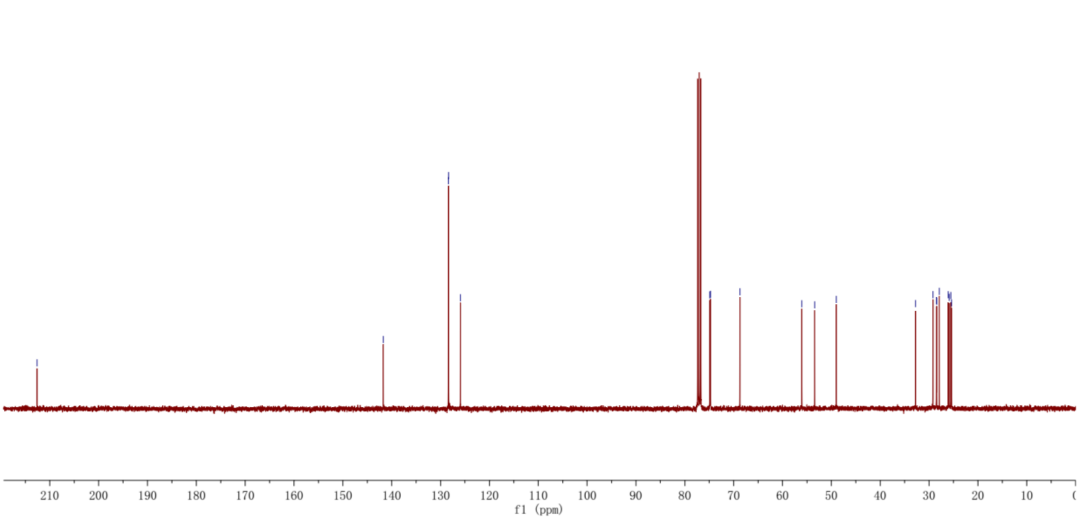

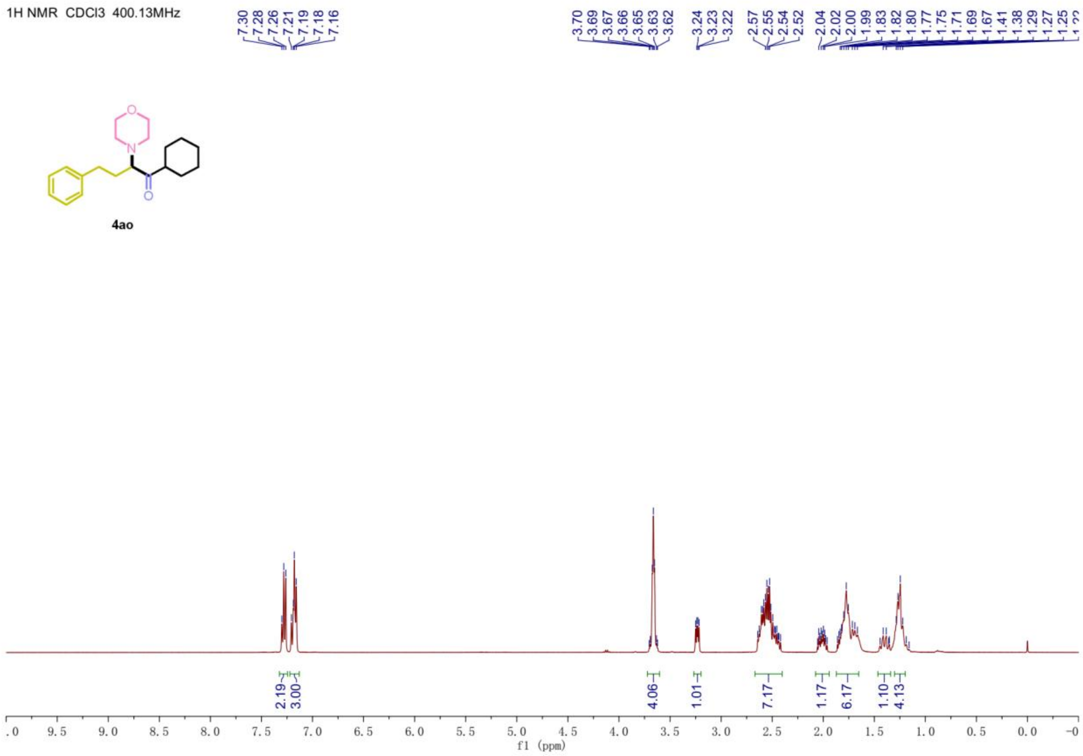
1H NMR CDCl3 400.13MHz
7.30
7.28
7.26
7.21
7.19
7.18
7.16
3.70
3.69
3.67
3.66
3.65
3.63
3.62
3.24
3.23
3.22
2.57
2.55
2.54
2.52
2.04
2.02
2.00
1.99
1.83
1.82
1.80
1.77
1.75
1.71
1.69
1.67
1.41
1.38
1.29
1.27
1.25
O
N
4ao
2.19
3.00
4.06
1.01
7.17
1.17
6.17
1.10
4.13
f1 (ppm)

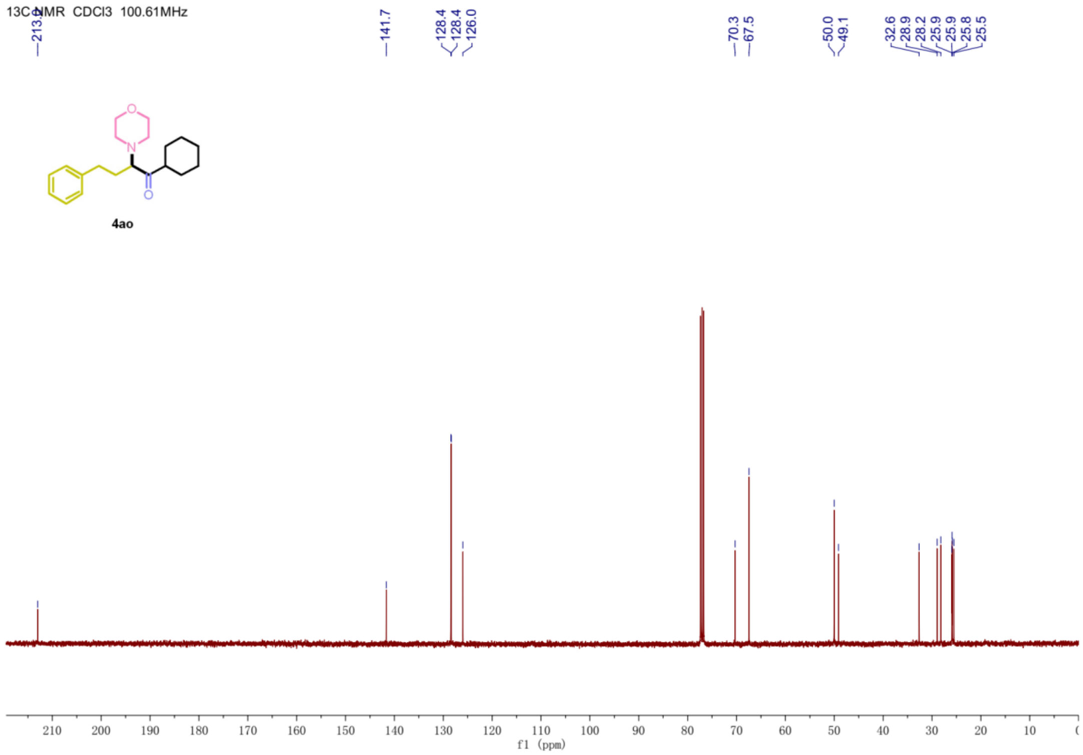
13C NMR CDCl3 100.61MHz
141.7
128.4
128.4
126.0
70.3
67.5
50.0
49.1
32.6
28.9
28.2
25.9
25.8
25.5
O
N
4ao
f1 (ppm)

1H NMR CDCl3 400.13MHz

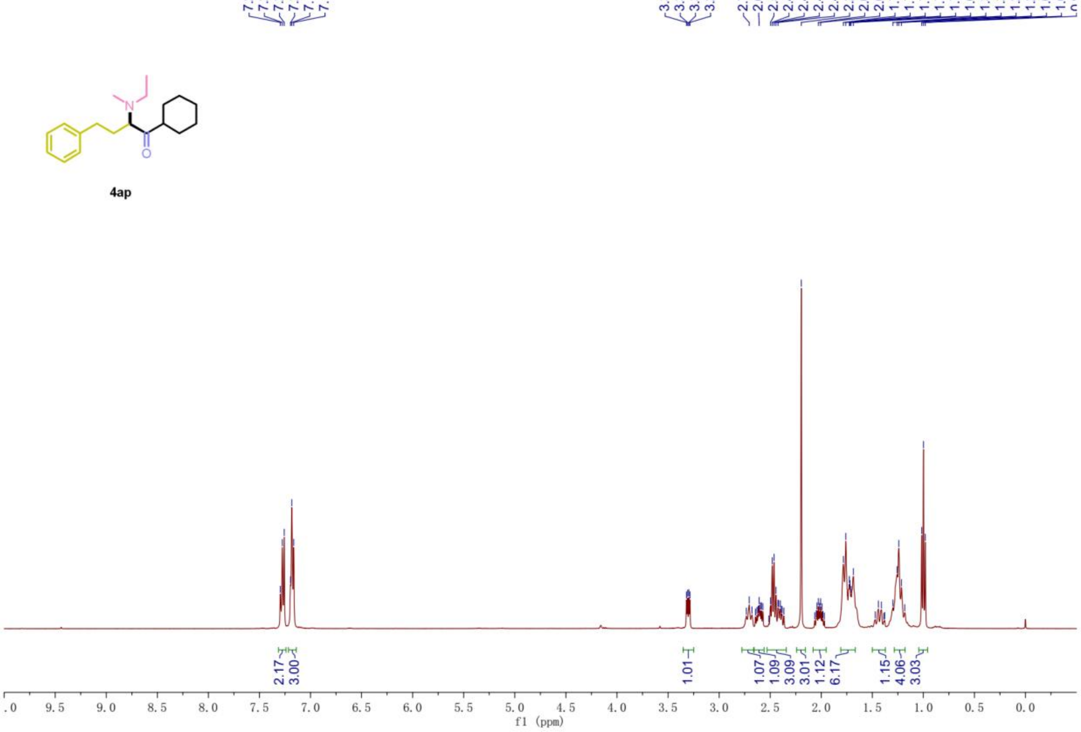

13C NMR CDCl3 100.61MHz

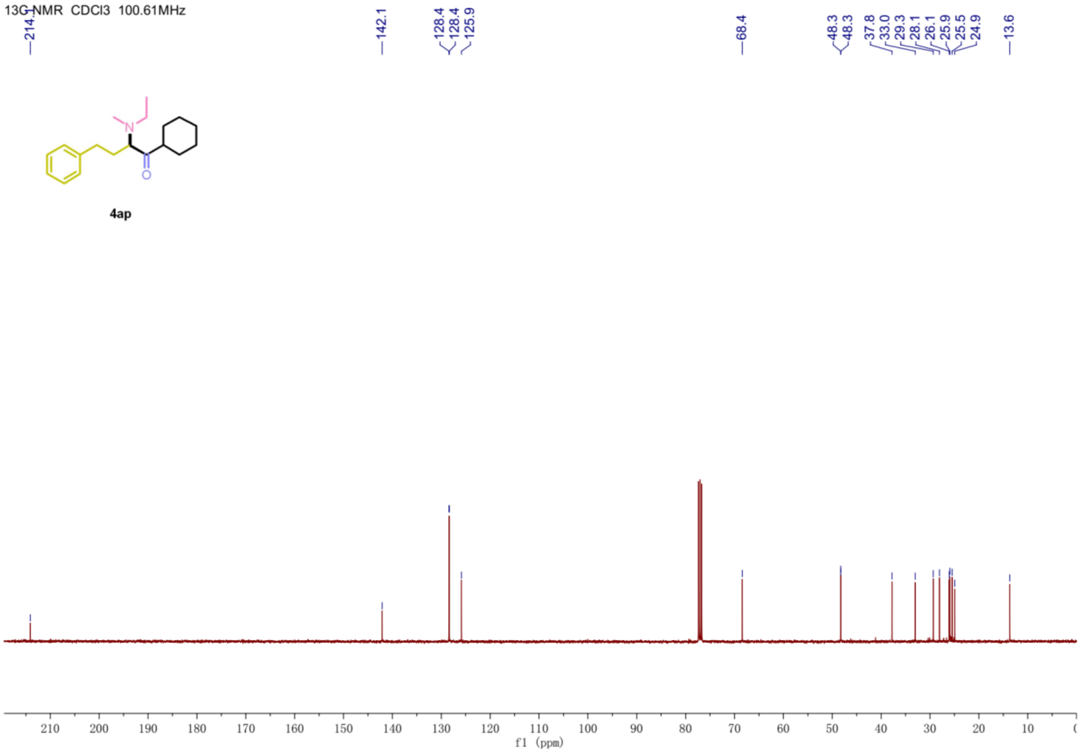

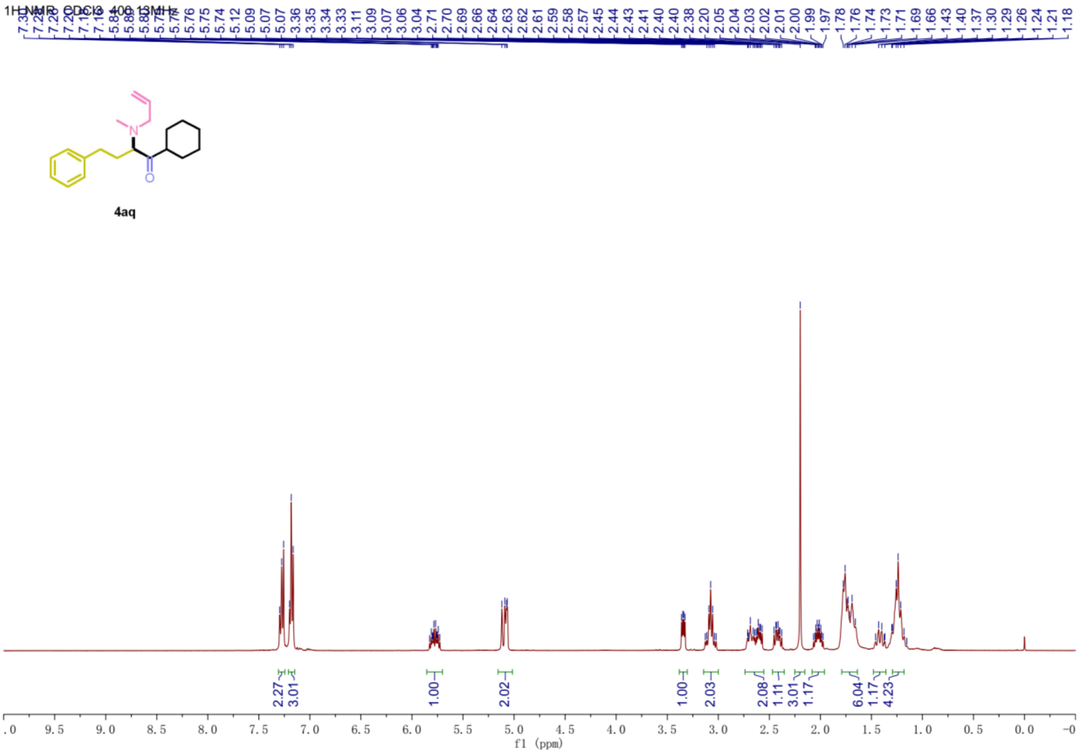
1H NMR CDCl3 400.13MHz
5.76 5.75 5.74 5.12 5.09 5.07 5.07 3.36 3.35 3.34 3.33 3.11 3.09 3.07 3.06 3.04 2.71 2.70 2.69 2.66 2.64 2.63 2.62 2.61 2.59 2.58 2.57 2.45 2.44 2.43 2.41 2.40 2.40 2.38 2.20 2.05 2.04 2.03 2.02 2.01 2.00 1.99 1.97 1.78 1.76 1.74 1.73 1.71 1.69 1.66 1.43 1.40 1.37 1.30 1.29 1.26 1.24 1.21 1.18
4aq
2.27 3.01 1.00 2.02 1.00 2.03 2.08 1.11 3.01 1.17 6.04 1.17 4.23
f1 (ppm)

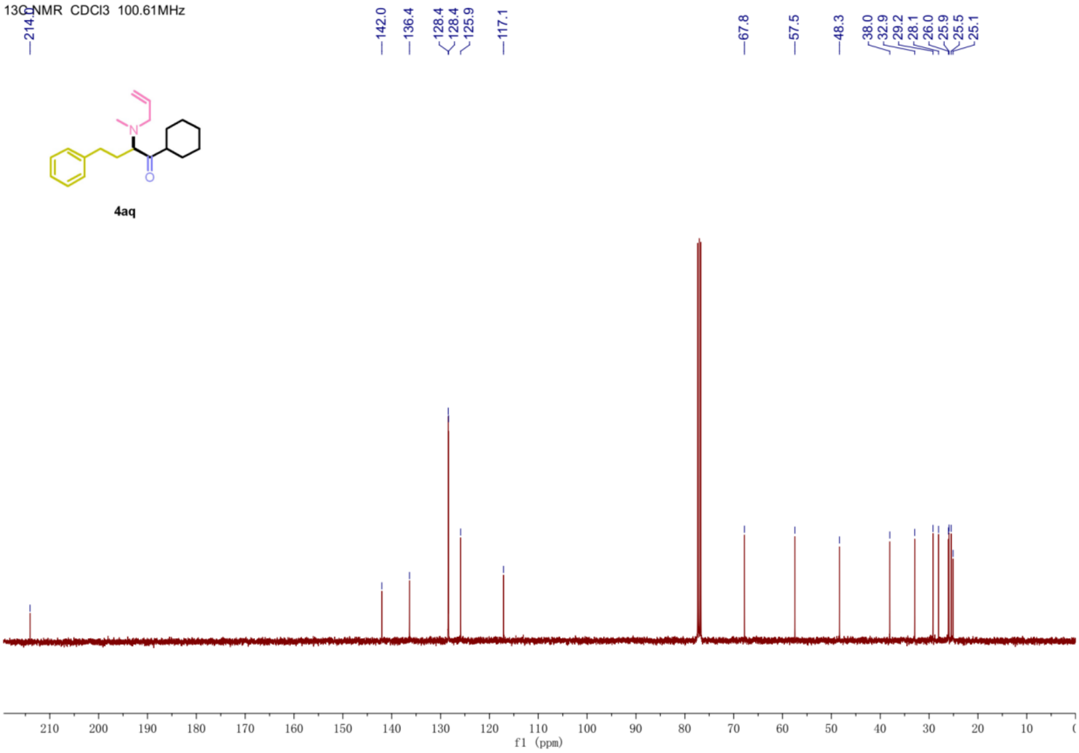
13C NMR CDCl3 100.61MHz
214.1 142.0 136.4 128.4 128.4 125.9 117.1 67.8 57.5 48.3 38.0 32.9 29.2 28.1 26.0 25.9 25.5 25.1
4aq
f1 (ppm)

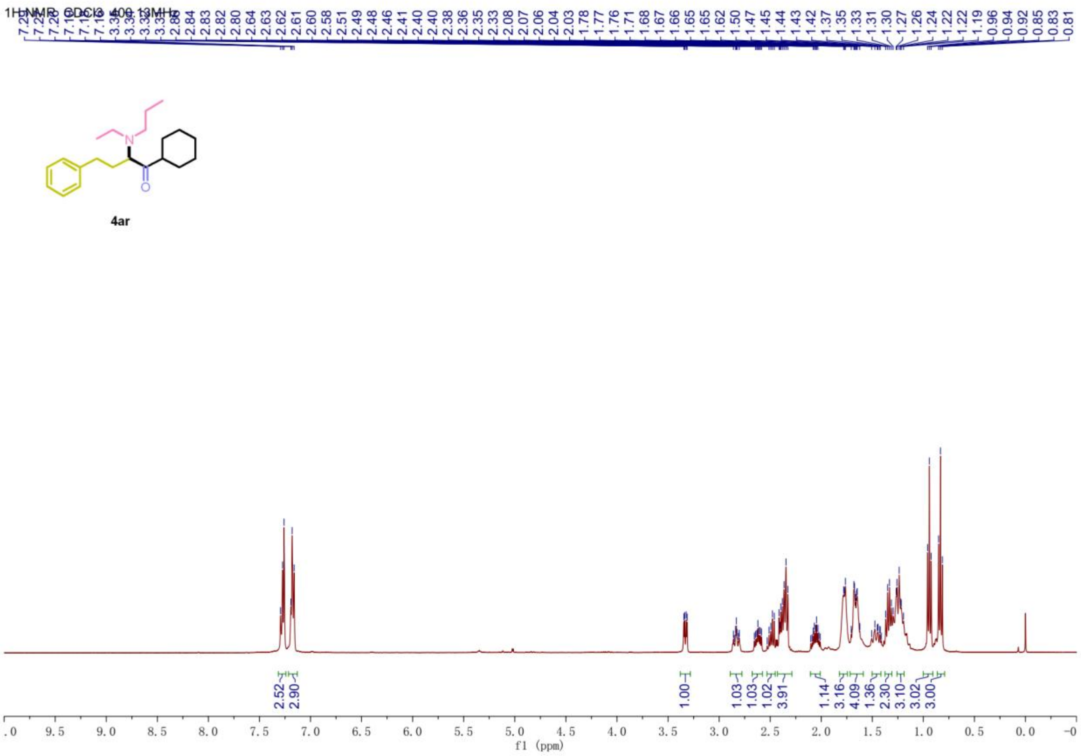
1H NMR CDCl3 400.13MHz
4ar
2.52
2.90
1.00
1.03
1.03
1.02
3.91
1.14
3.16
4.09
1.36
2.30
3.10
3.02
3.00
f1 (ppm)

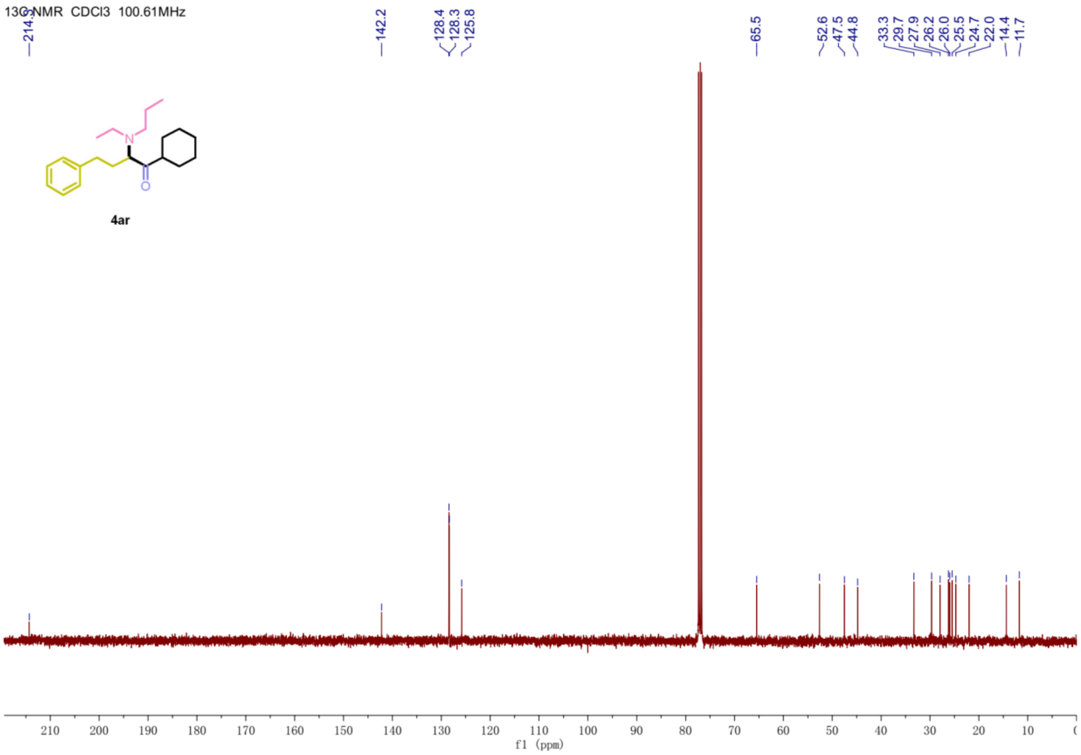
13C NMR CDCl3 100.61MHz
214.2
142.2
128.4
128.3
125.8
65.5
52.6
47.5
44.8
33.3
29.7
27.9
26.2
26.0
25.5
24.7
22.0
14.4
11.7
4ar
f1 (ppm)

1H NMR CDCl3 400.13MHz

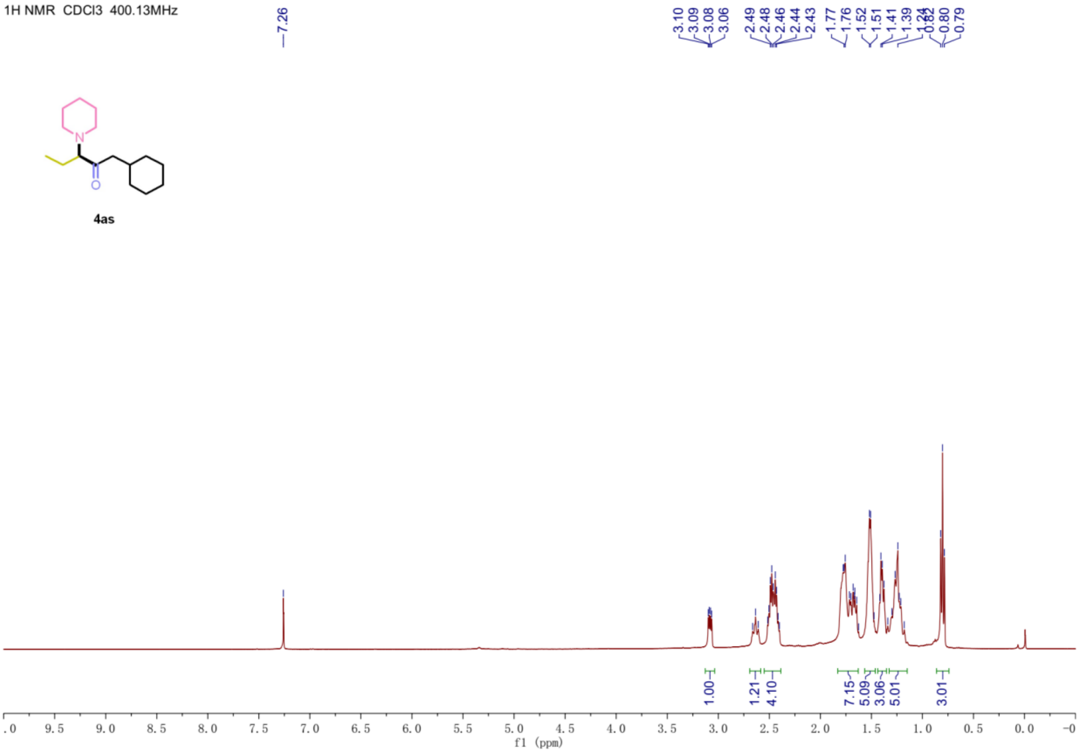

13C NMR CDCl3 100.61MHz

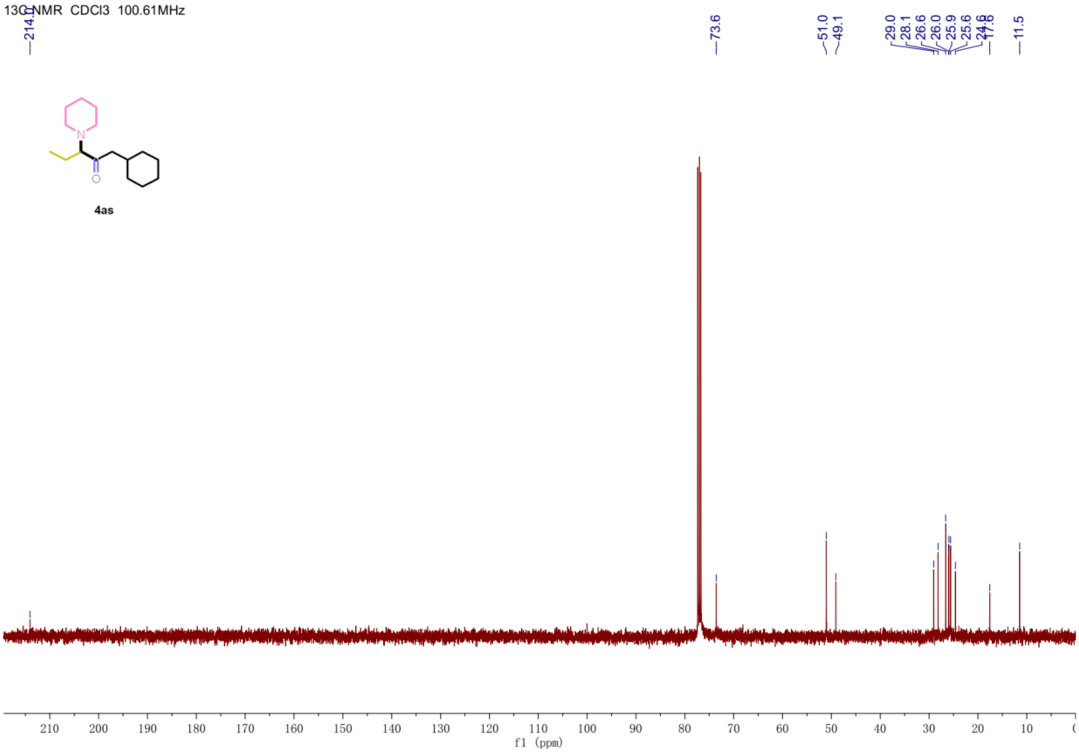

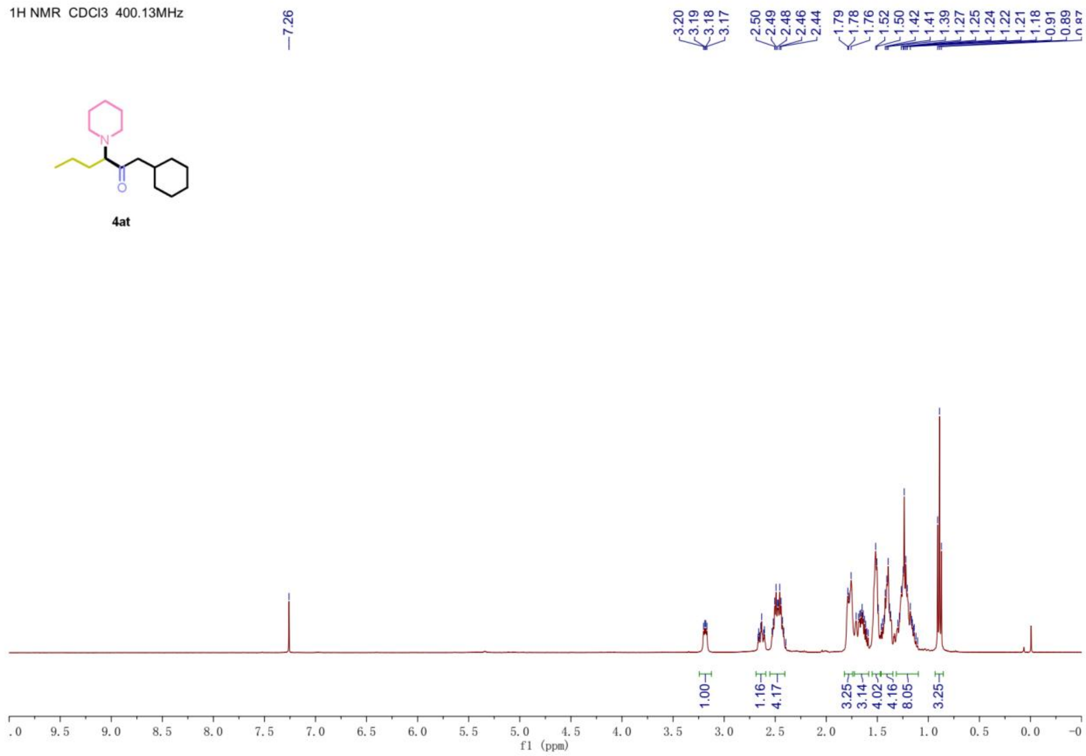
1H NMR CDCl3 400.13MHz
7.26
3.20
3.19
3.18
3.17
2.50
2.49
2.48
2.46
2.44
1.79
1.78
1.76
1.52
1.50
1.42
1.41
1.39
1.27
1.25
1.24
1.22
1.21
1.18
0.91
0.89
N
O
4at
1.00
1.16
4.17
3.25
3.14
4.02
4.16
8.05
3.25
f1 (ppm)

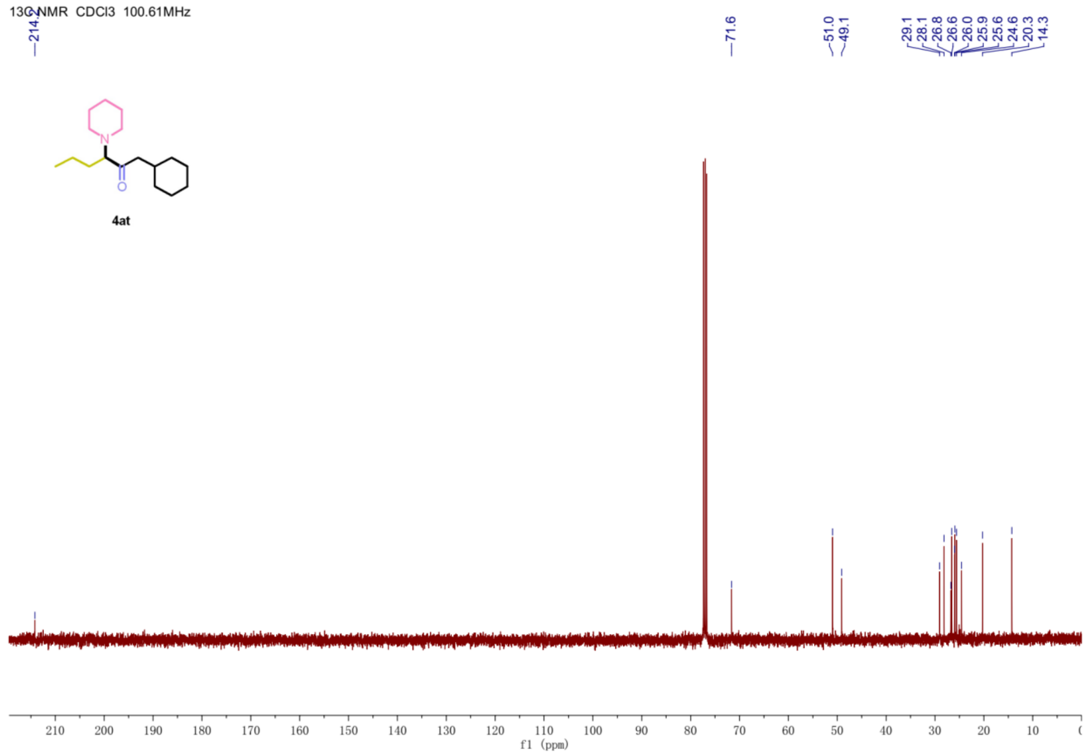
13C NMR CDCl3 100.61MHz
214.1
71.6
51.0
49.1
29.1
28.1
26.8
26.6
26.0
25.9
25.6
24.6
20.3
14.3
N
O
4at
f1 (ppm)

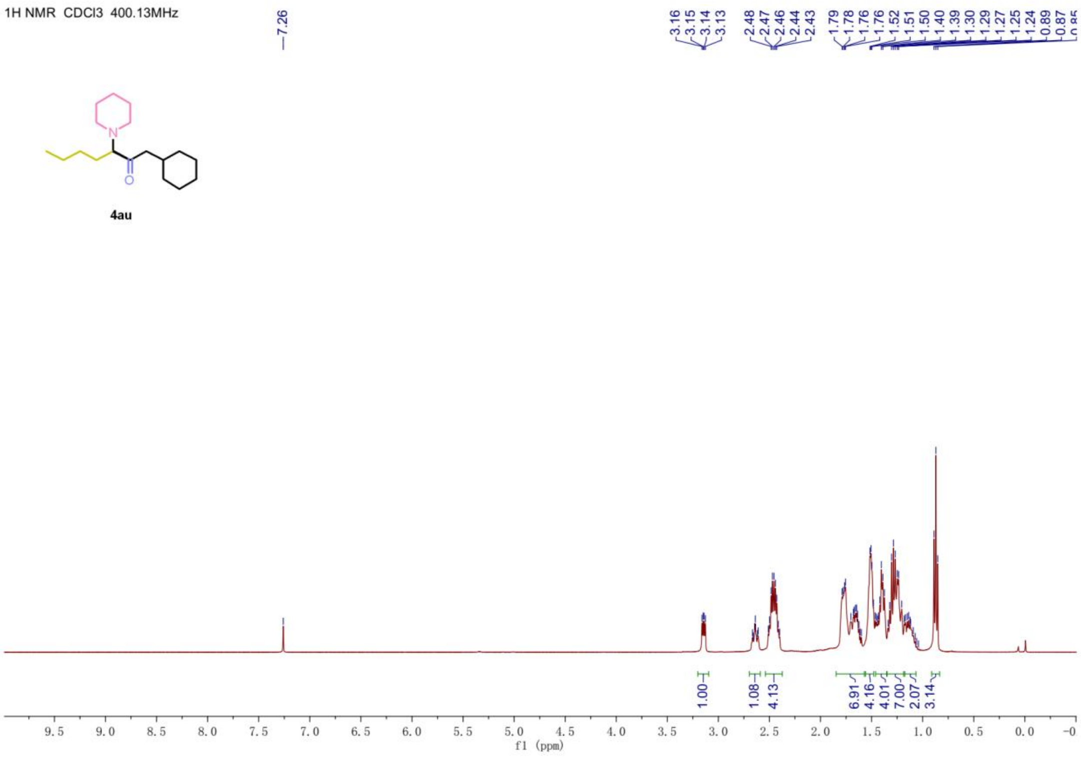
1H NMR CDCl3 400.13MHz
4au
7.26
3.16
3.15
3.14
3.13
2.48
2.47
2.46
2.44
2.43
1.79
1.78
1.76
1.76
1.52
1.51
1.50
1.40
1.39
1.30
1.29
1.27
1.25
1.24
0.89
0.87
1.00
1.08
4.13
6.91
4.16
4.01
7.00
2.07
3.14
f1 (ppm)

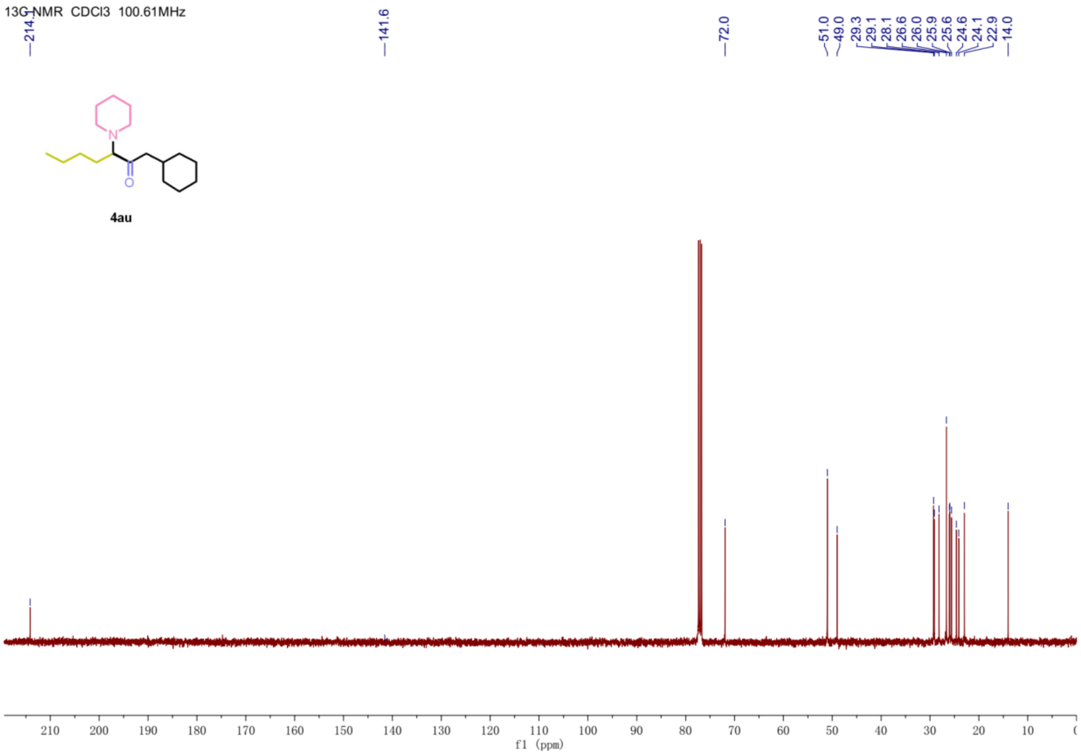
13C NMR CDCl3 100.61MHz
4au
214.1
141.6
72.0
51.0
49.0
29.3
29.1
28.1
26.6
26.0
25.9
25.6
24.6
24.1
22.9
14.0
f1 (ppm)

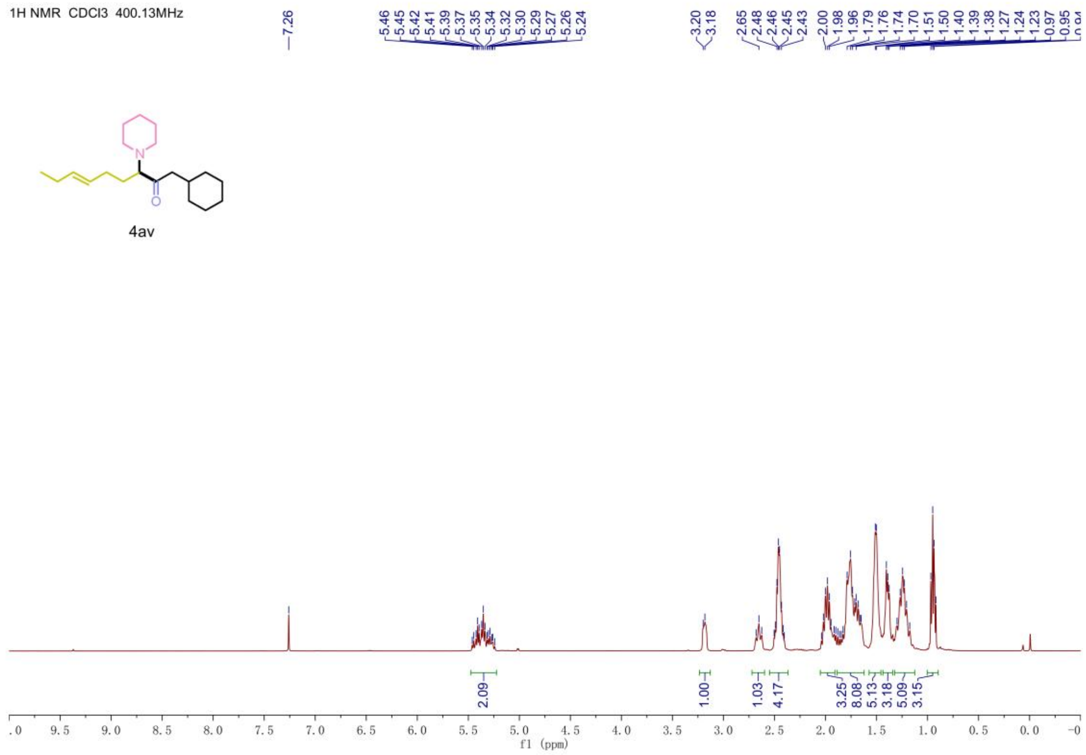
1H NMR CDCl3 400.13MHz
7.26
5.46 5.45 5.42 5.41 5.39 5.37 5.35 5.34 5.32 5.30 5.29 5.27 5.26 5.24
3.20 3.18
2.65 2.48 2.46 2.45 2.43
2.00 1.98 1.96 1.79 1.76 1.74 1.70 1.51 1.50 1.40 1.39 1.38 1.27 1.24 1.23 0.97 0.95
N
O
4av
2.09
1.00
1.03
4.17
3.25
8.08
5.13
3.18
5.09
3.15
f1 (ppm)

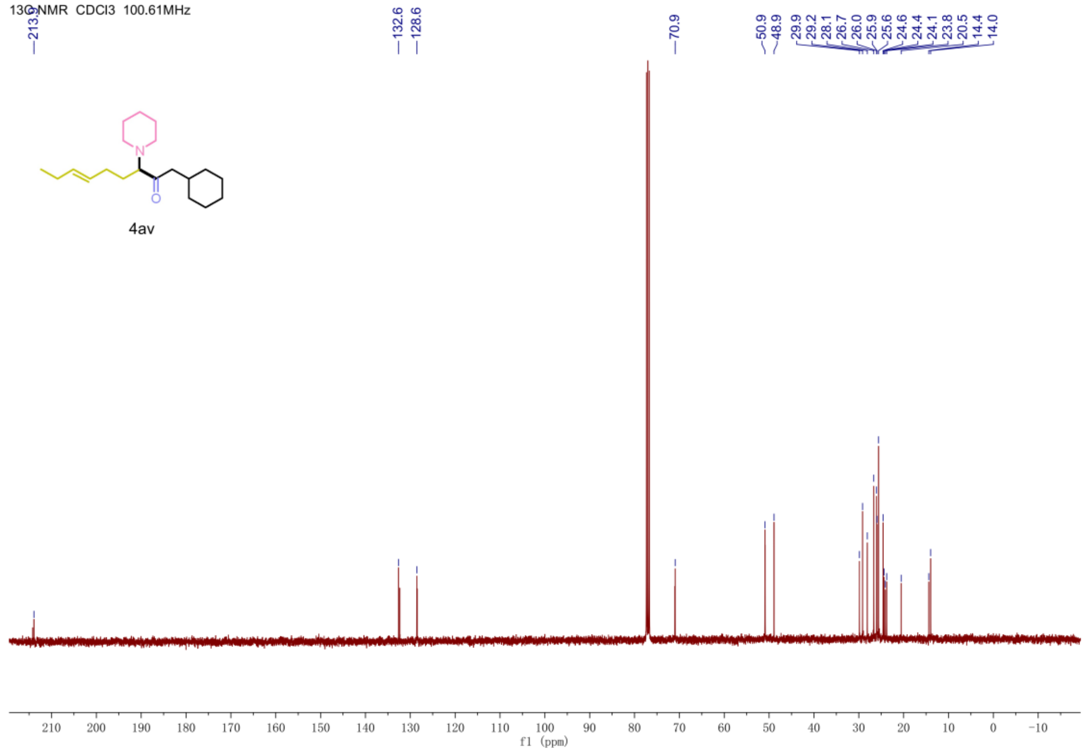
13C NMR CDCl3 100.61MHz
132.6
128.6
70.9
50.9
48.9
29.9 29.2 28.1 26.7 26.0 25.9 25.6 24.6 24.4 24.1 23.8 20.5 14.4 14.0
N
O
4av
f1 (ppm)

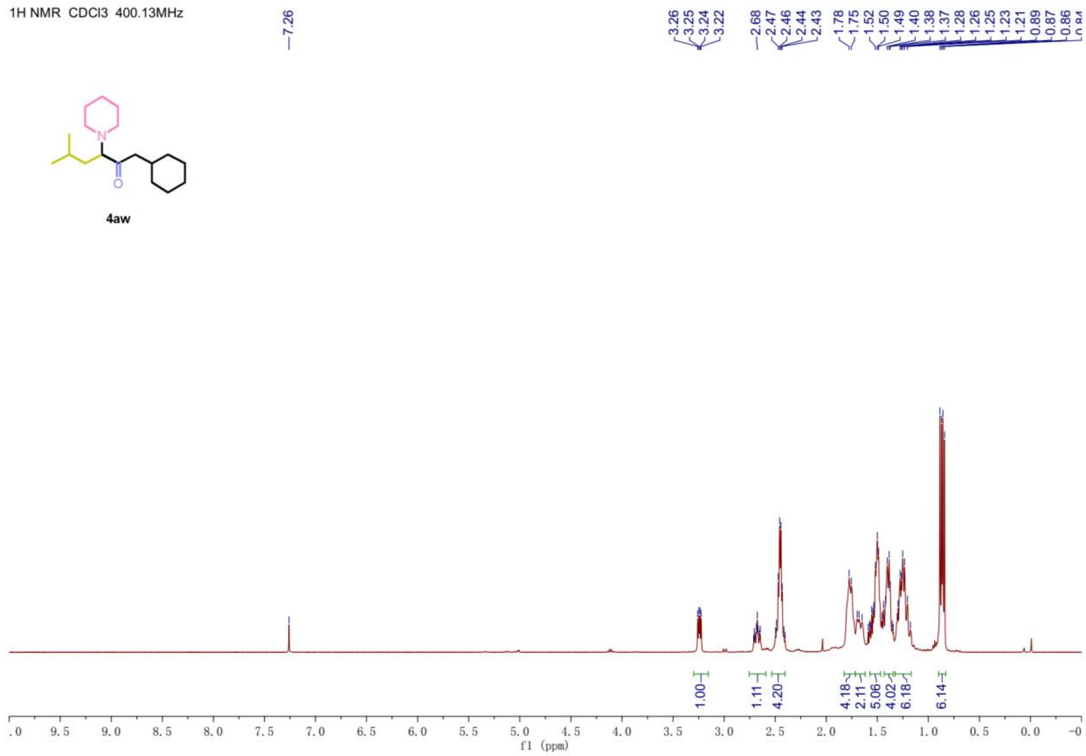
1H NMR CDCl3 400.13MHz
7.26
3.26
3.25
3.24
3.22
2.68
2.47
2.46
2.44
2.43
1.78
1.75
1.52
1.50
1.49
1.40
1.38
1.37
1.28
1.26
1.25
1.23
1.21
0.89
0.87
0.86
N
O
4aw
1.00
1.11
4.20
4.18
2.11
5.06
4.02
6.18
6.14
f1 (ppm)

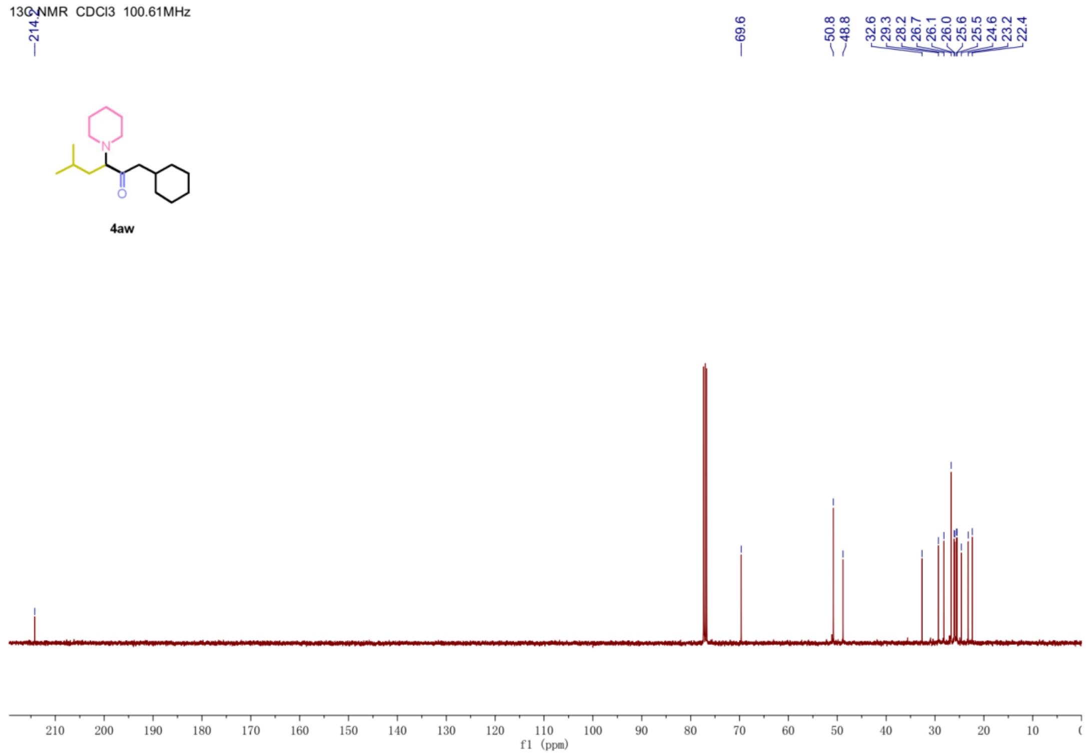
13C NMR CDCl3 100.61MHz
69.6
50.8
48.8
32.6
29.3
28.2
26.7
26.1
26.0
25.6
25.5
24.6
23.2
22.4
N
O
4aw
f1 (ppm)

1H NMR CDCl3 400.13MHz

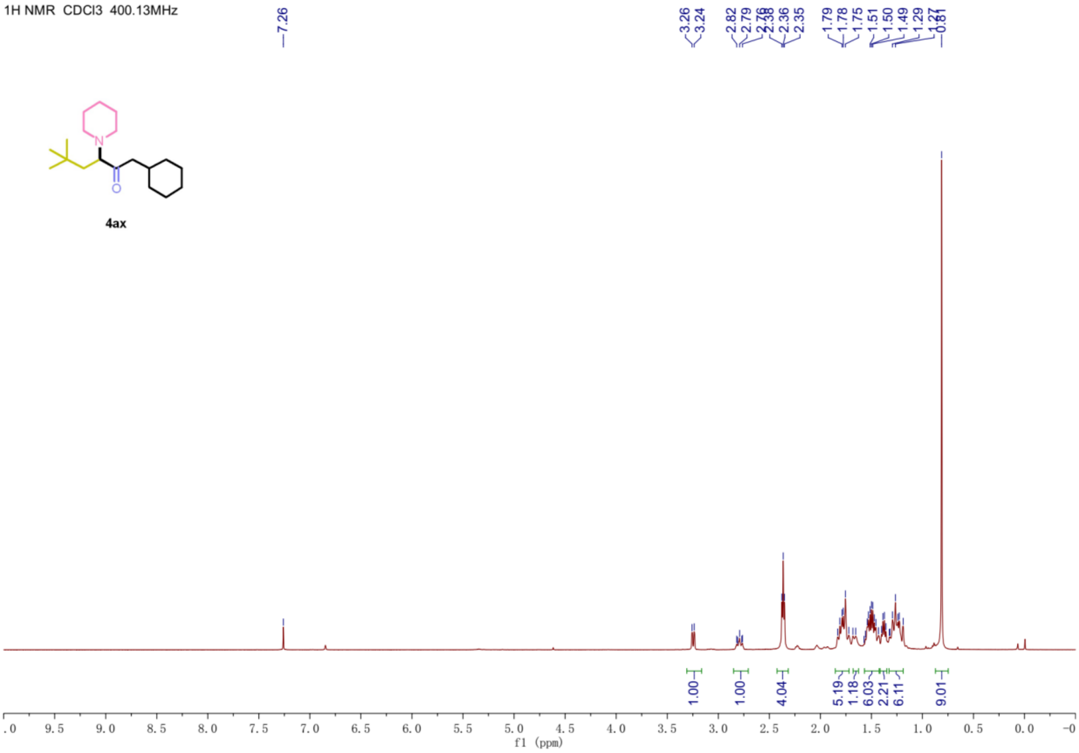

13C NMR CDCl3 100.61MHz

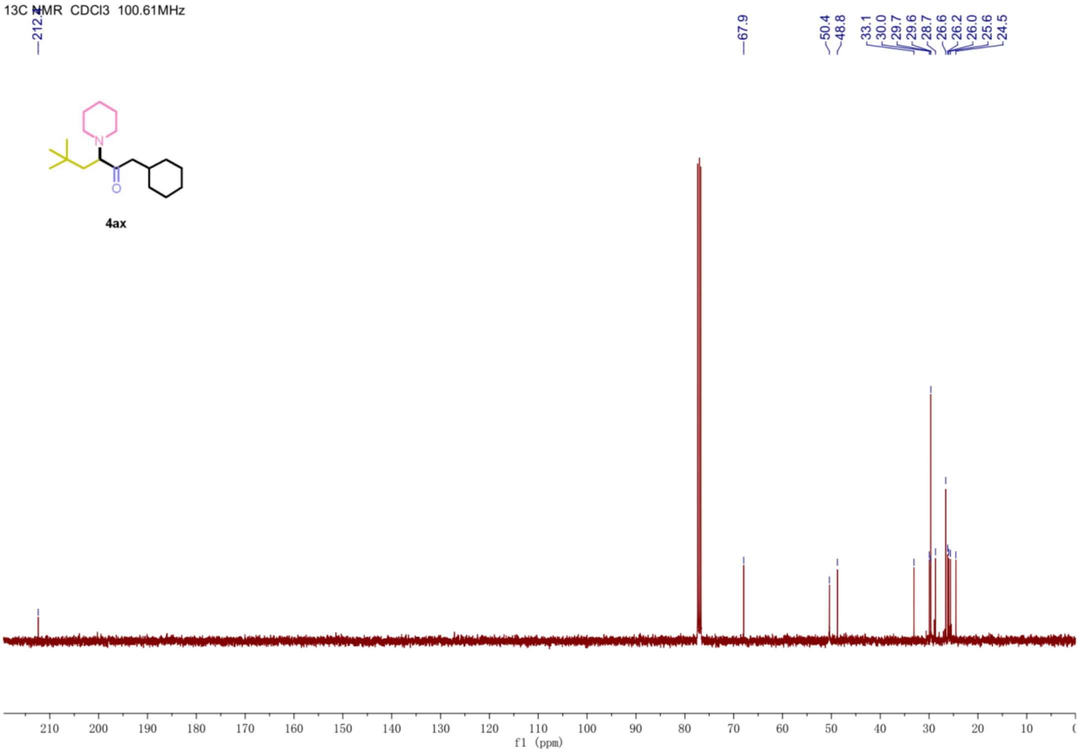

Supplement: Supplementary file 1 [file ol6c00604_si_001.pdf]
